# Characteristics of Listeria Monocytogenes Strains Persisting in a Meat Processing Facility over a 4-Year Period

**DOI:** 10.3390/pathogens8010032

**Published:** 2019-03-07

**Authors:** Andrea Stoller, Marc J. A. Stevens, Roger Stephan, Claudia Guldimann

**Affiliations:** Institute for Food Safety and Hygiene, Vetsuisse Faculty, University of Zurich, 8057 Zurich, Switzerland; andrealaura.stoller@uzh.ch (A.S.); marc.stevens@uzh.ch (M.J.A.S.); stephanr@fsafety.uzh.ch (R.S.)

**Keywords:** *Listeria monocytogenes*, meat processing facility, persistence, benzalkonium chloride, peracetic acid, biofilm

## Abstract

*Listeria monocytogenes* can persist in food production facilities, resulting in serious threats to consumers due to the high mortality associated with listeriosis, especially in the very young, old and pregnant. We subtyped 124 strains of *L. monocytogenes* isolated from a meat processing facility in Switzerland by serotyping, multi locus sequence typing (MLST) typing and whole genome sequencing. We then analyzed their ability to form biofilms and their resistance to the disinfectants benzalkonium chloride (BC) and peracetic acid (PAA). The genotyping results of the strains showed that several clonal populations of *L. monocytogenes* belonging to CC9, CC204 and CC121 had persisted in this meat processing facility for at least four years. All of the strains showed biofilm forming capacity comparable to a known high biofilm forming strain. Known efflux pumps for BC were present in CC204, CC9 (*brcABC*) and CC121 (*qacH*) strains, while strains from other CC showed very low minimal inhibitory concentrations (MICs) for BC. For PAA, minimal bactericidal concentrations of 1.2–1.6% for 20 min and minimal inhibitory concentrations between 0.1 and 0.2% were observed. These values were close to or above the recommended concentration for use (0.5–1%), suggesting that PAA might be ineffective at controlling *L. monocytogenes* in this and potentially other meat processing facilities.

## 1. Introduction

*Listeria monocytogenes* is a food-borne pathogen that causes disease mainly in vulnerable populations such as very young, old, pregnant or immunocompromised individuals. The high mortality rate of 15–30 deaths per 100 cases of listeriosis [1,2,3,4] is mostly attributable to severe central nervous system infections, septicemia, abortions and neonatal listeriosis. In the United States alone, listeriosis is estimated to cause an annual loss of 8800 disease adjusted live years (DALY), of which the majority are premature deaths [5].

Human cases of listeriosis often trace back to food products that were contaminated during production, with subsequent growth of *L. monocytogenes* to high numbers. Ready-to-eat products such as salads or deli meat are of special concern due to the lack of a heating step prior to consumption. The frequent occurrence of *L. monocytogenes* in the environment results in a high probability of introducing the organism into facilities, either on raw materials, through equipment or via employees. Once introduced, several factors increase the probability of a strain being able to establish long-lasting colonization of niches. *L. monocytogenes* has a high tolerance against acid and salt stress, and is able to grow at refrigerating temperatures. In addition, the ability to form biofilms may enhance survival, especially in niches that are difficult to reach during cleaning procedures. Further, tolerance against commonly used disinfectants such as the quaternary ammonium chloride compound benzalkonium chloride were observed in *L. monocytogenes* isolates from food processing environments [6,7,8,9,10,11]. Accordingly, *L. monocytogenes* presents a challenge to the food industry and has been shown to persist in food producing facilities for long periods of time, in some cases for more than a decade. For example, a meat factory in Texas harbored the same strain for at least 12 years, eventually causing an outbreak in 2000 [6,7]. A smoked fish processing plant harbored the same strain of *L. monocytogenes* for 11 years [8]. A study in Ireland found that seven out of 48 food processors housed a persistent strain—defined as isolated at least six months apart [9]. 

Here, we analyzed strains from a Swiss deli meat plant where *L. monocytogenes* strains were repeatedly isolated between 2015 and 2018. The aim was (i) to determine whether a clonal population of *L. monocytogenes* persisted in the facility or if *L. monocytogenes* was repeatedly reintroduced, and (ii), to characterize the resistance of the collected strains against benzalkonium chloride (BC) and peracetic acid (PAA) and their ability to form biofilms. 

## 2. Results

### 2.1. Subtyping of 124 Listeria Monocytogenes Isolated from a Swiss Meat Plant

A total of 124 strains of *L. monocytogenes* were analyzed in this study. All strains were collected in the framework of a hygiene monitoring program in a meat processing facility in Switzerland between 2015 and 2018 (Table 1). The collection contains four strains from 2015, three strains from 2016, 32 strains from 2017 and 85 strains from 2018. The samples originated from products (n = 8) and from the food production environment (n = 116).

In a first screening of the diversity of the 124 *L. monocytogenes* strains, their serogroup and multi locus sequence typing (MLST) profile was determined. The majority of the strains (n = 73, 58.9%) belonged to serogroup II (containing serotype 1/2c, 3c), 48 strains (38.7%) belonged to serogroup I (1/2a, 3a) and three strains (2.4%) belonged to serogroup IV (4b, 4d, 4e). Seven gene MLST revealed that all of the 73 serogroup II strains belonged to CC9 (n = 73, 58.9%). The strains belonging to serogroup I were more heterogenous with 31 strains assigned to CC204, seven strains assigned to CC121, four strains to CC20 and to CC29, and one to CC8 and to CC89. The three strains in serogroup IV all belonged to CC6. Only strains from CC9, CC204 and CC121 were consistently isolated over all four years. CC6 strains seem to have been introduced into the facility in July 2017 and persisted until the end of the sampling period in June 2018. 

From these results we concluded that it was likely that clonal populations of CC9, CC204, CC121, and CC6 persisted in this facility, while strains from other complexes were sporadically introduced and deemed “presumably non-persistent”. We consciously use the term “presumably” because there is no reasonable way to determine if those strains would have persisted in different environments or under different growth conditions. 

**Figure 1:**
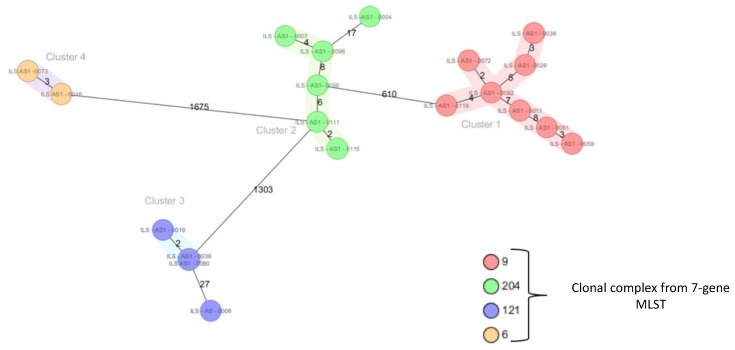
**Core genome MLST** (cgMLST) for 20 selected strains from the collection. See main text for details.

In a next step, the genomes of 20 strains were sequenced and a core genome MLST (cgMLST) comparison was performed (Figure 1). The cgMLST revealed that most strains from the same CC differed in less than 10 alleles, a cutoff for strain clonality [10]. 

### 2.2. Biofilm Formation

The capacity to form biofilms is an important mechanism for persistence [11], and biofilm formation might have contributed to the maintained presence of clonal clusters of *L. monocytogenes* strains in the facility for years. We therefore investigated the biofilm forming ability in a subset of strains of the collective (CC9, CC204, CC121 and CC6) at 22 and 8 °C.

**Figure 2:**
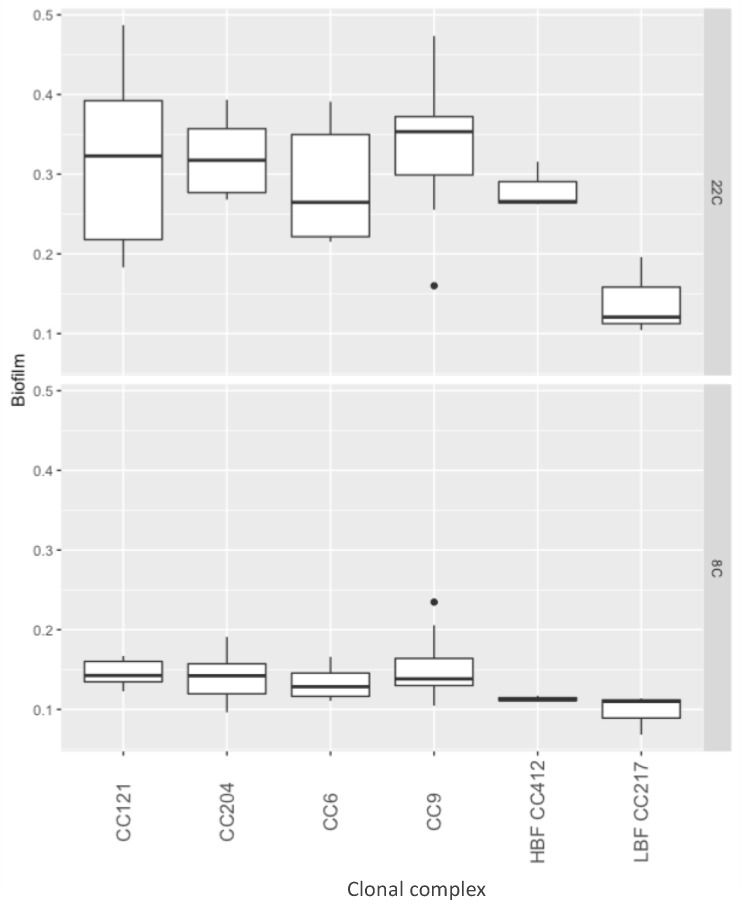
Biofilm formation by clonal complex, at 22 and 8 °C. The y-axis represents optical density in a crystal violet assay, the x-axis represents strains by clonal complex.

Approximately double the biofilm mass was recovered after 96 h of growth at 22 °C compared to 168 h growth at 8 °C (p < 0.01) (Figure 2). However, no individual strain had significantly different biofilm formation compared to the other tested strains (Supplementary File 1). 

At 22 °C, strains from CC9, CC204, CC121 and CC6 showed a biofilm forming capacity in the range of a high biofilm forming (HBF) control strain, and all strains had significantly higher biofilm formation than that of a low biofilm forming (LBF) control strain (p < 0.01). At 8 °C, all strains, including the HBF control strain, were impaired in their biofilm forming capacity and hence, no significant differences between the strains from different complexes, nor between the strains isolated from the meat processing facility vs. the control strains, were found (Figure 2). 

### 2.3. Tolerance to Benzalkonium Chloride 

**Figure 3:**
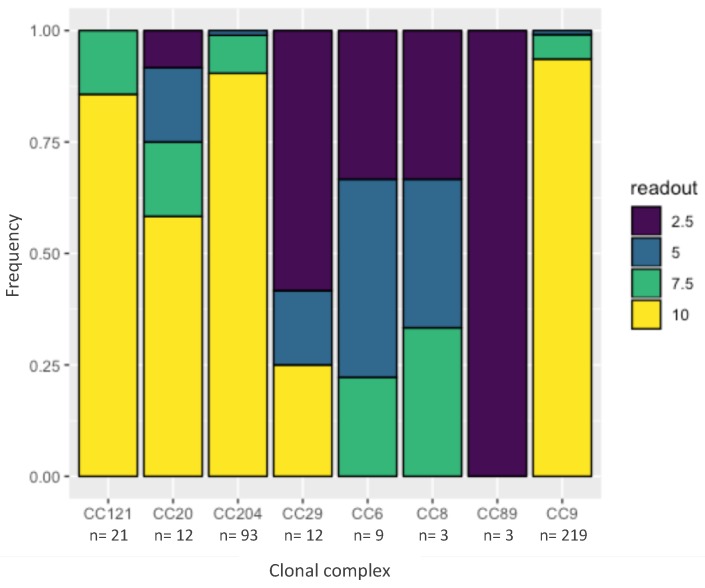
**Minimal inhibitory concentration** (MIC) of benzalkonium chlorice (BC) by clonal complex. The y-axis represents frequency. The colors represent the highest concentration of BC (in μg/mL) at which confluent growth was observed after 48 h at 37 °C. The x-axis represents the different clonal complexes. n is the number of data points that were observed for each clonal complex.

Benzalkonium chloride is a commonly used disinfectant in the food industry and resistance against BC might contribute to the persistence of a strain. One hundred and six out of 124 tested strains were resistant to 10 µg/mL BC (Figure 3). None of the strains, however, were resistant to the cutoff for resistance—20 µg/mL BC. A cumulative link model [12] revealed that CC29, CC89, CC8 and CC6 had a significantly lower tolerance to BC (combined average of 4.5 µg/mL BC) compared to the other CC in the dataset (combined average of 9.2 µg/mL BC, p < 0.05). 

A genome-wide search in the 20 sequenced strains revealed the presence of either *brcABC* [13] or *qacH* [14] BC resistance genes in 16 (80%) of the strains (Table 1). *brcABC* genes were present in six strains of CC204 and CC9, respectively. *qacH* was present in four CC121 strains. No strain carried the *emrE* [15] BC resistance gene. All strains that carried resistance genes had the highest measured BC resistance at 10 µg/mL, except strain ILS AS1-0004 which was resistant to 7.5 µg/mL. 

### 2.4. Minimal Inhibitory Concentration and Minimal Bactericidal Concentration of Peracetic Acid against L. monocytogenes

PAA is frequently used as a disinfectant in the food production environment with a recommended final concentration between 0.5% and 1%. The meat processing facility from which the strains originated used 0.8% PAA as a sanitizer; we therefore hypothesized that strains from this collective might have adapted to PAA over time. 

**Figure 4:**
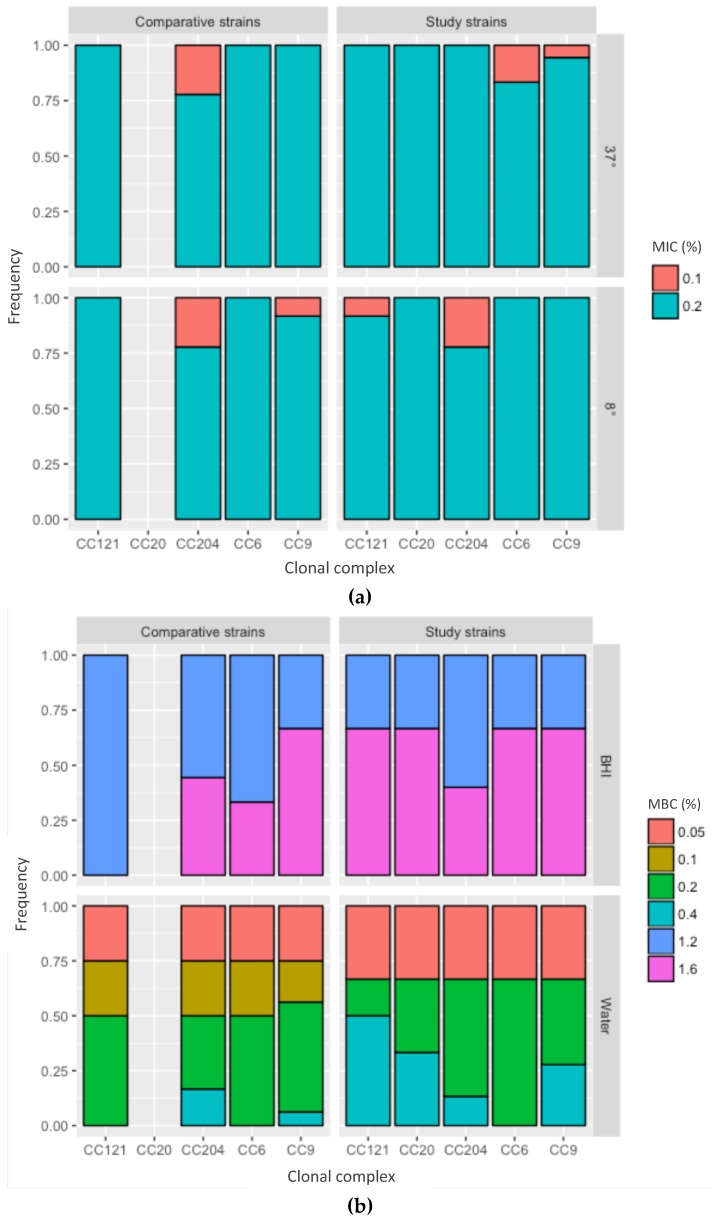
Tolerance to peracetic acid (PAA). The x-axis represents the clonal complexes, the y-axis the frequency. Study strains—strains isolated from the food processing facility; comparative strains—strains from the same CC but isolated from unrelated sources. (**a**) Minimal inhibitory concentration (MIC) for PAA in BHI, at 37 and 8 °C. (**b**) Minimal bactericidal concentration (MBC) for PAA by medium, BHI—brain heart infusion; water—tap water.

The MIC was between 0.1% and 0.2% for all strains and the MBC was between 1.2% and 1.6% (Figure 4, Supplementary File 1). Since the strains were isolated up to four years apart, these data strongly suggest no adaptation to PAA over time.

All further analyses were performed in a subset of strains that were chosen to represent all four years of collection and were divided over the most frequent clonal complexes CC9 (n = 6), CC204 (n = 4), CC121 (n = 3), CC6 (n = 2) and CC20 (n = 1). To test whether the strains had an unusually high resistance to PAA, a comparison was made to *L. monocytogenes* strains from unrelated sources that belonged to the same clonal complexes [CC9 (n = 4), CC204 (n = 3), CC121 (n = 1) and CC6 (n = 1)] (Table 1). The minimal bactericidal concentration (MBC) of the strains was between 1.2% and 1.6% with no difference between the clonal complexes or between the strains isolated from the meat processing facility versus the control strains (Figure 4a). Therefore, the strains from the meat processing facility showed no increased tolerance to PAA.

To test whether the availability of protein compounds in the test medium affected the outcome, the MBC was compared with PAA in tap water and PAA in brain heart infusion (BHI). The MBC of PAA in tap water was 0.1–0.4% and, therefore, lower compared to the PAA BHI (p < 0.05) (Figure 4b), indicating that protein affects the effectiveness of PAA and that there is a protein error for PAA. 

We further assessed whether the incubation temperature influenced the outcome, which would indicate that PAA has a cold error. While MBC values were generally a better indicator for disinfection, we used MIC measurements to address this question because they allowed us to test the effects of cold exposure over a longer time than MBC measurements. The MIC for PAA was 0.2% for all strains, regardless of incubation at 4 °C or 37 °C, suggesting no evidence for a cold error for PAA against *L. monocytogenes.*

## 3. Discussion

In this study, we showed that several clonal populations of *L. monocytogenes* persisted in a meat processing facility in Switzerland for at least four years. The persistent strains belonged to CC9, CC204 and CC121. The dominant CC9 and CC121 have previously been shown to be overrepresented in food processing facilities in France [16], Spain [17], Switzerland [18], and all over Europe [19]. CC204 on the other hand has only been sporadically isolated from food processing facilities [18] and human patients [20]. 

Strains from the facility formed biofilm in the range of a high biofilm former. However, no difference was found between the individual strains isolated from the facility, nor between clonal complexes. Subtle differences between clonal complexes might be revealed by replacing the 96-well format biofilm screening protocol used in this study [21] with more labor-intensive procedures, such as culturing biofilms on stainless steel coupons [22], in a follow-up study. Contradictory results have been found by other authors: Some studies showed that persistent strains of *L. monocytogenes* formed more biofilm than presumed non-persistent strains [11], and persistent strains were more efficient in attaching to surfaces during a short contact time [23]. Others did not observe a difference between persistent vs. presumed non-persistent strains [24], and the ability of *L. monocytogenes* to form true biofilms at all has been critically questioned by some authors [25]. Given the high biofilm forming capacity we found in the strains of *L. monocytogenes* isolated from this meat processing facility, biofilm formation may have contributed to persistence. 

The overrepresentation of CC121 in food processing environments has often been explained with their higher resistance to BC due to the presence of the *qacH* or *brcABC* genes that encode efflux pumps [26]. Confirming this, all CC121 strains that were sequenced in this study were positive for *qacH*. Moreover, 80% of all sequenced strains carried either *qacH* or *brcABC*. Given the clonal structure of the CC121, CC9 and CC204 strains in this collection and their uniform resistance to 10 µg/mL BC, it is reasonable to assume that most strains in this collection carried either *qacH* or *brcABC*. However, tolerance to 10 µg/mL BC is below the typical in-use concentrations of BC (500–1000 µg/mL) [27], indicating that BC should be active against the strains in this study. 

In contrast, PAA was routinely used for disinfection in the facility, at a final concentration of 0.8%. Our data showed that the MBC (1.2–1.6%) and the MIC (0.1–0.2%) values were close to or above this concentration, to ensure efficient disinfection. Experiments without nutrients in the medium showed that even in the absence of proteins the MBC, between 0.2% and 0.4%, was still very close to the recommended concentration for use. Under real-life conditions, when disinfecting larger areas, it is likely that the concentration of the disinfectant becomes diluted through residual washing water and that residual organic matter is present. It is also likely that contact times differ due to run-off or delayed reach of hard to clean niches, which in this case would lead to ineffective concentrations of PAA. 

Different strains of *L. monocytogenes* also exhibited high MBCs for PAA in the range of up to 0.5% [28,29,30,31]. In contrast, others concluded that PAA was able to significantly reduce *L. monocytogenes* from multispecies biofilms at 0.15% [32] and 0.3% [30], respectively. The MIC of PAA for other organisms seemed to be much lower, in the range of 0.01–0.03% for Gram-positive and Gram-negative flora isolated from citrus fruit [33], 0.0003% for *Escherichia coli, Staphylococcus aureus, Pseudomonas aeruginosa* [34], and 0.0001–0.001% for *P. aeruginosa*, *S. aureus*, *E. coli*, and *S. epidermidis* [35]. Given the high tolerance of *L. monocytogenes* against PAA found in our data and by others, the ability of PAA to reduce *L. monocytogenes* from biofilms may primarily have been due to the elimination of the supporting flora in multispecies biofilms.

Our data confirm the absence of adaptation of *L. monocytogenes* to PAA found in other work that found no adaptation over several hundred generations when *L. monocytogenes* were exposed to an industrial disinfectant containing PAA and hydrogen peroxide [36]. 

Taken together, these experiments show that the concentration of 0.8% PAA used by the facility is insufficient to ensure safe production standards with regard to *L. monocytogenes*. However, PAA is a valuable option for disinfection due to its effectiveness against most bacteria, fungi and viruses, the absence of a cold error, its status as GRAS, and its colorless and odorless properties, but only when *L. monocytogenes* is not a major concern. Since PAA at concentrations above 1% is corrosive to equipment and irritating to the eyes, via fumes, an alternative disinfectant regimen should be considered in food production facilities that struggle to control *L. monocytogenes* in their environment. 

## 4. Material and Methods

### 4.1. Bacterial Strains and Preparation of Bacterial Cultures

The hygiene-monitoring program of the meat-producing facility entailed product samples and swabs of the production environment such as floor drains, trolleys, elevators, cold room floors, scales, production and packaging lines, toilet drains and toilet floors or doors between production sections. All samples were tested for *L. monocytogenes* using the “Assurance Genetic Detection System” (GDS, Biocontrol, Nieuwerkerk aan den IJssel, The Netherlands) according to the protocol. In short, swabs or samples were incubated in Half Frazer Broth (HFB, BioRad, Marne-la-Coquette, France) at 30 °C for 48 h. *L. monocytogenes* were further enriched on magnetic beads and then identified via a kit-specific PCR in an “Assurance GDS Rotor-Gene” cycler. To obtain single colonies, the enriched HFB was streaked on Oxoid chromogenic Listeria agar (OCLA) plates (Oxoid, Pratteln, Switzerland) and incubated at 37 °C for 24 h. All strains were kept in 15% glycerol stocks at –80 °C.

To obtain overnight cultures for experiments, the strains were streaked on BHI agar (Oxoid, Pratteln, Switzerland) and incubated overnight at 37 °C. A single colony was inoculated into 5 mL BHI and incubated for 18 h overnight at 37 °C with shaking at 200 RPM in a shaking incubator (Edmund Buehler SM30/TH30 combination, Huber AG, Reinach, Switzerland). To obtain exponential phase cultures with an OD590 of 0.4, 50 µL of the overnight culture was subcultivated into 5 mL of fresh BHI and incubated for 3 h at 37 °C with shaking at 200 rpm.

### 4.2. Serogrouping by qPCR

DNA was isolated from 1 mL overnight cultures using the DNeasy Blood and Tissue Kit (Qiagen, Hilden, Germany) and eluted in 10 mM Tris pH 7.3. The DNA concentration was measured with a Nanodrop 1000 (Thermo Fisher Scientific, Basel, Switzerland) and PCR templates were standardized to 10^5^ copies per 1 µL by dilution in fresh 10 mM Tris. Multiplex qPCRs were performed according to Vitullo et al. [37], with the following modifications: Instead of a triplex-PCR we performed the qPCR as duplex with PUC19 [38] as an internal control on the third channel. Primers and probes according to Vitullo et al. (37) (Table 2) were obtained from Sigma-Aldrich (Buchs, Switzerland), and used at a final concentration of 0.4 μM for the primer and 0.2 μM for the probe.

Cycling conditions for the two-step PCR on a LightCycler 2.0 (Roche Science, Rotkreuz, Switzerland) were as follows: 5 s at 95 °C, followed by 40 cycles of 45 s at 95 °C and 45 s at 60 °C. 

### 4.3. Multi Locus Sequence Typing (MLST)

MLST was performed on all 124 strains according to Ragon et al. [39]. All primers (Table 2) were ordered from Microsynth (Balgach, Switzerland). The fragment sizes were confirmed by gel-electrophoresis, the products were sequenced by Microsynth (Balgach Switzerland), assembled in Geneious (Version 11.1.4, Biomatters, Newark, NJ, USA) and analyzed using the website of the Institute Pasteur (http://bigsdb.pasteur.fr/listeria/listeria.html). 

### 4.4. Whole Genome Sequencing

Based on the MLST results, a selection of 20 strains (Table 1) were Illumina sequenced. DNA was extracted, as for the MLST. Sequencing libraries were prepared using the Illumina Nextera DNA Flex chemistry and sequenced on an Illumina MiniSeq (Illlumina, San Diego, CA, USA) with a minimal coverage of 30 ×. After quality control with FastQC (http://www.bioinformatics.babraham.ac.uk/projects/fastqc/), the reads were assembled with Spades 3.12.0 [40]. Core-genome multilocus sequence typing (cgMLST) was performed in the software package SeqSphere 4.1.9 (Ridom, Münster, Germany). Assembled genome sequences were imported and blasted against 1701 genes of the seed genome EGD-e, using the standard settings [10]. A minimal spanning tree was produced in SeqSphere with the options “ignore missing values pairwise” and “discard genomes with >3% missing genes”. Strains with less than 10 different alleles were considered to belong to the same complex.

### 4.5. Biofilm Formation

The 15 strains that represented the four clonal complexes that seemed to persist in the factory (CC9 (n = 9), CC204 (n = 2), CC121 (n = 2)), (CC6 (n = 2)) were chosen for biofilm assays, according to the protocol published by Harvey et al. with minor changes [21]. A single colony was inoculated into 5 mL of tryptone soy broth (TSB, from Fluka, obtained from Sigma-Aldrich, Buchs, Switzerland), incubated for 20 h at 30 °C with shaking at 200 rpm, subcultured 1:250 into fresh TSB, and incubated for an additional 20 h at 30 °C with shaking at 200 rpm. The resulting cultures were adjusted to an OD_600_ of 1.0, diluted 1:80 in TSB and added to 96-well plates, which were incubated for 96 h at 22 °C, or for 168 h at 8 °C, respectively. Biofilms were then washed three times with distilled water, stained with crystal violet, and washed five times with distilled water. The remaining crystal violet was dissolved in ethanol and the OD_600_ was measured in a Synergy plate reader (BioTek, Lucern, Switzerland). Control strains that were high and low biofilm formers (Institute for Food Safety and Hygiene, Zurich; unpublished results) (Table 1) were included in each experiment. 

### 4.6. Tolerance to Benzalkonium Chloride 

MICs for BC were determined for all 124 strains included in this study, as per the protocol of Meier et al. with minor changes [26]. Five µL of an exponential phase culture were spotted on BHI plates containing BC at 0, 2.5, 5.0, 7.5, 10.0, 15.0, 20.0, 25.0 and 30.0 µg/mL (Sigma Aldrich, Buchs, Switzerland). The plates were incubated for 48 h at 37 °C. Strains were considered resistant to the concentration of BC on which confluent growth was observed. The cutoff for resistance was set, according to Langsrud et al. [41], as double the value of the lowest concentration that inhibited growth in >50% of the tested strains.

All sequenced genomes were searched for the *qacH*, *brcABC* and *emrE* BC resistance genes using BLASTP 2.7.1+ [42] with the standard settings and an e-value cutoff of 10^−20^. 

### 4.7. Minimal Inhibitory Concentration and Minimal Bactericidal Concentration of Peracetic Acid against L. monocytogenes

These assays were performed in PAA diluted in BHI to mimic a worst case scenario that assumed incomplete cleaning of organic matter from surfaces before disinfection, and to assess the overall effect of PAA on bacteria within an otherwise favorable environment. To determine the influence of the protein in BHI on the outcome, some of the assays were additionally performed in PAA diluted in tap water (City of Zürich, supplementary file 2). First, the MIC and MBC for PAA were determined in a screening of all 124 strains. Given the clonal nature of much of the strain collection, we then compared the resistance to PAA in detail in a subset of the strains: CC9 (n = 6), CC204 (n = 4), CC121 (n = 3), CC6 (n = 2) and CC20 (n = 1). A control dataset contained strains from unrelated sources: CC9 (n = 4), CC204 (n = 3), CC121 (n = 1) and CC6 (n = 1) (Table 1).

To obtain the MBC values, serial dilutions were prepared to obtain final PAA concentrations of 2.8%, 2.4%, 2.0%, 1.6%, 1.2%, 0.8%, 0.4%, 0.2%, 0.1% and 0.05%. Next, 190 µL of these dilutions were added to 96-well plates and cooled to 4 °C. Then, exponential phase cultures were diluted in 0.9% NaCl and 10 µL were added to each well to achieve an inoculum of approximately 5 × 10^2^ CFU/well for the dilution rows in BHI and 5 × 10^4^ CFU/well for the dilution rows in water. The plates were incubated for 20 min at 4 °C. After incubation, the wells were mixed by pipetting and 20 µL were washed in 180 µL 0.9% NaCl in a fresh 96-well plate. These plates were centrifuged at 3220 g for 5 min, the supernatant was discarded, and the cells were resuspended in 20 µL 0.9% NaCl. Next, 10 µL of each well was spotted on the edge of a BHI agar plate and run down the plate by tilting [43]. These plates were incubated for either 7 days at 8 °C, to mimic the conditions in a food processing plant, or for 48 h at 37 °C, to provide more favorable growth conditions. Surviving bacteria were enumerated by direct colony count and the MBC was defined as the concentration of PAA that produced no colonies. To determine the MIC, the serial dilution plates were incubated at 37 °C for 48 h and at 8 °C for 7 days. The MIC was defined as the concentration of PAA that allowed for no visible growth [44]. 

### 4.8. Statistical Analysis

All of the experiments were performed in triplicate unless otherwise indicated.

The results were analyzed in RStudio Version 1.1.456 (RStudio, Boston, MA, USA)) and all statistical analyses are provided as a supplementary file (Supplementary File 1). In short, a linear mixed effects model, using lmer in a LmerTest [45], was modelled to the biofilm data and lsmeans was used to create contrasts [46]. A cumulative link model was calculated for the BC and PAA data using polR in MASS [47], and model selection was done with stepAIC in MASS [47]. All graphics were done using ggplot2 [48]. 

## Figures and Tables

**Table 1:** *L. monocytogenes* strains used in this study.

Strain	Isolation Date	Source	Purpose	Serogroup	Sequence Type	CC	WGS	*brcABC*	*qacH*	*emrE*	Lineage
ILS AS1-0001	2015/10/15	FPE	Study strain	2	9	9	1	absent	absent	absent	II
ILS AS1-0002	2015/10/19	FP	Study strain	1	121	121		n/a	n/a	n/a	II
ILS AS1-0003	2015/11/9	FPE	Study strain	1	204	204		n/a	n/a	n/a	II
ILS AS1-0004	2015/11/14	FPE	Study strain	1	204	204	2*	present	absent	absent	II
ILS AS1-0005	2016/2/2	FPE	Study strain	2	9	9		n/a	n/a	n/a	II
ILS AS1-0006	2016/3/9	FPE	Study strain	1	121	121	3*	absent	present	absent	II
ILS AS1-0007	2016/10/20	FP	Study strain	1	204	204	2	present	absent	absent	II
ILS AS1-0008	2017/1/9	FPE	Study strain	2	9	9		n/a	n/a	n/a	II
ILS AS1-0009	2017/1/9	FPE	Study strain	2	9	9		n/a	n/a	n/a	II
ILS AS1-0010	2017/1/9	FPE	Study strain	2	9	9		n/a	n/a	n/a	II
ILS AS1-0011	2017/1/9	FPE	Study strain	2	9	9	1	present	absent	absent	II
ILS AS1-0012	2017/1/9	FPE	Study strain	2	9	9		n/a	n/a	n/a	II
ILS AS1-0013	2017/1/9	FPE	Study strain	2	9	9		n/a	n/a	n/a	II
ILS AS1-0014	2017/1/9	FPE	Study strain	2	9	9		n/a	n/a	n/a	II
ILS AS1-0015	2017/6/26	FPE	Study strain	2	9	9		n/a	n/a	n/a	II
ILS AS1-0016	2017/6/29	FPE	Study strain	2	9	9		n/a	n/a	n/a	II
ILS AS1-0017	2017/7/7	FPE	Study strain	2	9	9		n/a	n/a	n/a	II
ILS AS1-0018	2017/7/7	FPE	Study strain	4	6	6	4	absent	absent	absent	I
ILS AS1-0019	2017/8/10	FP	Study strain	1	121	121	3	absent	present	absent	II
ILS AS1-0020	2017/9/12	FP	Study strain	1	204	204		n/a	n/a	n/a	II
ILS AS1-0021	2017/9/13	FP	Study strain	1	204	204		n/a	n/a	n/a	II
ILS AS1-0022	2017/9/18	FPE	Study strain	2	9	9		n/a	n/a	n/a	II
ILS AS1-0023	2017/9/18	FPE	Study strain	1	204	204		n/a	n/a	n/a	II
ILS AS1-0024	2017/11/6	FPE	Study strain	2	9	9		n/a	n/a	n/a	II
ILS AS1-0025	2017/11/6	FPE	Study strain	2	9	9		n/a	n/a	n/a	II
ILS AS1-0026	2017/11/13	FPE	Study strain	1	204	204	1	absent	absent	absent	II
ILS AS1-0027	2017/11/23	FPE	Study strain	2	9	9		n/a	n/a	n/a	II
ILS AS1-0028	2017/12/5	FPE	Study strain	2	9	9		n/a	n/a	n/a	II
ILS AS1-0029	2017/12/5	FPE	Study strain	2	9	9		n/a	n/a	n/a	II
ILS AS1-0030	2017/12/5	FPE	Study strain	2	9	9		n/a	n/a	n/a	II
ILS AS1-0031	2017/12/5	FPE	Study strain	1	204	204		n/a	n/a	n/a	II
ILS AS1-0032	2017/12/7	FPE	Study strain	1	20	20		n/a	n/a	n/a	II
ILS AS1-0033	2017/12/7	FPE	Study strain	1	20	20		n/a	n/a	n/a	II
ILS AS1-0034	2017/12/7	FPE	Study strain	2	9	9		n/a	n/a	n/a	II
ILS AS1-0035	2017/12/5	FPE	Study strain	2	9	9		n/a	n/a	n/a	II
ILS AS1-0036	2017/12/5	FPE	Study strain	2	9	9		n/a	n/a	n/a	II
ILS AS1-0037	2017/12/5	FPE	Study strain	2	9	9		n/a	n/a	n/a	II
ILS AS1-0038	2017/12/5	FPE	Study strain	2	9	9	1	absent	absent	absent	II
ILS AS1-0039	2017/12/14	FPE	Study strain	1	121	121	3	absent	present	absent	II
ILS AS1-0040	2018/1/23	FPE	Study strain	1	204	204		n/a	n/a	n/a	II
ILS AS1-0041	2018/1/23	FPE	Study strain	2	9	9		n/a	n/a	n/a	II
ILS AS1-0042	2018/1/23	FPE	Study strain	2	9	9		n/a	n/a	n/a	II
ILS AS1-0043	2018/1/23	FPE	Study strain	1	29	29		n/a	n/a	n/a	II
ILS AS1-0044	2018/1/23	FPE	Study strain	2	9	9		n/a	n/a	n/a	II
ILS AS1-0045	2018/1/25	FPE	Study strain	2	9	9		n/a	n/a	n/a	II
ILS AS1-0046	2018/1/25	FPE	Study strain	2	9	9		n/a	n/a	n/a	II
ILS AS1-0047	2018/1/25	FPE	Study strain	1	204	204		n/a	n/a	n/a	II
ILS AS1-0048	2018/1/25	FPE	Study strain	2	9	9		n/a	n/a	n/a	II
ILS AS1-0049	2018/1/25	FPE	Study strain	2	9	9		n/a	n/a	n/a	II
ILS AS1-0050	2018/1/25	FPE	Study strain	2	9	9		n/a	n/a	n/a	II
ILS AS1-0051	2018/1/25	FPE	Study strain	2	9	9		n/a	n/a	n/a	II
ILS AS1-0052	2018/1/25	FPE	Study strain	2	9	9		n/a	n/a	n/a	II
ILS AS1-0053	2018/1/25	FPE	Study strain	2	9	9		n/a	n/a	n/a	II
ILS AS1-0054	2018/1/25	FPE	Study strain	1	204	204		n/a	n/a	n/a	II
ILS AS1-0055	2018/1/25	FPE	Study strain	1	204	204		n/a	n/a	n/a	II
ILS AS1-0056	2018/1/25	FPE	Study strain	1	204	204	2	present	absent	absent	II
ILS AS1-0057	2018/1/25	FPE	Study strain	2	9	9		n/a	n/a	n/a	II
ILS AS1-0058	2018/1/25	FPE	Study strain	2	9	9		n/a	n/a	n/a	II
ILS AS1-0059	2018/1/25	FPE	Study strain	2	9	9	1	present	absent	absent	II
ILS AS1-0060	2018/1/25	FPE	Study strain	2	9	9		n/a	n/a	n/a	II
ILS AS1-0061	2018/1/25	FPE	Study strain	2	9	9		n/a	n/a	n/a	II
ILS AS1-0062	2018/1/26	FPE	Study strain	2	9	9		n/a	n/a	n/a	II
ILS AS1-0063	2018/1/26	FPE	Study strain	2	9	9		n/a	n/a	n/a	II
ILS AS1-0064	2018/1/25	FPE	Study strain	2	9	9		n/a	n/a	n/a	II
ILS AS1-0065	2018/1/25	FPE	Study strain	2	9	9		n/a	n/a	n/a	II
ILS AS1-0066	2018/1/25	FPE	Study strain	1	391	89		n/a	n/a	n/a	II
ILS AS1-0067	2018/2/6	FPE	Study strain	2	9	9		n/a	n/a	n/a	II
ILS AS1-0068	2018/2/6	FPE	Study strain	2	9	9		n/a	n/a	n/a	II
ILS AS1-0069	2018/2/7	FPE	Study strain	1	29	29		n/a	n/a	n/a	II
ILS AS1-0070	2018/2/6	FPE	Study strain	2	9	9		n/a	n/a	n/a	II
ILS AS1-0071	2018/2/9	FPE	Study strain	2	9	9		n/a	n/a	n/a	II
ILS AS1-0072	2018/2/9	FPE	Study strain	2	9	9	1	present	absent	absent	II
ILS AS1-0073	2018/2/9	FPE	Study strain	4	6	6	4	absent	absent	absent	I
ILS AS1-0074	2018/2/9	FPE	Study strain	1	29	29		n/a	n/a	n/a	II
ILS AS1-0075	2018/2/9	FPE	Study strain	1	8	8		n/a	n/a	n/a	II
ILS AS1-0076	2018/2/9	FPE	Study strain	1	20	20		n/a	n/a	n/a	II
ILS AS1-0077	2018/2/9	FPE	Study strain	1	29	29		n/a	n/a	n/a	II
ILS AS1-0078	2018/2/9	FPE	Study strain	2	9	9		n/a	n/a	n/a	II
ILS AS1-0079	2018/2/9	FPE	Study strain	1	121	121		n/a	n/a	n/a	II
ILS AS1-0080	2018/2/9	FPE	Study strain	1	121	121	3	absent	present	absent	II
ILS AS1-0081	2018/2/9	FPE	Study strain	2	9	9		n/a	n/a	n/a	II
ILS AS1-0082	2018/2/9	FPE	Study strain	2	9	9		n/a	n/a	n/a	II
ILS AS1-0083	2018/2/9	FPE	Study strain	1	204	204		n/a	n/a	n/a	II
ILS AS1-0084	2018/2/9	FPE	Study strain	1	204	204		n/a	n/a	n/a	II
ILS AS1-0085	2018/2/9	FPE	Study strain	2	9	9		n/a	n/a	n/a	II
ILS AS1-0086	2018/2/9	FPE	Study strain	1	20	20		n/a	n/a	n/a	II
ILS AS1-0087	2018/2/9	FPE	Study strain	1	204	204		n/a	n/a	n/a	II
ILS AS1-0088	2018/2/9	FPE	Study strain	2	9	9		n/a	n/a	n/a	II
ILS AS1-0089	2018/2/9	FPE	Study strain	2	9	9		n/a	n/a	n/a	II
ILS AS1-0090	2018/2/9	FPE	Study strain	2	9	9		n/a	n/a	n/a	II
ILS AS1-0091	2018/2/10	FPE	Study strain	2	9	9		n/a	n/a	n/a	II
ILS AS1-0092	2018/2/13	FPE	Study strain	2	9	9	1	present	absent	absent	II
ILS AS1-0093	2018/3/21	FPE	Study strain	2	9	9		n/a	n/a	n/a	II
ILS AS1-0094	2018/4/5	FPE	Study strain	2	9	9		n/a	n/a	n/a	II
ILS AS1-0095	2018/4/5	FPE	Study strain	2	9	9		n/a	n/a	n/a	II
ILS AS1-0096	2018/4/5	FPE	Study strain	2	9	9		n/a	n/a	n/a	II
ILS AS1-0097	2018/4/5	FPE	Study strain	2	9	9		n/a	n/a	n/a	II
ILS AS1-0098	2018/5/22	FPE	Study strain	1	204	204	2	present	absent	absent	II
ILS AS1-0099	2018/5/22	FPE	Study strain	1	204	204		n/a	n/a	n/a	II
ILS AS1-0100	2018/3/21	FPE	Study strain	2	9	9		n/a	n/a	n/a	II
ILS AS1-0101	2018/5/25	FPE	Study strain	2	9	9		n/a	n/a	n/a	II
ILS AS1-0102	2018/5/25	FPE	Study strain	2	9	9		n/a	n/a	n/a	II
ILS AS1-0103	2018/5/25	FPE	Study strain	2	9	9		n/a	n/a	n/a	II
ILS AS1-0104	2018/5/25	FPE	Study strain	2	9	9		present	absent	absent	II
ILS AS1-0105	2018/5/25	FPE	Study strain	2	9	9		n/a	n/a	n/a	II
ILS AS1-0106	2018/5/25	FPE	Study strain	2	9	9		n/a	n/a	n/a	II
ILS AS1-0107	2018/5/25	FPE	Study strain	2	9	9		n/a	n/a	n/a	II
ILS AS1-0108	2018/5/25	FPE	Study strain	4	6	6		n/a	n/a	n/a	I
ILS AS1-0109	2018/6/19	FP	Study strain	2	9	9		n/a	n/a	n/a	II
ILS AS1-0110	2018/6/25	FPE	Study strain	1	204	204		n/a	n/a	n/a	II
ILS AS1-0111	2018/6/25	FPE	Study strain	1	204	204	2	present	absent	absent	II
ILS AS1-0112	2018/6/26	FPE	Study strain	1	204	204		n/a	n/a	n/a	II
ILS AS1-0113	2018/6/26	FPE	Study strain	1	204	204		n/a	n/a	n/a	II
ILS AS1-0114	2018/6/26	FPE	Study strain	1	204	204		n/a	n/a	n/a	II
ILS AS1-0115	2018/6/26	FPE	Study strain	1	204	204	2	present	absent	absent	II
ILS AS1-0116	2018/6/26	FPE	Study strain	1	204	204		n/a	n/a	n/a	II
ILS AS1-0117	2018/6/26	FPE	Study strain	1	204	204		n/a	n/a	n/a	II
ILS AS1-0118	2018/6/26	FPE	Study strain	1	204	204		n/a	n/a	n/a	II
ILS AS1-0119	2028/6/25	FP	Study strain	2	9	9	1	present	absent	absent	II
ILS AS1-0120	2018/7/16	FPE	Study strain	1	204	204		n/a	n/a	n/a	II
ILS AS1-0121	2018/7/16	FPE	Study strain	1	204	204		n/a	n/a	n/a	II
ILS AS1-0122	2018/7/16	FPE	Study strain	1	204	204		n/a	n/a	n/a	II
ILS AS1-0123	2018/8/3	FP	Study strain	1	121	121		n/a	n/a	n/a	II
ILS AS1-0124	2018/8/9	FPE	Study strain	1	204	204		n/a	n/a	n/a	II
ILS-AS-R-001			PAA reference		204	204		n/a	n/a	n/a	II
ILS-AS-R-002			PAA reference		204	204		n/a	n/a	n/a	II
ILS-AS-R-003			PAA reference		204	204		n/a	n/a	n/a	II
ILS-AS-R-004			PAA reference		9	9		n/a	n/a	n/a	II
ILS-AS-R-005			PAA reference		9	9		n/a	n/a	n/a	II
ILS-AS-R-006			PAA reference		9	9		n/a	n/a	n/a	II
ILS-AS-R-007			PAA reference		9	9		n/a	n/a	n/a	II
ILS-AS-R-008			PAA reference		121	121		n/a	n/a	n/a	II
ILS-AS-R-009			PAA reference		6	6		n/a	n/a	n/a	I
N586			LBF	3a°	412	412		n/a	n/a	n/a	II
N11-1850			HBF	4b°	1290	217		n/a	n/a	n/a	I

* indicates strains that are associated with a cgMLST cluster without being properly in it. These strains were serotyped with antibodies from Denka-Seiken (Basel, Switzerland). n/a = not applicable; LBF = low biofilm former; HBF = high biofilm former; CC = clonal complex; WGS = core genome multi locus sequencing (cgMLST) cluster; FPE = food processing environment; FP = food product.

**Table 2:** Nucleotides used in this study.

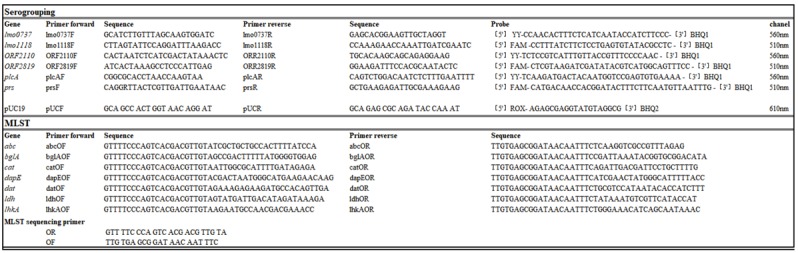

## Supplementary Materials

The following are available online at https://www.mdpi.com/2076-0817/8/1/32/s1.
