# Supplementary material for: Characteristics of Listeria Monocytogenes Strains Persisting in a Meat Processing Facility over a 4-Year Period
Source: Pathogens. 2019 Mar 7;8(1):32. doi: 10.3390/pathogens8010032 (PMC6471029; doi:10.3390/pathogens8010032)
Supplement: Supplementary file 1 [file pathogens-08-00032-s001.zip › pathogens-455627-supplementary.html]

Stoller et al. 2019 - stats file


Code 

- Show All Code
- Hide All Code

# Stoller et al. 2019 - stats file

#### *C. Guldimann*

#### *1/14/2019*

- Method
- Questions
- Biofilm
  - Setup and explore the data.
  - Visualize the data
  - Make a model for biofilm as a function of clonal complex
  - make a model for biofilm as a function of “strain”
- Benzalkonium chloride
  - Setup and explore the data
  - Visualize the Benzalkonium chloride data
  - Model the benzalkonium chloride data by clonal complex and by serotype
- Peracetic acid data
  - import and visualize the data for MIC
  - import and visualize the data for MBC
  - model the MBC data
- as a supplement, import the MBC / MIC data for all strains

# Method

Analyze strains isolated from a deli meat processor for their biofilm forming ability, and their resistance to the disinfectants benzalkonium chloride and peracetic acid.

# Questions

The majority of strains isolated from the facility belong to three groups of highly related clonal complexes (CC9, CC121, CC204), plus smaller clusters of strains that belong to other clonal complexes. The hypothesis is that the larger groups have persisted in the facility for at least 4 years, while strains that belong to isolated CC and are not repeatedly recovered may have been introduced sporadically. We aim to answer the following questions: 1. Among the clonal complexes isolated from the facility, are there strong biofilm formers compared to known high and low biofilm formers (HBF/LBF)? What is the influence of temperature on biofilm forming? 2. Among the clonal complexes isolated from the facility, are there groups that are resistant against benzalkonium chloride? 2. Among the clonal complexes isolated from the facility, are there groups that are resistant against peracetic acid? Since peracetic acid is routinely used in the facility, do we see an adaptation of strains to peracetic acid over time?

Load the required packages

```
#load required packages
library(readxl)
library(dplyr)
```

```
## 
## Attaching package: 'dplyr'
```

```
## The following objects are masked from 'package:stats':
## 
##     filter, lag
```

```
## The following objects are masked from 'package:base':
## 
##     intersect, setdiff, setequal, union
```

```
library(lme4)
```

```
## Loading required package: Matrix
```

```
library(ggplot2)
library(car)
```

```
## Loading required package: carData
```

```
## 
## Attaching package: 'car'
```

```
## The following object is masked from 'package:dplyr':
## 
##     recode
```

```
library(effects)
```

```
## lattice theme set by effectsTheme()
## See ?effectsTheme for details.
```

```
library(MASS)
```

```
## 
## Attaching package: 'MASS'
```

```
## The following object is masked from 'package:dplyr':
## 
##     select
```

```
library(lsmeans)
```

```
## The 'lsmeans' package is being deprecated.
## Users are encouraged to switch to 'emmeans'.
## See help('transition') for more information, including how
## to convert 'lsmeans' objects and scripts to work with 'emmeans'.
```

```
library(kableExtra)
library(multcompView)
```

# Biofilm

## Setup and explore the data.

The column “OD” is the OD of the culture at the time when Biofilm was measured. This data serves to verify that low biofilm formation is not due to inhibited growth of the strain.

```
#IMPORT DATA
biofilm_full <- read_excel("data/Biofilm-longlist_allreplicates.xlsx") 
biofilm_full%>%
  group_by(Stamm, CC, Temperatur, Zeit, Durchgang ) %>%
  summarize(Biofilm=mean(Biofilm), OD=mean(OD)) -> biofilm_1

biofilm_1 %>%
  kable("html") %>%
  kable_styling(bootstrap_options=c("striped",
                                    "hover",
                                    "condensed",
                                    "responsive"))
```

| Stamm | CC | Temperatur | Zeit | Durchgang | Biofilm | OD |
| --- | --- | --- | --- | --- | --- | --- |
| HBF | HBF CC412 | 22C | 96h | 1 | 0.2623333 | 0.0983333 |
| HBF | HBF CC412 | 22C | 96h | 2 | 0.3156667 | 0.1680000 |
| HBF | HBF CC412 | 22C | 96h | 3 | 0.2656667 | 0.0946667 |
| HBF | HBF CC412 | 8C | 168h | 1 | 0.1120000 | 0.3146667 |
| HBF | HBF CC412 | 8C | 168h | 2 | 0.1103333 | 0.3333333 |
| HBF | HBF CC412 | 8C | 168h | 3 | 0.1173333 | 0.0413333 |
| ILS AS-0015 | CC9 | 22C | 96h | 1 | 0.2803333 | 0.0976667 |
| ILS AS-0015 | CC9 | 22C | 96h | 2 | 0.4530000 | 0.3000000 |
| ILS AS-0015 | CC9 | 22C | 96h | 3 | 0.1600000 | 0.0866667 |
| ILS AS-0015 | CC9 | 8C | 168h | 1 | 0.1743333 | 0.2353333 |
| ILS AS-0015 | CC9 | 8C | 168h | 2 | 0.1046667 | 0.2966667 |
| ILS AS-0015 | CC9 | 8C | 168h | 3 | 0.1306667 | 0.2536667 |
| ILS AS-0016 | CC9 | 22C | 96h | 1 | 0.3513333 | 0.0520000 |
| ILS AS-0016 | CC9 | 22C | 96h | 2 | 0.3080000 | 0.2256667 |
| ILS AS-0016 | CC9 | 22C | 96h | 3 | 0.2553333 | 0.0583333 |
| ILS AS-0016 | CC9 | 8C | 168h | 1 | 0.1366667 | 0.3313333 |
| ILS AS-0016 | CC9 | 8C | 168h | 2 | 0.1183333 | 0.4430000 |
| ILS AS-0016 | CC9 | 8C | 168h | 3 | 0.1376667 | 0.2496667 |
| ILS AS-0018 | CC6 | 22C | 96h | 1 | 0.3050000 | 0.0696667 |
| ILS AS-0018 | CC6 | 22C | 96h | 2 | 0.2243333 | 0.2293333 |
| ILS AS-0018 | CC6 | 22C | 96h | 3 | 0.2206667 | 0.1290000 |
| ILS AS-0018 | CC6 | 8C | 168h | 1 | 0.1660000 | 0.2660000 |
| ILS AS-0018 | CC6 | 8C | 168h | 2 | 0.1106667 | 0.3156667 |
| ILS AS-0018 | CC6 | 8C | 168h | 3 | 0.1143333 | 0.2920000 |
| ILS AS-0021 | CC204 | 22C | 96h | 1 | 0.2686667 | 0.1083333 |
| ILS AS-0021 | CC204 | 22C | 96h | 2 | 0.3016667 | 0.2260000 |
| ILS AS-0021 | CC204 | 22C | 96h | 3 | 0.3650000 | 0.0820000 |
| ILS AS-0021 | CC204 | 8C | 168h | 1 | 0.1600000 | 0.2030000 |
| ILS AS-0021 | CC204 | 8C | 168h | 2 | 0.1143333 | 0.2553333 |
| ILS AS-0021 | CC204 | 8C | 168h | 3 | 0.0963333 | 0.0433333 |
| ILS AS-0054 | CC204 | 22C | 96h | 1 | 0.3933333 | 0.2453333 |
| ILS AS-0054 | CC204 | 22C | 96h | 2 | 0.2680000 | 0.1810000 |
| ILS AS-0054 | CC204 | 22C | 96h | 3 | 0.3333333 | 0.1286667 |
| ILS AS-0054 | CC204 | 8C | 168h | 1 | 0.1490000 | 0.3553333 |
| ILS AS-0054 | CC204 | 8C | 168h | 2 | 0.1353333 | 0.4483333 |
| ILS AS-0054 | CC204 | 8C | 168h | 3 | 0.1910000 | 0.2536667 |
| ILS AS-0079 | CC121 | 22C | 96h | 1 | 0.4870000 | 0.0680000 |
| ILS AS-0079 | CC121 | 22C | 96h | 2 | 0.2966667 | 0.1990000 |
| ILS AS-0079 | CC121 | 22C | 96h | 3 | 0.4066667 | 0.0670000 |
| ILS AS-0079 | CC121 | 8C | 168h | 1 | 0.1413333 | 0.2126667 |
| ILS AS-0079 | CC121 | 8C | 168h | 2 | 0.1226667 | 0.4033333 |
| ILS AS-0079 | CC121 | 8C | 168h | 3 | 0.1656667 | 0.2920000 |
| ILS AS-0080 | CC121 | 22C | 96h | 1 | 0.3490000 | 0.1256667 |
| ILS AS-0080 | CC121 | 22C | 96h | 2 | 0.1916667 | 0.1860000 |
| ILS AS-0080 | CC121 | 22C | 96h | 3 | 0.1830000 | 0.1160000 |
| ILS AS-0080 | CC121 | 8C | 168h | 1 | 0.1436667 | 0.2430000 |
| ILS AS-0080 | CC121 | 8C | 168h | 2 | 0.1323333 | 0.2926667 |
| ILS AS-0080 | CC121 | 8C | 168h | 3 | 0.1670000 | 0.2620000 |
| ILS AS-0101 | CC9 | 22C | 96h | 2 | 0.3720000 | 0.1543333 |
| ILS AS-0101 | CC9 | 22C | 96h | 3 | 0.4006667 | 0.0826667 |
| ILS AS-0101 | CC9 | 8C | 168h | 2 | 0.1490000 | 0.3853333 |
| ILS AS-0101 | CC9 | 8C | 168h | 3 | 0.2346667 | 0.2106667 |
| ILS AS-0105 | CC9 | 22C | 96h | 1 | 0.3376667 | 0.0853333 |
| ILS AS-0105 | CC9 | 22C | 96h | 2 | 0.3946667 | 0.1746667 |
| ILS AS-0105 | CC9 | 22C | 96h | 3 | 0.3936667 | 0.0723333 |
| ILS AS-0105 | CC9 | 8C | 168h | 1 | 0.1233333 | 0.3423333 |
| ILS AS-0105 | CC9 | 8C | 168h | 2 | 0.1276667 | 0.2856667 |
| ILS AS-0105 | CC9 | 8C | 168h | 3 | 0.1733333 | 0.2480000 |
| ILS AS-0108 | CC6 | 22C | 96h | 1 | 0.3646667 | 0.0946667 |
| ILS AS-0108 | CC6 | 22C | 96h | 2 | 0.3910000 | 0.4413333 |
| ILS AS-0108 | CC6 | 22C | 96h | 3 | 0.2153333 | 0.0876667 |
| ILS AS-0108 | CC6 | 8C | 168h | 1 | 0.1493333 | 0.3453333 |
| ILS AS-0108 | CC6 | 8C | 168h | 2 | 0.1343333 | 0.2910000 |
| ILS AS-0108 | CC6 | 8C | 168h | 3 | 0.1226667 | 0.2586667 |
| ILS AS1-0010 | CC9 | 22C | 96h | 1 | 0.3553333 | 0.1000000 |
| ILS AS1-0010 | CC9 | 22C | 96h | 2 | 0.3620000 | 0.1583333 |
| ILS AS1-0010 | CC9 | 22C | 96h | 3 | 0.3573333 | 0.0603333 |
| ILS AS1-0010 | CC9 | 8C | 168h | 1 | 0.1690000 | 0.3570000 |
| ILS AS1-0010 | CC9 | 8C | 168h | 2 | 0.1206667 | 0.2520000 |
| ILS AS1-0010 | CC9 | 8C | 168h | 3 | 0.1686667 | 0.2903333 |
| ILS AS1-0011 | CC9 | 22C | 96h | 1 | 0.2813333 | 0.1043333 |
| ILS AS1-0011 | CC9 | 22C | 96h | 2 | 0.4733333 | 0.2100000 |
| ILS AS1-0011 | CC9 | 22C | 96h | 3 | 0.3270000 | 0.0636667 |
| ILS AS1-0011 | CC9 | 8C | 168h | 1 | 0.1350000 | 0.2483333 |
| ILS AS1-0011 | CC9 | 8C | 168h | 2 | 0.1500000 | 0.2520000 |
| ILS AS1-0011 | CC9 | 8C | 168h | 3 | 0.2056667 | 0.3026667 |
| ILS AS1-0012 | CC9 | 22C | 96h | 1 | 0.2643333 | 0.0856667 |
| ILS AS1-0012 | CC9 | 22C | 96h | 2 | 0.3560000 | 0.1940000 |
| ILS AS1-0012 | CC9 | 22C | 96h | 3 | 0.3510000 | 0.0496667 |
| ILS AS1-0012 | CC9 | 8C | 168h | 1 | 0.1686667 | 0.3563333 |
| ILS AS1-0012 | CC9 | 8C | 168h | 2 | 0.1323333 | 0.2786667 |
| ILS AS1-0012 | CC9 | 8C | 168h | 3 | 0.1483333 | 0.3236667 |
| ILS AS1-0013 | CC9 | 22C | 96h | 1 | 0.2976667 | 0.1150000 |
| ILS AS1-0013 | CC9 | 22C | 96h | 2 | 0.2963333 | 0.2156667 |
| ILS AS1-0013 | CC9 | 22C | 96h | 3 | 0.4153333 | 0.0666667 |
| ILS AS1-0013 | CC9 | 8C | 168h | 1 | 0.1500000 | 0.2486667 |
| ILS AS1-0013 | CC9 | 8C | 168h | 2 | 0.1296667 | 0.3136667 |
| ILS AS1-0013 | CC9 | 8C | 168h | 3 | 0.1480000 | 0.2870000 |
| ILS AS1-0014 | CC9 | 22C | 96h | 1 | 0.3030000 | 0.0983333 |
| ILS AS1-0014 | CC9 | 22C | 96h | 2 | 0.3723333 | 0.1953333 |
| ILS AS1-0014 | CC9 | 22C | 96h | 3 | 0.3590000 | 0.0680000 |
| ILS AS1-0014 | CC9 | 8C | 168h | 1 | 0.1350000 | 0.2220000 |
| ILS AS1-0014 | CC9 | 8C | 168h | 2 | 0.1286667 | 0.4026667 |
| ILS AS1-0014 | CC9 | 8C | 168h | 3 | 0.1390000 | 0.2790000 |
| LBF | LBF CC217 | 22C | 96h | 1 | 0.1206667 | 0.0706667 |
| LBF | LBF CC217 | 22C | 96h | 2 | 0.1043333 | 0.1130000 |
| LBF | LBF CC217 | 22C | 96h | 3 | 0.1960000 | 0.0710000 |
| LBF | LBF CC217 | 8C | 168h | 1 | 0.1100000 | 0.0706667 |
| LBF | LBF CC217 | 8C | 168h | 2 | 0.0683333 | 0.1186667 |
| LBF | LBF CC217 | 8C | 168h | 3 | 0.1136667 | 0.2640000 |

```
#EXPLORE THE DATA
hist(biofilm_1$Biofilm)
```

```
hist(biofilm_1$OD)
```

```
plot(biofilm_1$Biofilm, as.factor(biofilm_1$CC))
```

```
plot(biofilm_1$Biofilm, biofilm_1$OD)  #es scheint dass die mit viel Biofilm wenig OD haben -> die Zellen sind im Biofilm
```

## Visualize the data

```
ggplot(biofilm_1, aes(x=CC, y=Biofilm)) + 
  geom_boxplot()+
  xlab("CC")+
  ylab("Biofilm")+
  theme(axis.title.x = element_text(),
        axis.text.x  = element_text(angle=90, vjust=0.5, size=12))+
  scale_y_continuous()+ 
  facet_grid(Temperatur~.)
```

```
  ggplot(biofilm_1, aes(x=Stamm, y=Biofilm)) + 
    geom_boxplot()+
    xlab("CC")+
    ylab("Biofilm")+
    theme(axis.title.x = element_text(),
          axis.text.x  = element_text(angle=90, vjust=0.5, size=12))+
    scale_y_continuous()+ 
    facet_grid(Temperatur~.)
```

```
ggplot(biofilm_1, aes(x=CC, y=OD)) + 
  geom_boxplot()+
  xlab("CC")+
  ylab("OD")+
  theme(axis.title.x = element_text(),
        axis.text.x  = element_text(angle=90, vjust=0.5, size=12))+
  scale_y_continuous()+ 
  facet_grid(Temperatur~.)
```

```
ggplot(biofilm_1, aes(x=Stamm, y=OD)) + 
  geom_boxplot()+
  xlab("Stamm")+
  ylab("OD")+
  theme(axis.title.x = element_text(),
        axis.text.x  = element_text(angle=90, vjust=0.5, size=12))+
  scale_y_continuous()
```

## Make a model for biofilm as a function of clonal complex

```
##BIOFILM AS A FUNCTION OF CC
#MAKE A MODEL: 
m_biofilm <- lmer(Biofilm ~ OD+Temperatur*CC + (1|Durchgang),
                  data=biofilm_1)

summary(m_biofilm)
```

```
## Linear mixed model fit by REML ['lmerMod']
## Formula: Biofilm ~ OD + Temperatur * CC + (1 | Durchgang)
##    Data: biofilm_1
## 
## REML criterion at convergence: -238.3
## 
## Scaled residuals: 
##     Min      1Q  Median      3Q     Max 
## -3.2808 -0.4844 -0.0225  0.4250  3.1608 
## 
## Random effects:
##  Groups    Name        Variance  Std.Dev. 
##  Durchgang (Intercept) 9.887e-20 3.144e-10
##  Residual              2.972e-03 5.452e-02
## Number of obs: 100, groups:  Durchgang, 3
## 
## Fixed effects:
##                            Estimate Std. Error t value
## (Intercept)               0.3096982  0.0241832  12.806
## OD                        0.0732746  0.0745073   0.983
## Temperatur8C             -0.1850841  0.0335879  -5.510
## CCCC204                   0.0001061  0.0315834   0.003
## CCCC6                    -0.0357083  0.0316813  -1.127
## CCCC9                     0.0228163  0.0246944   0.924
## CCHBF CC412              -0.0372934  0.0385531  -0.967
## CCLBF CC217              -0.1755851  0.0386771  -4.540
## Temperatur8C:CCCC204     -0.0027594  0.0447331  -0.062
## Temperatur8C:CCCC6        0.0223833  0.0446028   0.502
## Temperatur8C:CCCC9       -0.0214649  0.0349412  -0.614
## Temperatur8C:CCHBF CC412  0.0090646  0.0546345   0.166
## Temperatur8C:CCLBF CC217  0.1372317  0.0549389   2.498
```

```
## 
## Correlation matrix not shown by default, as p = 13 > 12.
## Use print(x, correlation=TRUE)  or
##   vcov(x)     if you need it
```

```
anova(m_biofilm)
```

```
## Analysis of Variance Table
##               Df  Sum Sq Mean Sq  F value
## OD             1 0.27114 0.27114  91.2252
## Temperatur     1 0.49838 0.49838 167.6805
## CC             5 0.08709 0.01742   5.8601
## Temperatur:CC  5 0.03469 0.00694   2.3341
```

```
#MODEL CHECKS
# Check normality of the residuals
hist(resid(m_biofilm))
```

```
qqnorm(resid(m_biofilm))
qqline(resid(m_biofilm))
```

```
plot(m_biofilm)
```

```
#DO CONTRASTS
lsm_biofilm <- lsmeans(m_biofilm,pairwise~CC|Temperatur,adjust="none")
```

```
## Loading required namespace: lmerTest
```

```
lsm_biofilm$contrasts %>%
  summary() %>%
  data.frame() %>%
  mutate(p.adj=p.adjust(p.value,method="holm")) -> lsm_df_biofilm_CC

lsm_biofilm <- lsmeans(m_biofilm,pairwise~Temperatur|CC,adjust="none")
lsm_biofilm$contrasts %>%
  summary() %>%
  data.frame() %>%
  mutate(p.adj=p.adjust(p.value,method="holm")) -> lsm_df_biofilm_Temp


#simple t-test to verify things
t.test(Biofilm ~ LBF, data = biofilm_full)
```

```
## 
##  Welch Two Sample t-test
## 
## data:  Biofilm by LBF
## t = 9.0488, df = 33.143, p-value = 1.787e-10
## alternative hypothesis: true difference in means is not equal to 0
## 95 percent confidence interval:
##  0.08915615 0.14086512
## sample estimates:
##  mean in group no mean in group yes 
##         0.2338440         0.1188333
```

```
t.test(Biofilm ~ LBF, data = subset(biofilm_full, biofilm_full$Temperatur== c("8C")))
```

```
## 
##  Welch Two Sample t-test
## 
## data:  Biofilm by LBF
## t = 3.8591, df = 9.182, p-value = 0.00371
## alternative hypothesis: true difference in means is not equal to 0
## 95 percent confidence interval:
##  0.01874544 0.07146733
## sample estimates:
##  mean in group no mean in group yes 
##        0.14243972        0.09733333
```

```
lsm_df_biofilm_CC %>%
  kable("html") %>%
  kable_styling(bootstrap_options=c("striped",
                                    "hover",
                                    "condensed",
                                    "responsive"))
```

| contrast | Temperatur | estimate | SE | df | t.ratio | p.value | p.adj |
| --- | --- | --- | --- | --- | --- | --- | --- |
| CC121 - CC204 | 22C | -0.0001061 | 0.0315834 | 87 | -0.0033602 | 0.9973267 | 1.0000000 |
| CC121 - CC6 | 22C | 0.0357083 | 0.0316813 | 87 | 1.1271104 | 0.2627949 | 1.0000000 |
| CC121 - CC9 | 22C | -0.0228163 | 0.0246944 | 87 | -0.9239482 | 0.3580688 | 1.0000000 |
| CC121 - HBF CC412 | 22C | 0.0372934 | 0.0385531 | 87 | 0.9673240 | 0.3360635 | 1.0000000 |
| CC121 - LBF CC217 | 22C | 0.1755851 | 0.0386771 | 87 | 4.5397674 | 0.0000180 | 0.0005214 |
| CC204 - CC6 | 22C | 0.0358144 | 0.0314917 | 87 | 1.1372639 | 0.2585505 | 1.0000000 |
| CC204 - CC9 | 22C | -0.0227102 | 0.0248690 | 87 | -0.9131913 | 0.3636657 | 1.0000000 |
| CC204 - HBF CC412 | 22C | 0.0373995 | 0.0386741 | 87 | 0.9670418 | 0.3362037 | 1.0000000 |
| CC204 - LBF CC217 | 22C | 0.1756912 | 0.0389745 | 87 | 4.5078464 | 0.0000203 | 0.0005688 |
| CC6 - CC9 | 22C | -0.0585246 | 0.0250076 | 87 | -2.3402744 | 0.0215579 | 0.5389469 |
| CC6 - HBF CC412 | 22C | 0.0015851 | 0.0387667 | 87 | 0.0408876 | 0.9674792 | 1.0000000 |
| CC6 - LBF CC217 | 22C | 0.1398768 | 0.0391338 | 87 | 3.5743205 | 0.0005757 | 0.0155438 |
| CC9 - HBF CC412 | 22C | 0.0601097 | 0.0332425 | 87 | 1.8082158 | 0.0740282 | 1.0000000 |
| CC9 - LBF CC217 | 22C | 0.1984014 | 0.0333577 | 87 | 5.9476928 | 0.0000001 | 0.0000017 |
| HBF CC412 - LBF CC217 | 22C | 0.1382917 | 0.0445919 | 87 | 3.1012717 | 0.0025975 | 0.0675343 |
| CC121 - CC204 | 8C | 0.0026533 | 0.0315286 | 87 | 0.0841550 | 0.9331265 | 1.0000000 |
| CC121 - CC6 | 8C | 0.0133249 | 0.0314856 | 87 | 0.4232069 | 0.6731887 | 1.0000000 |
| CC121 - CC9 | 8C | -0.0013514 | 0.0247071 | 87 | -0.0546967 | 0.9565055 | 1.0000000 |
| CC121 - HBF CC412 | 8C | 0.0282288 | 0.0387632 | 87 | 0.7282352 | 0.4684252 | 1.0000000 |
| CC121 - LBF CC217 | 8C | 0.0383534 | 0.0398063 | 87 | 0.9634994 | 0.3379674 | 1.0000000 |
| CC204 - CC6 | 8C | 0.0106717 | 0.0315834 | 87 | 0.3378878 | 0.7362617 | 1.0000000 |
| CC204 - CC9 | 8C | -0.0040047 | 0.0248382 | 87 | -0.1612307 | 0.8722855 | 1.0000000 |
| CC204 - HBF CC412 | 8C | 0.0255755 | 0.0386150 | 87 | 0.6623203 | 0.5095167 | 1.0000000 |
| CC204 - LBF CC217 | 8C | 0.0357001 | 0.0393919 | 87 | 0.9062805 | 0.3672906 | 1.0000000 |
| CC6 - CC9 | 8C | -0.0146763 | 0.0246919 | 87 | -0.5943786 | 0.5538015 | 1.0000000 |
| CC6 - HBF CC412 | 8C | 0.0149038 | 0.0388530 | 87 | 0.3835952 | 0.7022143 | 1.0000000 |
| CC6 - LBF CC217 | 8C | 0.0250284 | 0.0400085 | 87 | 0.6255780 | 0.5332294 | 1.0000000 |
| CC9 - HBF CC412 | 8C | 0.0295802 | 0.0336063 | 87 | 0.8801965 | 0.3811778 | 1.0000000 |
| CC9 - LBF CC217 | 8C | 0.0397048 | 0.0349509 | 87 | 1.1360155 | 0.2590698 | 1.0000000 |
| HBF CC412 - LBF CC217 | 8C | 0.0101246 | 0.0448979 | 87 | 0.2255032 | 0.8221167 | 1.0000000 |

```
lsm_df_biofilm_Temp %>%
  kable("html") %>%
  kable_styling(bootstrap_options=c("striped",
                                    "hover",
                                    "condensed",
                                    "responsive"))
```

| contrast | CC | estimate | SE | df | t.ratio | p.value | p.adj |
| --- | --- | --- | --- | --- | --- | --- | --- |
| 22C - 8C | CC121 | 0.1850841 | 0.0335879 | 87 | 5.510433 | 0.0000004 | 0.0000014 |
| 22C - 8C | CC204 | 0.1878435 | 0.0323108 | 87 | 5.813644 | 0.0000001 | 0.0000005 |
| 22C - 8C | CC6 | 0.1627008 | 0.0327110 | 87 | 4.973889 | 0.0000033 | 0.0000098 |
| 22C - 8C | CC9 | 0.2065490 | 0.0199118 | 87 | 10.373191 | 0.0000000 | 0.0000000 |
| 22C - 8C | HBF CC412 | 0.1760195 | 0.0452544 | 87 | 3.889555 | 0.0001960 | 0.0003919 |
| 22C - 8C | LBF CC217 | 0.0478524 | 0.0447863 | 87 | 1.068461 | 0.2882682 | 0.2882682 |

## make a model for biofilm as a function of “strain”

```
#-------------------------------------------------------------------
##BIOFILM AS A FUNCTION OF STRAIN
#MAKE A MODEL: 
m_biofilm_s <- lmer(Biofilm ~ OD+Temperatur*Stamm + (1|Durchgang),
                    data=biofilm_1)

summary(m_biofilm_s)
```

```
## Linear mixed model fit by REML ['lmerMod']
## Formula: Biofilm ~ OD + Temperatur * Stamm + (1 | Durchgang)
##    Data: biofilm_1
## 
## REML criterion at convergence: -161
## 
## Scaled residuals: 
##      Min       1Q   Median       3Q      Max 
## -2.48219 -0.40658 -0.08188  0.39509  2.72772 
## 
## Random effects:
##  Groups    Name        Variance  Std.Dev.
##  Durchgang (Intercept) 4.685e-05 0.006845
##  Residual              2.807e-03 0.052985
## Number of obs: 100, groups:  Durchgang, 3
## 
## Fixed effects:
##                                 Estimate Std. Error t value
## (Intercept)                     0.268195   0.032522   8.246
## OD                              0.108263   0.085670   1.264
## Temperatur8C                   -0.179849   0.044267  -4.063
## StammILS AS-0015                0.012105   0.043405   0.279
## StammILS AS-0016                0.024569   0.043268   0.568
## StammILS AS-0018               -0.033640   0.043305  -0.777
## StammILS AS-0021                0.028559   0.043291   0.660
## StammILS AS-0054                0.043332   0.043616   0.994
## StammILS AS-0079                0.116530   0.043269   2.693
## StammILS AS-0080               -0.042406   0.043304  -0.979
## StammILS AS-0101                0.106545   0.048423   2.200
## StammILS AS-0105                0.095146   0.043270   2.199
## StammILS AS-0108                0.032965   0.043908   0.751
## StammILS AS1-0010               0.078528   0.043279   1.814
## StammILS AS1-0011               0.078720   0.043265   1.819
## StammILS AS1-0012               0.043698   0.043272   1.010
## StammILS AS1-0013               0.053911   0.043275   1.246
## StammILS AS1-0014               0.063531   0.043262   1.469
## StammLBF                       -0.137052   0.043369  -3.160
## Temperatur8C:StammILS AS-0015   0.007752   0.061187   0.127
## Temperatur8C:StammILS AS-0016  -0.018979   0.062038  -0.306
## Temperatur8C:StammILS AS-0018   0.044099   0.061274   0.720
## Temperatur8C:StammILS AS-0021  -0.011453   0.061574  -0.186
## Temperatur8C:StammILS AS-0054  -0.011390   0.061384  -0.186
## Temperatur8C:StammILS AS-0079  -0.094421   0.061583  -1.533
## Temperatur8C:StammILS AS-0080   0.072941   0.061194   1.192
## Temperatur8C:StammILS AS-0101  -0.034084   0.068666  -0.496
## Temperatur8C:StammILS AS-0105  -0.073660   0.061490  -1.198
## Temperatur8C:StammILS AS-0108  -0.018165   0.061204  -0.297
## Temperatur8C:StammILS AS1-0010 -0.046551   0.061605  -0.756
## Temperatur8C:StammILS AS1-0011 -0.032488   0.061244  -0.530
## Temperatur8C:StammILS AS1-0012 -0.016862   0.061783  -0.273
## Temperatur8C:StammILS AS1-0013 -0.030352   0.061284  -0.495
## Temperatur8C:StammILS AS1-0014 -0.050266   0.061486  -0.818
## Temperatur8C:StammLBF           0.129679   0.061294   2.116
```

```
## 
## Correlation matrix not shown by default, as p = 35 > 12.
## Use print(x, correlation=TRUE)  or
##   vcov(x)     if you need it
```

```
anova(m_biofilm_s)
```

```
## Analysis of Variance Table
##                  Df  Sum Sq Mean Sq  F value
## OD                1 0.28308 0.28308 100.8327
## Temperatur        1 0.48876 0.48876 174.0935
## Stamm            16 0.13012 0.00813   2.8967
## Temperatur:Stamm 16 0.06509 0.00407   1.4491
```

```
#MODEL CHECKS
# Check normality of the residuals
hist(resid(m_biofilm_s))
```

```
qqnorm(resid(m_biofilm_s))
qqline(resid(m_biofilm_s))
```

```
plot(m_biofilm_s)
```

```
#DO CONTRASTS
lsm_biofilm_s <- lsmeans(m_biofilm_s,pairwise~Stamm|Temperatur,adjust="none")
lsm_biofilm_s$contrasts %>%
  summary() %>%
  data.frame() %>%
  group_by(Temperatur) %>%
  mutate(p.adj=p.adjust(p.value,method="holm")) -> lsm_df_biofilm_s

lsm_df_biofilm_s %>%
  kable("html") %>%
  kable_styling(bootstrap_options=c("striped",
                                    "hover",
                                    "condensed",
                                    "responsive"))
```

| contrast | Temperatur | estimate | SE | df | t.ratio | p.value | p.adj |
| --- | --- | --- | --- | --- | --- | --- | --- |
| HBF - ILS AS-0015 | 22C | -0.0121048 | 0.0434054 | 62.52544 | -0.2788766 | 0.7812598 | 1.0000000 |
| HBF - ILS AS-0016 | 22C | -0.0245689 | 0.0432682 | 62.11494 | -0.5678274 | 0.5721991 | 1.0000000 |
| HBF - ILS AS-0018 | 22C | 0.0336401 | 0.0433046 | 62.22686 | 0.7768253 | 0.4402049 | 1.0000000 |
| HBF - ILS AS-0021 | 22C | -0.0285587 | 0.0432911 | 62.18579 | -0.6596896 | 0.5118889 | 1.0000000 |
| HBF - ILS AS-0054 | 22C | -0.0433323 | 0.0436156 | 63.09291 | -0.9935066 | 0.3242582 | 1.0000000 |
| HBF - ILS AS-0079 | 22C | -0.1165299 | 0.0432692 | 62.11798 | -2.6931404 | 0.0090882 | 1.0000000 |
| HBF - ILS AS-0080 | 22C | 0.0424058 | 0.0433042 | 62.22559 | 0.9792556 | 0.3312457 | 1.0000000 |
| HBF - ILS AS-0101 | 22C | -0.1065445 | 0.0484231 | 62.93068 | -2.2002812 | 0.0314657 | 1.0000000 |
| HBF - ILS AS-0105 | 22C | -0.0951456 | 0.0432700 | 62.12070 | -2.1988803 | 0.0316188 | 1.0000000 |
| HBF - ILS AS-0108 | 22C | -0.0329654 | 0.0439077 | 63.75775 | -0.7507891 | 0.4555417 | 1.0000000 |
| HBF - ILS AS1-0010 | 22C | -0.0785277 | 0.0432792 | 62.14899 | -1.8144455 | 0.0744361 | 1.0000000 |
| HBF - ILS AS1-0011 | 22C | -0.0787198 | 0.0432650 | 62.10510 | -1.8194805 | 0.0736589 | 1.0000000 |
| HBF - ILS AS1-0012 | 22C | -0.0436983 | 0.0432717 | 62.12598 | -1.0098584 | 0.3164802 | 1.0000000 |
| HBF - ILS AS1-0013 | 22C | -0.0539110 | 0.0432747 | 62.13524 | -1.2457858 | 0.2175201 | 1.0000000 |
| HBF - ILS AS1-0014 | 22C | -0.0635315 | 0.0432623 | 62.09664 | -1.4685190 | 0.1470110 | 1.0000000 |
| HBF - LBF | 22C | 0.1370516 | 0.0433687 | 62.41876 | 3.1601479 | 0.0024323 | 0.2918814 |
| ILS AS-0015 - ILS AS-0016 | 22C | -0.0124641 | 0.0434692 | 62.70544 | -0.2867343 | 0.7752611 | 1.0000000 |
| ILS AS-0015 - ILS AS-0018 | 22C | 0.0457448 | 0.0432922 | 62.18901 | 1.0566536 | 0.2947572 | 1.0000000 |
| ILS AS-0015 - ILS AS-0021 | 22C | -0.0164540 | 0.0433058 | 62.23073 | -0.3799476 | 0.7052779 | 1.0000000 |
| ILS AS-0015 - ILS AS-0054 | 22C | -0.0312276 | 0.0433093 | 62.24132 | -0.7210361 | 0.4735870 | 1.0000000 |
| ILS AS-0015 - ILS AS-0079 | 22C | -0.1044252 | 0.0434748 | 62.72094 | -2.4019717 | 0.0192811 | 1.0000000 |
| ILS AS-0015 - ILS AS-0080 | 22C | 0.0545106 | 0.0432925 | 62.19010 | 1.2591220 | 0.2126919 | 1.0000000 |
| ILS AS-0015 - ILS AS-0101 | 22C | -0.0944397 | 0.0485816 | 63.52489 | -1.9439410 | 0.0563346 | 1.0000000 |
| ILS AS-0015 - ILS AS-0105 | 22C | -0.0830409 | 0.0434795 | 62.73396 | -1.9098857 | 0.0607224 | 1.0000000 |
| ILS AS-0015 - ILS AS-0108 | 22C | -0.0208607 | 0.0434449 | 62.63766 | -0.4801645 | 0.6327830 | 1.0000000 |
| ILS AS-0015 - ILS AS1-0010 | 22C | -0.0664230 | 0.0435202 | 62.84459 | -1.5262560 | 0.1319635 | 1.0000000 |
| ILS AS-0015 - ILS AS1-0011 | 22C | -0.0666151 | 0.0433687 | 62.41876 | -1.5360169 | 0.1295881 | 1.0000000 |
| ILS AS-0015 - ILS AS1-0012 | 22C | -0.0315936 | 0.0434881 | 62.75767 | -0.7264872 | 0.4702412 | 1.0000000 |
| ILS AS-0015 - ILS AS1-0013 | 22C | -0.0418063 | 0.0433336 | 62.31444 | -0.9647552 | 0.3383966 | 1.0000000 |
| ILS AS-0015 - ILS AS1-0014 | 22C | -0.0514267 | 0.0434039 | 62.52101 | -1.1848422 | 0.2405642 | 1.0000000 |
| ILS AS-0015 - LBF | 22C | 0.1491563 | 0.0437566 | 63.43192 | 3.4087739 | 0.0011387 | 0.1389212 |
| ILS AS-0016 - ILS AS-0018 | 22C | 0.0582089 | 0.0433420 | 62.33961 | 1.3430151 | 0.1841364 | 1.0000000 |
| ILS AS-0016 - ILS AS-0021 | 22C | -0.0039899 | 0.0433231 | 62.28289 | -0.0920954 | 0.9269178 | 1.0000000 |
| ILS AS-0016 - ILS AS-0054 | 22C | -0.0187635 | 0.0437120 | 63.32831 | -0.4292529 | 0.6691961 | 1.0000000 |
| ILS AS-0016 - ILS AS-0079 | 22C | -0.0919611 | 0.0432623 | 62.09674 | -2.1256616 | 0.0375159 | 1.0000000 |
| ILS AS-0016 - ILS AS-0080 | 22C | 0.0669747 | 0.0433414 | 62.33789 | 1.5452818 | 0.1273400 | 1.0000000 |
| ILS AS-0016 - ILS AS-0101 | 22C | -0.0819756 | 0.0484222 | 62.87366 | -1.6929358 | 0.0954176 | 1.0000000 |
| ILS AS-0016 - ILS AS-0105 | 22C | -0.0705768 | 0.0432624 | 62.09702 | -1.6313644 | 0.1078753 | 1.0000000 |
| ILS AS-0016 - ILS AS-0108 | 22C | -0.0083966 | 0.0440353 | 64.00264 | -0.1906786 | 0.8493810 | 1.0000000 |
| ILS AS-0016 - ILS AS1-0010 | 22C | -0.0539589 | 0.0432651 | 62.10544 | -1.2471675 | 0.2170189 | 1.0000000 |
| ILS AS-0016 - ILS AS1-0011 | 22C | -0.0541510 | 0.0432789 | 62.14817 | -1.2512096 | 0.2155476 | 1.0000000 |
| ILS AS-0016 - ILS AS1-0012 | 22C | -0.0191295 | 0.0432627 | 62.09793 | -0.4421700 | 0.6599024 | 1.0000000 |
| ILS AS-0016 - ILS AS1-0013 | 22C | -0.0293422 | 0.0432977 | 62.20597 | -0.6776842 | 0.5004849 | 1.0000000 |
| ILS AS-0016 - ILS AS1-0014 | 22C | -0.0389626 | 0.0432685 | 62.11593 | -0.9004852 | 0.3713401 | 1.0000000 |
| ILS AS-0016 - LBF | 22C | 0.1616204 | 0.0433246 | 62.28747 | 3.7304550 | 0.0004154 | 0.0519260 |
| ILS AS-0018 - ILS AS-0021 | 22C | -0.0621988 | 0.0432636 | 62.10062 | -1.4376714 | 0.1555474 | 1.0000000 |
| ILS AS-0018 - ILS AS-0054 | 22C | -0.0769724 | 0.0434140 | 62.55018 | -1.7729850 | 0.0810999 | 1.0000000 |
| ILS AS-0018 - ILS AS-0079 | 22C | -0.1501700 | 0.0433455 | 62.35004 | -3.4644901 | 0.0009665 | 0.1188759 |
| ILS AS-0018 - ILS AS-0080 | 22C | 0.0087657 | 0.0432623 | 62.09663 | 0.2026187 | 0.8400955 | 1.0000000 |
| ILS AS-0018 - ILS AS-0101 | 22C | -0.1401846 | 0.0484775 | 63.19645 | -2.8917430 | 0.0052475 | 0.6139551 |
| ILS AS-0018 - ILS AS-0105 | 22C | -0.1287857 | 0.0433485 | 62.35888 | -2.9709408 | 0.0042123 | 0.4970519 |
| ILS AS-0018 - ILS AS-0108 | 22C | -0.0666055 | 0.0436216 | 63.10819 | -1.5268924 | 0.1317845 | 1.0000000 |
| ILS AS-0018 - ILS AS1-0010 | 22C | -0.1121678 | 0.0433748 | 62.43660 | -2.5860127 | 0.0120550 | 1.0000000 |
| ILS AS-0018 - ILS AS1-0011 | 22C | -0.1123599 | 0.0432858 | 62.16954 | -2.5957663 | 0.0117615 | 1.0000000 |
| ILS AS-0018 - ILS AS1-0012 | 22C | -0.0773384 | 0.0433539 | 62.37515 | -1.7838843 | 0.0793068 | 1.0000000 |
| ILS AS-0018 - ILS AS1-0013 | 22C | -0.0875511 | 0.0432712 | 62.12416 | -2.0233141 | 0.0473487 | 1.0000000 |
| ILS AS-0018 - ILS AS1-0014 | 22C | -0.0971716 | 0.0433037 | 62.22431 | -2.2439537 | 0.0284026 | 1.0000000 |
| ILS AS-0018 - LBF | 22C | 0.1034115 | 0.0435445 | 62.90943 | 2.3748444 | 0.0206181 | 1.0000000 |
| ILS AS-0021 - ILS AS-0054 | 22C | -0.0147736 | 0.0434431 | 62.63277 | -0.3400684 | 0.7349429 | 1.0000000 |
| ILS AS-0021 - ILS AS-0079 | 22C | -0.0879712 | 0.0433261 | 62.29211 | -2.0304423 | 0.0465864 | 1.0000000 |
| ILS AS-0021 - ILS AS-0080 | 22C | 0.0709645 | 0.0432635 | 62.10039 | 1.6402868 | 0.1060017 | 1.0000000 |
| ILS AS-0021 - ILS AS-0101 | 22C | -0.0779858 | 0.0484626 | 63.13928 | -1.6091940 | 0.1125630 | 1.0000000 |
| ILS AS-0021 - ILS AS-0105 | 22C | -0.0665869 | 0.0433287 | 62.29995 | -1.5367839 | 0.1294099 | 1.0000000 |
| ILS AS-0021 - ILS AS-0108 | 22C | -0.0044067 | 0.0436656 | 63.21694 | -0.1009202 | 0.9199332 | 1.0000000 |
| ILS AS-0021 - ILS AS1-0010 | 22C | -0.0499690 | 0.0433521 | 62.36968 | -1.1526316 | 0.2534603 | 1.0000000 |
| ILS AS-0021 - ILS AS1-0011 | 22C | -0.0501611 | 0.0432761 | 62.13960 | -1.1590946 | 0.2508525 | 1.0000000 |
| ILS AS-0021 - ILS AS1-0012 | 22C | -0.0151396 | 0.0433336 | 62.31444 | -0.3493739 | 0.7279865 | 1.0000000 |
| ILS AS-0021 - ILS AS1-0013 | 22C | -0.0253523 | 0.0432657 | 62.10721 | -0.5859685 | 0.5600202 | 1.0000000 |
| ILS AS-0021 - ILS AS1-0014 | 22C | -0.0349728 | 0.0432904 | 62.18367 | -0.8078639 | 0.4222487 | 1.0000000 |
| ILS AS-0021 - LBF | 22C | 0.1656103 | 0.0435079 | 62.81153 | 3.8064401 | 0.0003224 | 0.0406189 |
| ILS AS-0054 - ILS AS-0079 | 22C | -0.0731976 | 0.0437202 | 63.34762 | -1.6742284 | 0.0990166 | 1.0000000 |
| ILS AS-0054 - ILS AS-0080 | 22C | 0.0857382 | 0.0434148 | 62.55246 | 1.9748593 | 0.0527018 | 1.0000000 |
| ILS AS-0054 - ILS AS-0101 | 22C | -0.0632122 | 0.0487869 | 64.01559 | -1.2956798 | 0.1997384 | 1.0000000 |
| ILS AS-0054 - ILS AS-0105 | 22C | -0.0518133 | 0.0437271 | 63.36376 | -1.1849242 | 0.2404727 | 1.0000000 |
| ILS AS-0054 - ILS AS-0108 | 22C | 0.0103669 | 0.0433067 | 62.23334 | 0.2393833 | 0.8115950 | 1.0000000 |
| ILS AS-0054 - ILS AS1-0010 | 22C | -0.0351954 | 0.0437855 | 63.49731 | -0.8038125 | 0.4245043 | 1.0000000 |
| ILS AS-0054 - ILS AS1-0011 | 22C | -0.0353875 | 0.0435566 | 62.94109 | -0.8124494 | 0.4195962 | 1.0000000 |
| ILS AS-0054 - ILS AS1-0012 | 22C | -0.0003660 | 0.0437396 | 63.39290 | -0.0083673 | 0.9933502 | 1.0000000 |
| ILS AS-0054 - ILS AS1-0013 | 22C | -0.0105787 | 0.0434959 | 62.77902 | -0.2432110 | 0.8086349 | 1.0000000 |
| ILS AS-0054 - ILS AS1-0014 | 22C | -0.0201992 | 0.0436131 | 63.08681 | -0.4631436 | 0.6448552 | 1.0000000 |
| ILS AS-0054 - LBF | 22C | 0.1803839 | 0.0441042 | 64.12346 | 4.0899473 | 0.0001227 | 0.0157070 |
| ILS AS-0079 - ILS AS-0080 | 22C | 0.1589358 | 0.0433449 | 62.34829 | 3.6667697 | 0.0005098 | 0.0632150 |
| ILS AS-0079 - ILS AS-0101 | 22C | 0.0099854 | 0.0484226 | 62.87016 | 0.2062142 | 0.8372899 | 1.0000000 |
| ILS AS-0079 - ILS AS-0105 | 22C | 0.0213843 | 0.0432623 | 62.09670 | 0.4942939 | 0.6228428 | 1.0000000 |
| ILS AS-0079 - ILS AS-0108 | 22C | 0.0835645 | 0.0440460 | 64.02191 | 1.8972093 | 0.0623124 | 1.0000000 |
| ILS AS-0079 - ILS AS1-0010 | 22C | 0.0380022 | 0.0432645 | 62.10352 | 0.8783693 | 0.3831291 | 1.0000000 |
| ILS AS-0079 - ILS AS1-0011 | 22C | 0.0378101 | 0.0432805 | 62.15317 | 0.8736047 | 0.3856965 | 1.0000000 |
| ILS AS-0079 - ILS AS1-0012 | 22C | 0.0728316 | 0.0432625 | 62.09726 | 1.6834811 | 0.0973033 | 1.0000000 |
| ILS AS-0079 - ILS AS1-0013 | 22C | 0.0626189 | 0.0433001 | 62.21314 | 1.4461609 | 0.1531514 | 1.0000000 |
| ILS AS-0079 - ILS AS1-0014 | 22C | 0.0529984 | 0.0432695 | 62.11905 | 1.2248448 | 0.2252582 | 1.0000000 |
| ILS AS-0079 - LBF | 22C | 0.2535815 | 0.0433216 | 62.27835 | 5.8534703 | 0.0000002 | 0.0000264 |
| ILS AS-0080 - ILS AS-0101 | 22C | -0.1489503 | 0.0484771 | 63.19476 | -3.0725928 | 0.0031296 | 0.3724281 |
| ILS AS-0080 - ILS AS-0105 | 22C | -0.1375515 | 0.0433479 | 62.35710 | -3.1732006 | 0.0023412 | 0.2832821 |
| ILS AS-0080 - ILS AS-0108 | 22C | -0.0753713 | 0.0436229 | 63.11126 | -1.7277936 | 0.0889160 | 1.0000000 |
| ILS AS-0080 - ILS AS1-0010 | 22C | -0.1209335 | 0.0433741 | 62.43460 | -2.7881498 | 0.0070166 | 0.8069087 |
| ILS AS-0080 - ILS AS1-0011 | 22C | -0.1211257 | 0.0432855 | 62.16857 | -2.7982949 | 0.0068318 | 0.7924846 |
| ILS AS-0080 - ILS AS1-0012 | 22C | -0.0861042 | 0.0433533 | 62.37332 | -1.9861029 | 0.0514208 | 1.0000000 |
| ILS AS-0080 - ILS AS1-0013 | 22C | -0.0963169 | 0.0432710 | 62.12357 | -2.2259011 | 0.0296580 | 1.0000000 |
| ILS AS-0080 - ILS AS1-0014 | 22C | -0.1059373 | 0.0433033 | 62.22305 | -2.4464019 | 0.0172683 | 1.0000000 |
| ILS AS-0080 - LBF | 22C | 0.0946457 | 0.0435435 | 62.90657 | 2.1735930 | 0.0335075 | 1.0000000 |
| ILS AS-0101 - ILS AS-0105 | 22C | 0.0113989 | 0.0484229 | 62.86736 | 0.2354025 | 0.8146615 | 1.0000000 |
| ILS AS-0101 - ILS AS-0108 | 22C | 0.0735791 | 0.0490651 | 64.49191 | 1.4996218 | 0.1385921 | 1.0000000 |
| ILS AS-0101 - ILS AS1-0010 | 22C | 0.0280168 | 0.0484277 | 62.84863 | 0.5785282 | 0.5649750 | 1.0000000 |
| ILS AS-0101 - ILS AS1-0011 | 22C | 0.0278247 | 0.0484298 | 62.98312 | 0.5745360 | 0.5676517 | 1.0000000 |
| ILS AS-0101 - ILS AS1-0012 | 22C | 0.0628462 | 0.0484237 | 62.86261 | 1.2978389 | 0.1990848 | 1.0000000 |
| ILS AS-0101 - ILS AS1-0013 | 22C | 0.0526335 | 0.0484433 | 63.05556 | 1.0864969 | 0.2813953 | 1.0000000 |
| ILS AS-0101 - ILS AS1-0014 | 22C | 0.0430130 | 0.0484233 | 62.93253 | 0.8882708 | 0.3777776 | 1.0000000 |
| ILS AS-0101 - LBF | 22C | 0.2435961 | 0.0484919 | 62.86155 | 5.0234412 | 0.0000045 | 0.0005989 |
| ILS AS-0105 - ILS AS-0108 | 22C | 0.0621802 | 0.0440550 | 64.03792 | 1.4114228 | 0.1629603 | 1.0000000 |
| ILS AS-0105 - ILS AS1-0010 | 22C | 0.0166179 | 0.0432640 | 62.10210 | 0.3841045 | 0.7022131 | 1.0000000 |
| ILS AS-0105 - ILS AS1-0011 | 22C | 0.0164258 | 0.0432819 | 62.15751 | 0.3795065 | 0.7056053 | 1.0000000 |
| ILS AS-0105 - ILS AS1-0012 | 22C | 0.0514473 | 0.0432624 | 62.09689 | 1.1891926 | 0.2388914 | 1.0000000 |
| ILS AS-0105 - ILS AS1-0013 | 22C | 0.0412346 | 0.0433021 | 62.21929 | 0.9522538 | 0.3446530 | 1.0000000 |
| ILS AS-0105 - ILS AS1-0014 | 22C | 0.0316141 | 0.0432704 | 62.12183 | 0.7306178 | 0.4677592 | 1.0000000 |
| ILS AS-0105 - LBF | 22C | 0.2321972 | 0.0433191 | 62.27092 | 5.3601572 | 0.0000013 | 0.0001738 |
| ILS AS-0108 - ILS AS1-0010 | 22C | -0.0455623 | 0.0441303 | 64.16714 | -1.0324479 | 0.3057381 | 1.0000000 |
| ILS AS-0108 - ILS AS1-0011 | 22C | -0.0457544 | 0.0438274 | 63.58941 | -1.0439678 | 0.3004529 | 1.0000000 |
| ILS AS-0108 - ILS AS1-0012 | 22C | -0.0107329 | 0.0440712 | 64.06660 | -0.2435350 | 0.8083691 | 1.0000000 |
| ILS AS-0108 - ILS AS1-0013 | 22C | -0.0209456 | 0.0437424 | 63.39939 | -0.4788393 | 0.6337003 | 1.0000000 |
| ILS AS-0108 - ILS AS1-0014 | 22C | -0.0305661 | 0.0439045 | 63.75116 | -0.6961944 | 0.4888376 | 1.0000000 |
| ILS AS-0108 - LBF | 22C | 0.1700170 | 0.0445271 | 64.69070 | 3.8182824 | 0.0003035 | 0.0385508 |
| ILS AS1-0010 - ILS AS1-0011 | 22C | -0.0001921 | 0.0432955 | 62.19902 | -0.0044378 | 0.9964733 | 1.0000000 |
| ILS AS1-0010 - ILS AS1-0012 | 22C | 0.0348294 | 0.0432634 | 62.09996 | 0.8050548 | 0.4238597 | 1.0000000 |
| ILS AS1-0010 - ILS AS1-0013 | 22C | 0.0246167 | 0.0433206 | 62.27536 | 0.5682442 | 0.5719125 | 1.0000000 |
| ILS AS1-0010 - ILS AS1-0014 | 22C | 0.0149962 | 0.0432797 | 62.15064 | 0.3464951 | 0.7301410 | 1.0000000 |
| ILS AS1-0010 - LBF | 22C | 0.2155793 | 0.0433009 | 62.21558 | 4.9786357 | 0.0000054 | 0.0007143 |
| ILS AS1-0011 - ILS AS1-0012 | 22C | 0.0350215 | 0.0432846 | 62.16572 | 0.8090987 | 0.4215443 | 1.0000000 |
| ILS AS1-0011 - ILS AS1-0013 | 22C | 0.0248088 | 0.0432658 | 62.10759 | 0.5734043 | 0.5684415 | 1.0000000 |
| ILS AS1-0011 - ILS AS1-0014 | 22C | 0.0151883 | 0.0432648 | 62.10445 | 0.3510555 | 0.7267348 | 1.0000000 |
| ILS AS1-0011 - LBF | 22C | 0.2157714 | 0.0434054 | 62.52544 | 4.9710717 | 0.0000055 | 0.0007228 |
| ILS AS1-0012 - ILS AS1-0013 | 22C | -0.0102127 | 0.0433058 | 62.23073 | -0.2358276 | 0.8143409 | 1.0000000 |
| ILS AS1-0012 - ILS AS1-0014 | 22C | -0.0198332 | 0.0432721 | 62.12723 | -0.4583357 | 0.6483105 | 1.0000000 |
| ILS AS1-0012 - LBF | 22C | 0.1807499 | 0.0433148 | 62.25791 | 4.1729363 | 0.0000950 | 0.0122521 |
| ILS AS1-0013 - ILS AS1-0014 | 22C | -0.0096205 | 0.0432743 | 62.13384 | -0.2223135 | 0.8247990 | 1.0000000 |
| ILS AS1-0013 - LBF | 22C | 0.1909626 | 0.0434537 | 62.66239 | 4.3946232 | 0.0000437 | 0.0056804 |
| ILS AS1-0014 - LBF | 22C | 0.2005831 | 0.0433701 | 62.42269 | 4.6249205 | 0.0000194 | 0.0025378 |
| HBF - ILS AS-0015 | 8C | -0.0198569 | 0.0433497 | 62.36246 | -0.4580635 | 0.6484990 | 1.0000000 |
| HBF - ILS AS-0016 | 8C | -0.0055894 | 0.0443053 | 64.43034 | -0.1261556 | 0.9000016 | 1.0000000 |
| HBF - ILS AS-0018 | 8C | -0.0104590 | 0.0435814 | 63.00562 | -0.2399873 | 0.8111192 | 1.0000000 |
| HBF - ILS AS-0021 | 8C | -0.0171058 | 0.0435930 | 63.03544 | -0.3923975 | 0.6960883 | 1.0000000 |
| HBF - ILS AS-0054 | 8C | -0.0319420 | 0.0445203 | 64.68397 | -0.7174697 | 0.4756674 | 1.0000000 |
| HBF - ILS AS-0079 | 8C | -0.0221089 | 0.0437106 | 63.32510 | -0.5058006 | 0.6147529 | 1.0000000 |
| HBF - ILS AS-0080 | 8C | -0.0305350 | 0.0433728 | 62.43060 | -0.7040124 | 0.4840428 | 1.0000000 |
| HBF - ILS AS-0101 | 8C | -0.0724601 | 0.0487418 | 63.21582 | -1.4866110 | 0.1420884 | 1.0000000 |
| HBF - ILS AS-0105 | 8C | -0.0214859 | 0.0435895 | 63.02646 | -0.4929146 | 0.6237859 | 1.0000000 |
| HBF - ILS AS-0108 | 8C | -0.0148002 | 0.0436591 | 63.20123 | -0.3389947 | 0.7357375 | 1.0000000 |
| HBF - ILS AS1-0010 | 8C | -0.0319772 | 0.0436759 | 63.24219 | -0.7321460 | 0.4667842 | 1.0000000 |
| HBF - ILS AS1-0011 | 8C | -0.0462314 | 0.0433839 | 62.46313 | -1.0656348 | 0.2906906 | 1.0000000 |
| HBF - ILS AS1-0012 | 8C | -0.0268360 | 0.0439407 | 63.82359 | -0.6107322 | 0.5435457 | 1.0000000 |
| HBF - ILS AS1-0013 | 8C | -0.0235593 | 0.0435029 | 62.79792 | -0.5415576 | 0.5900396 | 1.0000000 |
| HBF - ILS AS1-0014 | 8C | -0.0132652 | 0.0436931 | 63.28349 | -0.3036001 | 0.7624285 | 1.0000000 |
| HBF - LBF | 8C | 0.0073722 | 0.0437841 | 63.49403 | 0.1683769 | 0.8668220 | 1.0000000 |
| ILS AS-0015 - ILS AS-0016 | 8C | 0.0142675 | 0.0437944 | 63.51700 | 0.3257847 | 0.7456581 | 1.0000000 |
| ILS AS-0015 - ILS AS-0018 | 8C | 0.0093979 | 0.0433352 | 62.31937 | 0.2168658 | 0.8290213 | 1.0000000 |
| ILS AS-0015 - ILS AS-0021 | 8C | 0.0027511 | 0.0440159 | 63.96716 | 0.0625032 | 0.9503570 | 1.0000000 |
| ILS AS-0015 - ILS AS-0054 | 8C | -0.0120851 | 0.0439524 | 63.84657 | -0.2749591 | 0.7842355 | 1.0000000 |
| ILS AS-0015 - ILS AS-0079 | 8C | -0.0022520 | 0.0434031 | 62.51880 | -0.0518847 | 0.9587860 | 1.0000000 |
| ILS AS-0015 - ILS AS-0080 | 8C | -0.0106781 | 0.0432636 | 62.10085 | -0.2468137 | 0.8058665 | 1.0000000 |
| ILS AS-0015 - ILS AS-0101 | 8C | -0.0526032 | 0.0485039 | 62.87386 | -1.0845149 | 0.2822777 | 1.0000000 |
| ILS AS-0015 - ILS AS-0105 | 8C | -0.0016290 | 0.0433391 | 62.33108 | -0.0375868 | 0.9701373 | 1.0000000 |
| ILS AS-0015 - ILS AS-0108 | 8C | 0.0050567 | 0.0433748 | 62.43660 | 0.1165811 | 0.9075662 | 1.0000000 |
| ILS AS-0015 - ILS AS1-0010 | 8C | -0.0121203 | 0.0433839 | 62.46313 | -0.2793726 | 0.7808819 | 1.0000000 |
| ILS AS-0015 - ILS AS1-0011 | 8C | -0.0263745 | 0.0432651 | 62.10544 | -0.6096015 | 0.5443497 | 1.0000000 |
| ILS AS-0015 - ILS AS1-0012 | 8C | -0.0069791 | 0.0435435 | 62.90657 | -0.1602784 | 0.8731754 | 1.0000000 |
| ILS AS-0015 - ILS AS1-0013 | 8C | -0.0037024 | 0.0433005 | 62.21436 | -0.0855054 | 0.9321342 | 1.0000000 |
| ILS AS-0015 - ILS AS1-0014 | 8C | 0.0065917 | 0.0433933 | 62.49053 | 0.1519051 | 0.8797512 | 1.0000000 |
| ILS AS-0015 - LBF | 8C | 0.0272291 | 0.0442910 | 64.41072 | 0.6147779 | 0.5408672 | 1.0000000 |
| ILS AS-0016 - ILS AS-0018 | 8C | -0.0048696 | 0.0434748 | 62.72094 | -0.1120100 | 0.9111732 | 1.0000000 |
| ILS AS-0016 - ILS AS-0021 | 8C | -0.0115164 | 0.0457615 | 64.74463 | -0.2516614 | 0.8020999 | 1.0000000 |
| ILS AS-0016 - ILS AS-0054 | 8C | -0.0263526 | 0.0432728 | 62.12915 | -0.6089891 | 0.5447521 | 1.0000000 |
| ILS AS-0016 - ILS AS-0079 | 8C | -0.0165195 | 0.0433889 | 62.47777 | -0.3807306 | 0.7046947 | 1.0000000 |
| ILS AS-0016 - ILS AS-0080 | 8C | -0.0249456 | 0.0437424 | 63.39939 | -0.5702837 | 0.5705009 | 1.0000000 |
| ILS AS-0016 - ILS AS-0101 | 8C | -0.0668707 | 0.0485843 | 63.53246 | -1.3763855 | 0.1735342 | 1.0000000 |
| ILS AS-0016 - ILS AS-0105 | 8C | -0.0158965 | 0.0434682 | 62.70288 | -0.3657041 | 0.7158167 | 1.0000000 |
| ILS AS-0016 - ILS AS-0108 | 8C | -0.0092109 | 0.0434188 | 62.56393 | -0.2121395 | 0.8326878 | 1.0000000 |
| ILS AS-0016 - ILS AS1-0010 | 8C | -0.0263878 | 0.0434085 | 62.53437 | -0.6078946 | 0.5454588 | 1.0000000 |
| ILS AS-0016 - ILS AS1-0011 | 8C | -0.0406420 | 0.0437202 | 63.34762 | -0.9295939 | 0.3561103 | 1.0000000 |
| ILS AS-0016 - ILS AS1-0012 | 8C | -0.0212466 | 0.0433025 | 62.22054 | -0.4906555 | 0.6253961 | 1.0000000 |
| ILS AS-0016 - ILS AS1-0013 | 8C | -0.0179700 | 0.0435489 | 62.92090 | -0.4126389 | 0.6812739 | 1.0000000 |
| ILS AS-0016 - ILS AS1-0014 | 8C | -0.0076759 | 0.0433985 | 62.50565 | -0.1768693 | 0.8601829 | 1.0000000 |
| ILS AS-0016 - LBF | 8C | 0.0129616 | 0.0462298 | 64.22124 | 0.2803729 | 0.7800926 | 1.0000000 |
| ILS AS-0018 - ILS AS-0021 | 8C | -0.0066468 | 0.0445474 | 64.71054 | -0.1492071 | 0.8818543 | 1.0000000 |
| ILS AS-0018 - ILS AS-0054 | 8C | -0.0214830 | 0.0435791 | 62.99969 | -0.4929668 | 0.6237500 | 1.0000000 |
| ILS AS-0018 - ILS AS-0079 | 8C | -0.0116499 | 0.0432734 | 62.13112 | -0.2692158 | 0.7886556 | 1.0000000 |
| ILS AS-0018 - ILS AS-0080 | 8C | -0.0200760 | 0.0433167 | 62.26363 | -0.4634700 | 0.6446436 | 1.0000000 |
| ILS AS-0018 - ILS AS-0101 | 8C | -0.0620011 | 0.0484223 | 62.87218 | -1.2804242 | 0.2050997 | 1.0000000 |
| ILS AS-0018 - ILS AS-0105 | 8C | -0.0110269 | 0.0432623 | 62.09678 | -0.2548847 | 0.7996544 | 1.0000000 |
| ILS AS-0018 - ILS AS-0108 | 8C | -0.0043412 | 0.0432666 | 62.10997 | -0.1003371 | 0.9203999 | 1.0000000 |
| ILS AS-0018 - ILS AS1-0010 | 8C | -0.0215182 | 0.0432685 | 62.11593 | -0.4973179 | 0.6207208 | 1.0000000 |
| ILS AS-0018 - ILS AS1-0011 | 8C | -0.0357724 | 0.0433093 | 62.24132 | -0.8259747 | 0.4119734 | 1.0000000 |
| ILS AS-0018 - ILS AS1-0012 | 8C | -0.0163770 | 0.0433303 | 62.30473 | -0.3779570 | 0.7067472 | 1.0000000 |
| ILS AS-0018 - ILS AS1-0013 | 8C | -0.0131004 | 0.0432679 | 62.11398 | -0.3027732 | 0.7630743 | 1.0000000 |
| ILS AS-0018 - ILS AS1-0014 | 8C | -0.0028063 | 0.0432708 | 62.12298 | -0.0648535 | 0.9484989 | 1.0000000 |
| ILS AS-0018 - LBF | 8C | 0.0178312 | 0.0448966 | 64.94723 | 0.3971614 | 0.6925507 | 1.0000000 |
| ILS AS-0021 - ILS AS-0054 | 8C | -0.0148362 | 0.0460806 | 64.41624 | -0.3219630 | 0.7485232 | 1.0000000 |
| ILS AS-0021 - ILS AS-0079 | 8C | -0.0050031 | 0.0447914 | 64.89629 | -0.1116976 | 0.9114080 | 1.0000000 |
| ILS AS-0021 - ILS AS-0080 | 8C | -0.0134292 | 0.0440803 | 64.08247 | -0.3046528 | 0.7616178 | 1.0000000 |
| ILS AS-0021 - ILS AS-0101 | 8C | -0.0553543 | 0.0496417 | 64.44296 | -1.1150778 | 0.2689582 | 1.0000000 |
| ILS AS-0021 - ILS AS-0105 | 8C | -0.0043801 | 0.0445634 | 64.72560 | -0.0982895 | 0.9220062 | 1.0000000 |
| ILS AS-0021 - ILS AS-0108 | 8C | 0.0023056 | 0.0446966 | 64.83548 | 0.0515822 | 0.9590202 | 1.0000000 |
| ILS AS-0021 - ILS AS1-0010 | 8C | -0.0148714 | 0.0447279 | 64.85712 | -0.3324861 | 0.7405945 | 1.0000000 |
| ILS AS-0021 - ILS AS1-0011 | 8C | -0.0291256 | 0.0441098 | 64.13286 | -0.6602984 | 0.5114271 | 1.0000000 |
| ILS AS-0021 - ILS AS1-0012 | 8C | -0.0097302 | 0.0451878 | 64.99948 | -0.2153282 | 0.8301857 | 1.0000000 |
| ILS AS-0021 - ILS AS1-0013 | 8C | -0.0064536 | 0.0443869 | 64.53553 | -0.1453934 | 0.8848534 | 1.0000000 |
| ILS AS-0021 - ILS AS1-0014 | 8C | 0.0038405 | 0.0447595 | 64.87740 | 0.0858038 | 0.9318866 | 1.0000000 |
| ILS AS-0021 - LBF | 8C | 0.0244780 | 0.0432843 | 62.16479 | 0.5655166 | 0.5737580 | 1.0000000 |
| ILS AS-0054 - ILS AS-0079 | 8C | 0.0098331 | 0.0434720 | 62.71317 | 0.2261952 | 0.8217852 | 1.0000000 |
| ILS AS-0054 - ILS AS-0080 | 8C | 0.0014070 | 0.0438932 | 63.72806 | 0.0320560 | 0.9745276 | 1.0000000 |
| ILS AS-0054 - ILS AS-0101 | 8C | -0.0405181 | 0.0486715 | 63.75809 | -0.8324816 | 0.4082446 | 1.0000000 |
| ILS AS-0054 - ILS AS-0105 | 8C | 0.0104561 | 0.0435711 | 62.97903 | 0.2399784 | 0.8111264 | 1.0000000 |
| ILS AS-0054 - ILS AS-0108 | 8C | 0.0171418 | 0.0435099 | 62.81700 | 0.3939740 | 0.6949345 | 1.0000000 |
| ILS AS-0054 - ILS AS1-0010 | 8C | -0.0000352 | 0.0434969 | 62.78171 | -0.0008085 | 0.9993575 | 1.0000000 |
| ILS AS-0054 - ILS AS1-0011 | 8C | -0.0142894 | 0.0438677 | 63.67523 | -0.3257381 | 0.7456906 | 1.0000000 |
| ILS AS-0054 - ILS AS1-0012 | 8C | 0.0051060 | 0.0433539 | 62.37515 | 0.1177754 | 0.9066241 | 1.0000000 |
| ILS AS-0054 - ILS AS1-0013 | 8C | 0.0083827 | 0.0436681 | 63.22324 | 0.1919632 | 0.8483863 | 1.0000000 |
| ILS AS-0054 - ILS AS1-0014 | 8C | 0.0186768 | 0.0434843 | 62.74709 | 0.4295063 | 0.6690261 | 1.0000000 |
| ILS AS-0054 - LBF | 8C | 0.0393142 | 0.0465739 | 63.67997 | 0.8441261 | 0.4017589 | 1.0000000 |
| ILS AS-0079 - ILS AS-0080 | 8C | -0.0084261 | 0.0433769 | 62.44264 | -0.1942534 | 0.8466080 | 1.0000000 |
| ILS AS-0079 - ILS AS-0101 | 8C | -0.0503512 | 0.0484258 | 62.95555 | -1.0397597 | 0.3024284 | 1.0000000 |
| ILS AS-0079 - ILS AS-0105 | 8C | 0.0006230 | 0.0432719 | 62.12660 | 0.0143968 | 0.9885596 | 1.0000000 |
| ILS AS-0079 - ILS AS-0108 | 8C | 0.0073086 | 0.0432639 | 62.10158 | 0.1689317 | 0.8663994 | 1.0000000 |
| ILS AS-0079 - ILS AS1-0010 | 8C | -0.0098683 | 0.0432630 | 62.09883 | -0.2281006 | 0.8203175 | 1.0000000 |
| ILS AS-0079 - ILS AS1-0011 | 8C | -0.0241225 | 0.0433661 | 62.41097 | -0.5562534 | 0.5800266 | 1.0000000 |
| ILS AS-0079 - ILS AS1-0012 | 8C | -0.0047271 | 0.0432865 | 62.17148 | -0.1092055 | 0.9133913 | 1.0000000 |
| ILS AS-0079 - ILS AS1-0013 | 8C | -0.0014505 | 0.0432947 | 62.19676 | -0.0335022 | 0.9733814 | 1.0000000 |
| ILS AS-0079 - ILS AS1-0014 | 8C | 0.0088436 | 0.0432625 | 62.09717 | 0.2044179 | 0.8386955 | 1.0000000 |
| ILS AS-0079 - LBF | 8C | 0.0294811 | 0.0451686 | 64.99996 | 0.6526896 | 0.5162583 | 1.0000000 |
| ILS AS-0080 - ILS AS-0101 | 8C | -0.0419251 | 0.0484851 | 62.85524 | -0.8647013 | 0.3904909 | 1.0000000 |
| ILS AS-0080 - ILS AS-0105 | 8C | 0.0090491 | 0.0433201 | 62.27388 | 0.2088888 | 0.8352168 | 1.0000000 |
| ILS AS-0080 - ILS AS-0108 | 8C | 0.0157347 | 0.0433515 | 62.36787 | 0.3629575 | 0.7178639 | 1.0000000 |
| ILS AS-0080 - ILS AS1-0010 | 8C | -0.0014422 | 0.0433596 | 62.39187 | -0.0332616 | 0.9735722 | 1.0000000 |
| ILS AS-0080 - ILS AS1-0011 | 8C | -0.0156964 | 0.0432626 | 62.09746 | -0.3628177 | 0.7179731 | 1.0000000 |
| ILS AS-0080 - ILS AS1-0012 | 8C | 0.0036990 | 0.0435059 | 62.80607 | 0.0850226 | 0.9325139 | 1.0000000 |
| ILS AS-0080 - ILS AS1-0013 | 8C | 0.0069756 | 0.0432874 | 62.17444 | 0.1611469 | 0.8725002 | 1.0000000 |
| ILS AS-0080 - ILS AS1-0014 | 8C | 0.0172697 | 0.0433681 | 62.41680 | 0.3982130 | 0.6918320 | 1.0000000 |
| ILS AS-0080 - LBF | 8C | 0.0379072 | 0.0443657 | 64.50926 | 0.8544259 | 0.3960310 | 1.0000000 |
| ILS AS-0101 - ILS AS-0105 | 8C | 0.0509742 | 0.0484219 | 62.87639 | 1.0527091 | 0.2965032 | 1.0000000 |
| ILS AS-0101 - ILS AS-0108 | 8C | 0.0576599 | 0.0484222 | 62.91863 | 1.1907742 | 0.2382154 | 1.0000000 |
| ILS AS-0101 - ILS AS1-0010 | 8C | 0.0404829 | 0.0484231 | 62.93022 | 0.8360254 | 0.4063039 | 1.0000000 |
| ILS AS-0101 - ILS AS1-0011 | 8C | 0.0262287 | 0.0484775 | 62.84880 | 0.5410488 | 0.5903866 | 1.0000000 |
| ILS AS-0101 - ILS AS1-0012 | 8C | 0.0456241 | 0.0484673 | 63.15760 | 0.9413386 | 0.3501181 | 1.0000000 |
| ILS AS-0101 - ILS AS1-0013 | 8C | 0.0489008 | 0.0484319 | 62.84129 | 1.0096820 | 0.3165197 | 1.0000000 |
| ILS AS-0101 - ILS AS1-0014 | 8C | 0.0591949 | 0.0484243 | 62.94253 | 1.2224204 | 0.2261065 | 1.0000000 |
| ILS AS-0101 - LBF | 8C | 0.0798323 | 0.0499640 | 64.72438 | 1.5977975 | 0.1149591 | 1.0000000 |
| ILS AS-0105 - ILS AS-0108 | 8C | 0.0066857 | 0.0432657 | 62.10721 | 0.1545258 | 0.8776963 | 1.0000000 |
| ILS AS-0105 - ILS AS1-0010 | 8C | -0.0104913 | 0.0432674 | 62.11258 | -0.2424755 | 0.8092106 | 1.0000000 |
| ILS AS-0105 - ILS AS1-0011 | 8C | -0.0247455 | 0.0433125 | 62.25089 | -0.5713250 | 0.5698364 | 1.0000000 |
| ILS AS-0105 - ILS AS1-0012 | 8C | -0.0053501 | 0.0433266 | 62.29366 | -0.1234828 | 0.9021224 | 1.0000000 |
| ILS AS-0105 - ILS AS1-0013 | 8C | -0.0020734 | 0.0432690 | 62.11746 | -0.0479199 | 0.9619338 | 1.0000000 |
| ILS AS-0105 - ILS AS1-0014 | 8C | 0.0082206 | 0.0432695 | 62.11905 | 0.1899870 | 0.8499388 | 1.0000000 |
| ILS AS-0105 - LBF | 8C | 0.0288581 | 0.0449145 | 64.95416 | 0.6425125 | 0.5228020 | 1.0000000 |
| ILS AS-0108 - ILS AS1-0010 | 8C | -0.0171770 | 0.0432625 | 62.09717 | -0.3970406 | 0.6926989 | 1.0000000 |
| ILS AS-0108 - ILS AS1-0011 | 8C | -0.0314312 | 0.0433420 | 62.33961 | -0.7251898 | 0.4710491 | 1.0000000 |
| ILS AS-0108 - ILS AS1-0012 | 8C | -0.0120358 | 0.0433005 | 62.21436 | -0.2779590 | 0.7819655 | 1.0000000 |
| ILS AS-0108 - ILS AS1-0013 | 8C | -0.0087591 | 0.0432819 | 62.15751 | -0.2023733 | 0.8402858 | 1.0000000 |
| ILS AS-0108 - ILS AS1-0014 | 8C | 0.0015350 | 0.0432630 | 62.09883 | 0.0354802 | 0.9718106 | 1.0000000 |
| ILS AS-0108 - LBF | 8C | 0.0221724 | 0.0450633 | 64.99284 | 0.4920288 | 0.6243569 | 1.0000000 |
| ILS AS1-0010 - ILS AS1-0011 | 8C | -0.0142542 | 0.0433497 | 62.36246 | -0.3288195 | 0.7433938 | 1.0000000 |
| ILS AS1-0010 - ILS AS1-0012 | 8C | 0.0051412 | 0.0432955 | 62.19902 | 0.1187468 | 0.9058587 | 1.0000000 |
| ILS AS1-0010 - ILS AS1-0013 | 8C | 0.0084178 | 0.0432858 | 62.16954 | 0.1944711 | 0.8464411 | 1.0000000 |
| ILS AS1-0010 - ILS AS1-0014 | 8C | 0.0187119 | 0.0432625 | 62.09717 | 0.4325212 | 0.6668610 | 1.0000000 |
| ILS AS1-0010 - LBF | 8C | 0.0393494 | 0.0450981 | 64.99703 | 0.8725290 | 0.3861318 | 1.0000000 |
| ILS AS1-0011 - ILS AS1-0012 | 8C | 0.0193954 | 0.0434901 | 62.76299 | 0.4459732 | 0.6571513 | 1.0000000 |
| ILS AS1-0011 - ILS AS1-0013 | 8C | 0.0226721 | 0.0432825 | 62.15929 | 0.5238156 | 0.6022695 | 1.0000000 |
| ILS AS1-0011 - ILS AS1-0014 | 8C | 0.0329661 | 0.0433577 | 62.38625 | 0.7603298 | 0.4499215 | 1.0000000 |
| ILS AS1-0011 - LBF | 8C | 0.0536036 | 0.0443997 | 64.55105 | 1.2072971 | 0.2317230 | 1.0000000 |
| ILS AS1-0012 - ILS AS1-0013 | 8C | 0.0032766 | 0.0433748 | 62.43660 | 0.0755427 | 0.9400245 | 1.0000000 |
| ILS AS1-0012 - ILS AS1-0014 | 8C | 0.0135707 | 0.0432908 | 62.18473 | 0.3134787 | 0.7549661 | 1.0000000 |
| ILS AS1-0012 - LBF | 8C | 0.0342082 | 0.0456056 | 64.85840 | 0.7500881 | 0.4559141 | 1.0000000 |
| ILS AS1-0013 - ILS AS1-0014 | 8C | 0.0102941 | 0.0432901 | 62.18262 | 0.2377932 | 0.8128233 | 1.0000000 |
| ILS AS1-0013 - LBF | 8C | 0.0309315 | 0.0447158 | 64.84896 | 0.6917359 | 0.4915724 | 1.0000000 |
| ILS AS1-0014 - LBF | 8C | 0.0206375 | 0.0451332 | 64.99941 | 0.4572568 | 0.6490112 | 1.0000000 |

# Benzalkonium chloride

## Setup and explore the data

```
#BC data 
bc <- read_excel("data/171018BC_longlist_ohnejule.xlsx")
# Make readout an ordered factor
bc$readout <- factor(bc$readout, ordered=T)
bc$clonal.complex<-factor(bc$clonal.complex)
bc$serotype <- factor(bc$serotype)

bc %>%
  kable("html") %>%
  kable_styling(bootstrap_options=c("striped",
                                    "hover",
                                    "condensed",
                                    "responsive"))
```

| Stamm | serotype | clonal.complex | Zeit | Replikat | 0 | 2.5 | 5 | 7.5 | 10 | 15 | 20 | 25 | 30 | readout |
| --- | --- | --- | --- | --- | --- | --- | --- | --- | --- | --- | --- | --- | --- | --- |
| ILS AS1-0092 | 2 | CC9 | 48h | 1 | 4 | 4 | 4 | 4 | 4 | 0 | 0 | 0 | 0 | 10 |
| ILS AS1-0092 | 2 | CC9 | 48h | 2 | 4 | 4 | 4 | 4 | 4 | 0 | 0 | 0 | 0 | 10 |
| ILS AS1-0092 | 2 | CC9 | 48h | 3 | 4 | 4 | 4 | 4 | 4 | 0 | 0 | 0 | 0 | 10 |
| ILS AS1-0093 | 2 | CC9 | 48h | 1 | 4 | 4 | 4 | 4 | 4 | 0 | 0 | 0 | 0 | 10 |
| ILS AS1-0093 | 2 | CC9 | 48h | 2 | 4 | 4 | 4 | 4 | 4 | 0 | 0 | 0 | 0 | 10 |
| ILS AS1-0093 | 2 | CC9 | 48h | 3 | 4 | 4 | 4 | 4 | 4 | 0 | 0 | 0 | 0 | 10 |
| ILS AS1-0100 | 2 | CC9 | 48h | 1 | 4 | 4 | 4 | 4 | 4 | 0 | 0 | 0 | 0 | 10 |
| ILS AS1-0100 | 2 | CC9 | 48h | 2 | 4 | 4 | 4 | 4 | 4 | 0 | 0 | 0 | 0 | 10 |
| ILS AS1-0100 | 2 | CC9 | 48h | 3 | 4 | 4 | 4 | 4 | 4 | 0 | 0 | 0 | 0 | 10 |
| ILS AS1-0012 | 2 | CC9 | 48h | 1 | 4 | 4 | 4 | 4 | 4 | 0 | 0 | 0 | 0 | 10 |
| ILS AS1-0012 | 2 | CC9 | 48h | 2 | 4 | 4 | 4 | 4 | 4 | 0 | 0 | 0 | 0 | 10 |
| ILS AS1-0012 | 2 | CC9 | 48h | 3 | 4 | 4 | 4 | 4 | 4 | 0 | 0 | 0 | 0 | 10 |
| ILS AS1-0008 | 2 | CC9 | 48h | 1 | 4 | 4 | 4 | 4 | 3 | 0 | 0 | 0 | 0 | 10 |
| ILS AS1-0008 | 2 | CC9 | 48h | 2 | 4 | 4 | 4 | 4 | 0 | 0 | 0 | 0 | 0 | 7.5 |
| ILS AS1-0008 | 2 | CC9 | 48h | 3 | 4 | 4 | 4 | 3 | 2 | 0 | 0 | 0 | 0 | 7.5 |
| ILS AS1-0014 | 2 | CC9 | 48h | 1 | 4 | 4 | 4 | 4 | 4 | 0 | 0 | 0 | 0 | 10 |
| ILS AS1-0014 | 2 | CC9 | 48h | 2 | 4 | 4 | 4 | 4 | 4 | 0 | 0 | 0 | 0 | 10 |
| ILS AS1-0014 | 2 | CC9 | 48h | 3 | 4 | 4 | 4 | 4 | 4 | 0 | 0 | 0 | 0 | 10 |
| ILS AS1-0009 | 2 | CC9 | 48h | 1 | 4 | 4 | 4 | 4 | 4 | 0 | 0 | 0 | 0 | 10 |
| ILS AS1-0009 | 2 | CC9 | 48h | 2 | 4 | 4 | 4 | 4 | 0 | 0 | 0 | 0 | 0 | 7.5 |
| ILS AS1-0009 | 2 | CC9 | 48h | 3 | 4 | 4 | 4 | 4 | 3 | 0 | 0 | 0 | 0 | 10 |
| ILS AS1-0010 | 2 | CC9 | 48h | 1 | 4 | 4 | 4 | 4 | 4 | 0 | 0 | 0 | 0 | 10 |
| ILS AS1-0010 | 2 | CC9 | 48h | 2 | 4 | 4 | 4 | 4 | 4 | 0 | 0 | 0 | 0 | 10 |
| ILS AS1-0010 | 2 | CC9 | 48h | 3 | 4 | 4 | 4 | 4 | 4 | 0 | 0 | 0 | 0 | 10 |
| ILS AS1-0013 | 2 | CC9 | 48h | 1 | 4 | 4 | 4 | 4 | 4 | 0 | 0 | 0 | 0 | 10 |
| ILS AS1-0013 | 2 | CC9 | 48h | 2 | 4 | 4 | 4 | 4 | 4 | 0 | 0 | 0 | 0 | 10 |
| ILS AS1-0013 | 2 | CC9 | 48h | 3 | 4 | 4 | 4 | 4 | 4 | 0 | 0 | 0 | 0 | 10 |
| ILS AS1-0011 | 2 | CC9 | 48h | 1 | 4 | 4 | 4 | 4 | 4 | 0 | 0 | 0 | 0 | 10 |
| ILS AS1-0011 | 2 | CC9 | 48h | 2 | 4 | 4 | 4 | 4 | 4 | 0 | 0 | 0 | 0 | 10 |
| ILS AS1-0011 | 2 | CC9 | 48h | 3 | 4 | 4 | 4 | 4 | 4 | 0 | 0 | 0 | 0 | 10 |
| ILS AS1-0094 | 2 | CC9 | 48h | 1 | 4 | 4 | 4 | 4 | 4 | 0 | 0 | 0 | 0 | 10 |
| ILS AS1-0094 | 2 | CC9 | 48h | 2 | 4 | 4 | 4 | 4 | 4 | 0 | 0 | 0 | 0 | 10 |
| ILS AS1-0094 | 2 | CC9 | 48h | 3 | 4 | 4 | 4 | 4 | 4 | 0 | 0 | 0 | 0 | 10 |
| ILS AS1-0095 | 2 | CC9 | 48h | 1 | 4 | 4 | 4 | 4 | 4 | 0 | 0 | 0 | 0 | 10 |
| ILS AS1-0095 | 2 | CC9 | 48h | 2 | 4 | 4 | 4 | 4 | 4 | 0 | 0 | 0 | 0 | 10 |
| ILS AS1-0095 | 2 | CC9 | 48h | 3 | 4 | 4 | 4 | 4 | 4 | 0 | 0 | 0 | 0 | 10 |
| ILS AS1-0096 | 2 | CC9 | 48h | 1 | 4 | 4 | 4 | 4 | 4 | 0 | 0 | 0 | 0 | 10 |
| ILS AS1-0096 | 2 | CC9 | 48h | 2 | 4 | 4 | 4 | 4 | 4 | 0 | 0 | 0 | 0 | 10 |
| ILS AS1-0096 | 2 | CC9 | 48h | 3 | 4 | 4 | 4 | 4 | 4 | 0 | 0 | 0 | 0 | 10 |
| ILS AS1-0097 | 2 | CC9 | 48h | 1 | 4 | 4 | 4 | 4 | 4 | 0 | 0 | 0 | 0 | 10 |
| ILS AS1-0097 | 2 | CC9 | 48h | 2 | 4 | 4 | 4 | 4 | 4 | 0 | 0 | 0 | 0 | 10 |
| ILS AS1-0097 | 2 | CC9 | 48h | 3 | 4 | 4 | 4 | 4 | 4 | 0 | 0 | 0 | 0 | 10 |
| ILS AS1-0099 | 1 | CC204 | 48h | 1 | 4 | 4 | 4 | 4 | 4 | 0 | 0 | 0 | 0 | 10 |
| ILS AS1-0099 | 1 | CC204 | 48h | 2 | 4 | 4 | 4 | 4 | 4 | 0 | 0 | 0 | 0 | 10 |
| ILS AS1-0099 | 1 | CC204 | 48h | 3 | 4 | 4 | 4 | 4 | 4 | 0 | 0 | 0 | 0 | 10 |
| ILS AS1-0098 | 1 | CC204 | 48h | 1 | 4 | 4 | 4 | 4 | 4 | 0 | 0 | 0 | 0 | 10 |
| ILS AS1-0098 | 1 | CC204 | 48h | 2 | 4 | 4 | 4 | 4 | 4 | 0 | 0 | 0 | 0 | 10 |
| ILS AS1-0098 | 1 | CC204 | 48h | 3 | 4 | 4 | 4 | 4 | 4 | 0 | 0 | 0 | 0 | 10 |
| ILS AS1-0102 | 2 | CC9 | 48h | 1 | 4 | 4 | 4 | 4 | 4 | 0 | 0 | 0 | 0 | 10 |
| ILS AS1-0102 | 2 | CC9 | 48h | 2 | 4 | 4 | 4 | 4 | 4 | 0 | 0 | 0 | 0 | 10 |
| ILS AS1-0102 | 2 | CC9 | 48h | 3 | 4 | 4 | 4 | 4 | 4 | 0 | 0 | 0 | 0 | 10 |
| ILS AS1-0103 | 2 | CC9 | 48h | 1 | 4 | 4 | 4 | 4 | 4 | 0 | 0 | 0 | 0 | 10 |
| ILS AS1-0103 | 2 | CC9 | 48h | 2 | 4 | 4 | 4 | 4 | 4 | 0 | 0 | 0 | 0 | 10 |
| ILS AS1-0103 | 2 | CC9 | 48h | 3 | 4 | 4 | 4 | 4 | 4 | 0 | 0 | 0 | 0 | 10 |
| ILS AS1-0104 | 2 | CC9 | 48h | 1 | 4 | 4 | 4 | 4 | 4 | 0 | 0 | 0 | 0 | 10 |
| ILS AS1-0104 | 2 | CC9 | 48h | 2 | 4 | 4 | 4 | 4 | 4 | 0 | 0 | 0 | 0 | 10 |
| ILS AS1-0104 | 2 | CC9 | 48h | 3 | 4 | 4 | 4 | 4 | 4 | 0 | 0 | 0 | 0 | 10 |
| ILS AS1-0105 | 2 | CC9 | 48h | 1 | 4 | 4 | 4 | 4 | 4 | 0 | 0 | 0 | 0 | 10 |
| ILS AS1-0105 | 2 | CC9 | 48h | 2 | 4 | 4 | 4 | 4 | 4 | 0 | 0 | 0 | 0 | 10 |
| ILS AS1-0105 | 2 | CC9 | 48h | 3 | 4 | 4 | 4 | 4 | 4 | 0 | 0 | 0 | 0 | 10 |
| ILS AS1-0106 | 2 | CC9 | 48h | 1 | 4 | 4 | 4 | 4 | 4 | 0 | 0 | 0 | 0 | 10 |
| ILS AS1-0106 | 2 | CC9 | 48h | 2 | 4 | 4 | 4 | 4 | 4 | 0 | 0 | 0 | 0 | 10 |
| ILS AS1-0106 | 2 | CC9 | 48h | 3 | 4 | 4 | 4 | 4 | 4 | 0 | 0 | 0 | 0 | 10 |
| ILS AS1-0107 | 2 | CC9 | 48h | 1 | 4 | 4 | 4 | 4 | 4 | 0 | 0 | 0 | 0 | 10 |
| ILS AS1-0107 | 2 | CC9 | 48h | 2 | 4 | 4 | 4 | 4 | 4 | 0 | 0 | 0 | 0 | 10 |
| ILS AS1-0107 | 2 | CC9 | 48h | 3 | 4 | 4 | 4 | 4 | 4 | 0 | 0 | 0 | 0 | 10 |
| ILS AS1-0101 | 2 | CC9 | 48h | 1 | 4 | 4 | 4 | 4 | 4 | 0 | 0 | 0 | 0 | 10 |
| ILS AS1-0101 | 2 | CC9 | 48h | 2 | 4 | 4 | 4 | 4 | 4 | 0 | 0 | 0 | 0 | 10 |
| ILS AS1-0101 | 2 | CC9 | 48h | 3 | 4 | 4 | 4 | 4 | 4 | 0 | 0 | 0 | 0 | 10 |
| ILS AS1-0108 | 4 | CC6 | 48h | 1 | 4 | 4 | 4 | 2 | 1 | 0 | 0 | 0 | 0 | 5 |
| ILS AS1-0108 | 4 | CC6 | 48h | 2 | 4 | 4 | 4 | 1 | 1 | 0 | 0 | 0 | 0 | 5 |
| ILS AS1-0108 | 4 | CC6 | 48h | 3 | 4 | 4 | 4 | 2 | 2 | 0 | 0 | 0 | 0 | 5 |
| ILS AS1-0109 | 2 | CC9 | 48h | 1 | 4 | 4 | 4 | 4 | 4 | 0 | 0 | 0 | 0 | 10 |
| ILS AS1-0109 | 2 | CC9 | 48h | 2 | 4 | 4 | 4 | 4 | 4 | 0 | 0 | 0 | 0 | 10 |
| ILS AS1-0109 | 2 | CC9 | 48h | 3 | 4 | 4 | 4 | 4 | 4 | 0 | 0 | 0 | 0 | 10 |
| ILS AS1-0119 | 2 | CC9 | 48h | 1 | 4 | 4 | 4 | 4 | 4 | 0 | 0 | 0 | 0 | 10 |
| ILS AS1-0119 | 2 | CC9 | 48h | 2 | 4 | 4 | 4 | 4 | 4 | 0 | 0 | 0 | 0 | 10 |
| ILS AS1-0119 | 2 | CC9 | 48h | 3 | 4 | 4 | 4 | 4 | 4 | 0 | 0 | 0 | 0 | 10 |
| ILS AS1-0110 | 1 | CC204 | 48h | 1 | 4 | 4 | 4 | 4 | 4 | 0 | 0 | 0 | 0 | 10 |
| ILS AS1-0110 | 1 | CC204 | 48h | 2 | 4 | 4 | 4 | 4 | 4 | 0 | 0 | 0 | 0 | 10 |
| ILS AS1-0110 | 1 | CC204 | 48h | 3 | 4 | 4 | 4 | 4 | 4 | 0 | 0 | 0 | 0 | 10 |
| ILS AS1-0111 | 1 | CC204 | 48h | 1 | 4 | 4 | 4 | 4 | 4 | 0 | 0 | 0 | 0 | 10 |
| ILS AS1-0111 | 1 | CC204 | 48h | 2 | 4 | 4 | 4 | 4 | 4 | 0 | 0 | 0 | 0 | 10 |
| ILS AS1-0111 | 1 | CC204 | 48h | 3 | 4 | 4 | 4 | 4 | 4 | 0 | 0 | 0 | 0 | 10 |
| ILS AS1-0112 | 1 | CC204 | 48h | 1 | 4 | 4 | 4 | 4 | 4 | 0 | 0 | 0 | 0 | 10 |
| ILS AS1-0112 | 1 | CC204 | 48h | 2 | 4 | 4 | 4 | 4 | 4 | 0 | 0 | 0 | 0 | 10 |
| ILS AS1-0112 | 1 | CC204 | 48h | 3 | 4 | 4 | 4 | 4 | 4 | 0 | 0 | 0 | 0 | 10 |
| ILS AS1-0116 | 1 | CC204 | 48h | 1 | 4 | 4 | 4 | 4 | 4 | 0 | 0 | 0 | 0 | 10 |
| ILS AS1-0116 | 1 | CC204 | 48h | 2 | 4 | 4 | 4 | 4 | 4 | 0 | 0 | 0 | 0 | 10 |
| ILS AS1-0116 | 1 | CC204 | 48h | 3 | 4 | 4 | 4 | 4 | 4 | 0 | 0 | 0 | 0 | 10 |
| ILS AS1-0113 | 1 | CC204 | 48h | 1 | 4 | 4 | 4 | 4 | 4 | 0 | 0 | 0 | 0 | 10 |
| ILS AS1-0113 | 1 | CC204 | 48h | 2 | 4 | 4 | 4 | 4 | 4 | 0 | 0 | 0 | 0 | 10 |
| ILS AS1-0113 | 1 | CC204 | 48h | 3 | 4 | 4 | 4 | 4 | 4 | 0 | 0 | 0 | 0 | 10 |
| ILS AS1-0114 | 1 | CC204 | 48h | 1 | 4 | 4 | 4 | 4 | 4 | 0 | 0 | 0 | 0 | 10 |
| ILS AS1-0114 | 1 | CC204 | 48h | 2 | 4 | 4 | 4 | 4 | 4 | 0 | 0 | 0 | 0 | 10 |
| ILS AS1-0114 | 1 | CC204 | 48h | 3 | 4 | 4 | 4 | 4 | 4 | 0 | 0 | 0 | 0 | 10 |
| ILS AS1-0117 | 1 | CC204 | 48h | 1 | 4 | 4 | 4 | 4 | 4 | 0 | 0 | 0 | 0 | 10 |
| ILS AS1-0117 | 1 | CC204 | 48h | 2 | 4 | 4 | 4 | 4 | 4 | 0 | 0 | 0 | 0 | 10 |
| ILS AS1-0117 | 1 | CC204 | 48h | 3 | 4 | 4 | 4 | 4 | 4 | 0 | 0 | 0 | 0 | 10 |
| ILS AS1-0118 | 1 | CC204 | 48h | 1 | 4 | 4 | 4 | 4 | 4 | 0 | 0 | 0 | 0 | 10 |
| ILS AS1-0118 | 1 | CC204 | 48h | 2 | 4 | 4 | 4 | 4 | 4 | 0 | 0 | 0 | 0 | 10 |
| ILS AS1-0118 | 1 | CC204 | 48h | 3 | 4 | 4 | 4 | 4 | 4 | 0 | 0 | 0 | 0 | 10 |
| ILS AS1-0115 | 1 | CC204 | 48h | 1 | 4 | 4 | 4 | 4 | 4 | 0 | 0 | 0 | 0 | 10 |
| ILS AS1-0115 | 1 | CC204 | 48h | 2 | 4 | 4 | 4 | 4 | 4 | 0 | 0 | 0 | 0 | 10 |
| ILS AS1-0115 | 1 | CC204 | 48h | 3 | 4 | 4 | 4 | 4 | 4 | 0 | 0 | 0 | 0 | 10 |
| ILS AS1-0120 | 1 | CC204 | 48h | 1 | 4 | 4 | 4 | 4 | 4 | 0 | 0 | 0 | 0 | 10 |
| ILS AS1-0120 | 1 | CC204 | 48h | 2 | 4 | 4 | 4 | 4 | 4 | 0 | 0 | 0 | 0 | 10 |
| ILS AS1-0120 | 1 | CC204 | 48h | 3 | 4 | 4 | 4 | 4 | 4 | 0 | 0 | 0 | 0 | 10 |
| ILS AS1-0121 | 1 | CC204 | 48h | 1 | 4 | 4 | 4 | 4 | 4 | 0 | 0 | 0 | 0 | 10 |
| ILS AS1-0121 | 1 | CC204 | 48h | 2 | 4 | 4 | 4 | 4 | 4 | 0 | 0 | 0 | 0 | 10 |
| ILS AS1-0121 | 1 | CC204 | 48h | 3 | 4 | 4 | 4 | 4 | 4 | 0 | 0 | 0 | 0 | 10 |
| ILS AS1-0122 | 1 | CC204 | 48h | 1 | 4 | 4 | 4 | 4 | 4 | 0 | 0 | 0 | 0 | 10 |
| ILS AS1-0122 | 1 | CC204 | 48h | 2 | 4 | 4 | 4 | 4 | 4 | 0 | 0 | 0 | 0 | 10 |
| ILS AS1-0122 | 1 | CC204 | 48h | 3 | 4 | 4 | 4 | 4 | 4 | 0 | 0 | 0 | 0 | 10 |
| ILS AS1-0044 | 2 | CC9 | 48h | 1 | 4 | 4 | 4 | 4 | 4 | 0 | 0 | 0 | 0 | 10 |
| ILS AS1-0044 | 2 | CC9 | 48h | 2 | 4 | 4 | 3 | 2 | 2 | 0 | 0 | 0 | 0 | 5 |
| ILS AS1-0044 | 2 | CC9 | 48h | 3 | 4 | 4 | 3 | 2 | 1 | 0 | 0 | 0 | 0 | 5 |
| ILS AS1-0040 | 1 | CC204 | 48h | 1 | 4 | 4 | 3 | 0 | 0 | 0 | 0 | 0 | 0 | 5 |
| ILS AS1-0040 | 1 | CC204 | 48h | 2 | 4 | 4 | 4 | 4 | 4 | 0 | 0 | 0 | 0 | 10 |
| ILS AS1-0040 | 1 | CC204 | 48h | 3 | 4 | 4 | 4 | 4 | 4 | 0 | 0 | 0 | 0 | 10 |
| ILS AS1-0041 | 2 | CC9 | 48h | 1 | 4 | 4 | 4 | 4 | 4 | 0 | 0 | 0 | 0 | 10 |
| ILS AS1-0041 | 2 | CC9 | 48h | 2 | 4 | 4 | 4 | 4 | 4 | 0 | 0 | 0 | 0 | 10 |
| ILS AS1-0041 | 2 | CC9 | 48h | 3 | 4 | 4 | 4 | 4 | 4 | 0 | 0 | 0 | 0 | 10 |
| ILS AS1-0042 | 2 | CC9 | 48h | 1 | 4 | 4 | 4 | 4 | 4 | 0 | 0 | 0 | 0 | 10 |
| ILS AS1-0042 | 2 | CC9 | 48h | 2 | 4 | 4 | 4 | 4 | 4 | 0 | 0 | 0 | 0 | 10 |
| ILS AS1-0042 | 2 | CC9 | 48h | 3 | 4 | 4 | 4 | 4 | 4 | 0 | 0 | 0 | 0 | 10 |
| ILS AS1-0043 | 1 | CC29 | 48h | 1 | 4 | 4 | 4 | 4 | 4 | 0 | 0 | 0 | 0 | 10 |
| ILS AS1-0043 | 1 | CC29 | 48h | 2 | 4 | 4 | 4 | 4 | 4 | 0 | 0 | 0 | 0 | 10 |
| ILS AS1-0043 | 1 | CC29 | 48h | 3 | 4 | 4 | 4 | 4 | 4 | 0 | 0 | 0 | 0 | 10 |
| ILS AS1-0015 | 2 | CC9 | 48h | 1 | 4 | 4 | 4 | 4 | 4 | 0 | 0 | 0 | 0 | 10 |
| ILS AS1-0015 | 2 | CC9 | 48h | 2 | 4 | 4 | 4 | 4 | 4 | 0 | 0 | 0 | 0 | 10 |
| ILS AS1-0015 | 2 | CC9 | 48h | 3 | 4 | 4 | 4 | 4 | 4 | 0 | 0 | 0 | 0 | 10 |
| ILS AS1-0016 | 2 | CC9 | 48h | 1 | 4 | 4 | 4 | 4 | 4 | 0 | 0 | 0 | 0 | 10 |
| ILS AS1-0016 | 2 | CC9 | 48h | 2 | 4 | 4 | 4 | 4 | 4 | 0 | 0 | 0 | 0 | 10 |
| ILS AS1-0016 | 2 | CC9 | 48h | 3 | 4 | 4 | 4 | 4 | 4 | 0 | 0 | 0 | 0 | 10 |
| ILS AS1-0123 | 1 | CC121 | 48h | 1 | 4 | 4 | 4 | 4 | 3 | 0 | 0 | 0 | 0 | 10 |
| ILS AS1-0123 | 1 | CC121 | 48h | 2 | 4 | 4 | 4 | 4 | 4 | 0 | 0 | 0 | 0 | 10 |
| ILS AS1-0123 | 1 | CC204 | 48h | 3 | 4 | 4 | 4 | 4 | 4 | 0 | 0 | 0 | 0 | 10 |
| ILS AS1-0017 | 2 | CC9 | 48h | 1 | 4 | 4 | 4 | 4 | 4 | 0 | 0 | 0 | 0 | 10 |
| ILS AS1-0017 | 2 | CC9 | 48h | 2 | 4 | 4 | 4 | 4 | 4 | 0 | 0 | 0 | 0 | 10 |
| ILS AS1-0017 | 2 | CC9 | 48h | 3 | 4 | 4 | 4 | 4 | 4 | 0 | 0 | 0 | 0 | 10 |
| ILS AS1-0018 | 4 | CC6 | 48h | 1 | 4 | 4 | 1 | 0 | 0 | 0 | 0 | 0 | 0 | 2.5 |
| ILS AS1-0018 | 4 | CC6 | 48h | 2 | 4 | 4 | 2 | 1 | 0 | 0 | 0 | 0 | 0 | 2.5 |
| ILS AS1-0018 | 4 | CC6 | 48h | 3 | 4 | 4 | 1 | 1 | 0 | 0 | 0 | 0 | 0 | 2.5 |
| ILS AS1-0124 | 1 | CC204 | 48h | 1 | 4 | 4 | 4 | 4 | 4 | 0 | 0 | 0 | 0 | 10 |
| ILS AS1-0124 | 1 | CC204 | 48h | 2 | 4 | 4 | 4 | 4 | 4 | 0 | 0 | 0 | 0 | 10 |
| ILS AS1-0124 | 1 | CC204 | 48h | 3 | 4 | 4 | 4 | 4 | 4 | 0 | 0 | 0 | 0 | 10 |
| ILS AS1-0065 | 2 | CC9 | 48h | 1 | 4 | 4 | 4 | 4 | 4 | 0 | 0 | 0 | 0 | 10 |
| ILS AS1-0065 | 2 | CC9 | 48h | 2 | 4 | 4 | 4 | 4 | 4 | 0 | 0 | 0 | 0 | 10 |
| ILS AS1-0065 | 2 | CC9 | 48h | 3 | 4 | 4 | 4 | 4 | 4 | 0 | 0 | 0 | 0 | 10 |
| ILS AS1-0064 | 2 | CC9 | 48h | 1 | 4 | 4 | 4 | 4 | 4 | 0 | 0 | 0 | 0 | 10 |
| ILS AS1-0064 | 2 | CC9 | 48h | 2 | 4 | 4 | 4 | 4 | 2 | 0 | 0 | 0 | 0 | 7.5 |
| ILS AS1-0064 | 2 | CC9 | 48h | 3 | 4 | 4 | 4 | 4 | 4 | 0 | 0 | 0 | 0 | 10 |
| ILS AS1-0048 | 2 | CC9 | 48h | 1 | 4 | 4 | 4 | 4 | 4 | 0 | 0 | 0 | 0 | 10 |
| ILS AS1-0048 | 2 | CC9 | 48h | 2 | 4 | 4 | 4 | 4 | 4 | 0 | 0 | 0 | 0 | 10 |
| ILS AS1-0048 | 2 | CC9 | 48h | 3 | 4 | 4 | 4 | 4 | 4 | 0 | 0 | 0 | 0 | 10 |
| ILS AS1-0060 | 2 | CC9 | 48h | 1 | 4 | 4 | 4 | 4 | 4 | 0 | 0 | 0 | 0 | 10 |
| ILS AS1-0060 | 2 | CC9 | 48h | 2 | 4 | 4 | 4 | 4 | 4 | 0 | 0 | 0 | 0 | 10 |
| ILS AS1-0060 | 2 | CC9 | 48h | 3 | 4 | 4 | 4 | 4 | 4 | 0 | 0 | 0 | 0 | 10 |
| ILS AS1-0049 | 2 | CC9 | 48h | 1 | 4 | 4 | 4 | 4 | 4 | 0 | 0 | 0 | 0 | 10 |
| ILS AS1-0049 | 2 | CC9 | 48h | 2 | 4 | 4 | 4 | 4 | 4 | 0 | 0 | 0 | 0 | 10 |
| ILS AS1-0049 | 2 | CC9 | 48h | 3 | 4 | 4 | 4 | 4 | 4 | 0 | 0 | 0 | 0 | 10 |
| ILS AS1-0050 | 2 | CC9 | 48h | 1 | 4 | 4 | 4 | 4 | 4 | 0 | 0 | 0 | 0 | 10 |
| ILS AS1-0050 | 2 | CC9 | 48h | 2 | 4 | 4 | 4 | 4 | 4 | 0 | 0 | 0 | 0 | 10 |
| ILS AS1-0050 | 2 | CC9 | 48h | 3 | 4 | 4 | 4 | 4 | 4 | 0 | 0 | 0 | 0 | 10 |
| ILS AS1-0051 | 2 | CC9 | 48h | 1 | 4 | 4 | 4 | 4 | 4 | 0 | 0 | 0 | 0 | 10 |
| ILS AS1-0051 | 2 | CC9 | 48h | 2 | 4 | 4 | 4 | 4 | 4 | 0 | 0 | 0 | 0 | 10 |
| ILS AS1-0051 | 2 | CC9 | 48h | 3 | 4 | 4 | 4 | 4 | 4 | 0 | 0 | 0 | 0 | 10 |
| ILS AS1-0066 | 1 | CC89 | 48h | 1 | 4 | 4 | 2 | 0 | 0 | 0 | 0 | 0 | 0 | 2.5 |
| ILS AS1-0066 | 1 | CC89 | 48h | 2 | 4 | 4 | 1 | 0 | 0 | 0 | 0 | 0 | 0 | 2.5 |
| ILS AS1-0066 | 1 | CC89 | 48h | 3 | 4 | 4 | 2 | 1 | 0 | 0 | 0 | 0 | 0 | 2.5 |
| ILS AS1-0045 | 2 | CC9 | 48h | 1 | 4 | 4 | 4 | 4 | 4 | 0 | 0 | 0 | 0 | 10 |
| ILS AS1-0045 | 2 | CC9 | 48h | 2 | 4 | 4 | 4 | 4 | 4 | 0 | 0 | 0 | 0 | 10 |
| ILS AS1-0045 | 2 | CC9 | 48h | 3 | 4 | 4 | 4 | 4 | 4 | 0 | 0 | 0 | 0 | 10 |
| ILS AS1-0046 | 2 | CC9 | 48h | 1 | 4 | 4 | 4 | 4 | 4 | 0 | 0 | 0 | 0 | 10 |
| ILS AS1-0046 | 2 | CC9 | 48h | 2 | 4 | 4 | 4 | 4 | 4 | 0 | 0 | 0 | 0 | 10 |
| ILS AS1-0046 | 2 | CC9 | 48h | 3 | 4 | 4 | 4 | 4 | 4 | 0 | 0 | 0 | 0 | 10 |
| ILS AS1-0052 | 2 | CC9 | 48h | 1 | 4 | 4 | 4 | 4 | 4 | 0 | 0 | 0 | 0 | 10 |
| ILS AS1-0052 | 2 | CC9 | 48h | 2 | 4 | 4 | 4 | 4 | 4 | 0 | 0 | 0 | 0 | 10 |
| ILS AS1-0052 | 2 | CC9 | 48h | 3 | 4 | 4 | 4 | 4 | 4 | 0 | 0 | 0 | 0 | 10 |
| ILS AS1-0047 | 1 | CC204 | 48h | 1 | 4 | 4 | 4 | 4 | 4 | 0 | 0 | 0 | 0 | 10 |
| ILS AS1-0047 | 1 | CC204 | 48h | 2 | 4 | 4 | 4 | 4 | 4 | 0 | 0 | 0 | 0 | 10 |
| ILS AS1-0047 | 1 | CC204 | 48h | 3 | 4 | 4 | 4 | 4 | 4 | 0 | 0 | 0 | 0 | 10 |
| ILS AS1-0053 | 2 | CC9 | 48h | 1 | 4 | 4 | 4 | 4 | 4 | 0 | 0 | 0 | 0 | 10 |
| ILS AS1-0053 | 2 | CC9 | 48h | 2 | 4 | 4 | 4 | 4 | 4 | 0 | 0 | 0 | 0 | 10 |
| ILS AS1-0053 | 2 | CC9 | 48h | 3 | 4 | 4 | 4 | 4 | 4 | 0 | 0 | 0 | 0 | 10 |
| ILS AS1-0055 | 1 | CC204 | 48h | 1 | 4 | 4 | 4 | 4 | 4 | 0 | 0 | 0 | 0 | 10 |
| ILS AS1-0055 | 1 | CC204 | 48h | 2 | 4 | 4 | 4 | 4 | 4 | 0 | 0 | 0 | 0 | 10 |
| ILS AS1-0055 | 1 | CC204 | 48h | 3 | 4 | 4 | 4 | 4 | 4 | 0 | 0 | 0 | 0 | 10 |
| ILS AS1-0054 | 1 | CC204 | 48h | 1 | 4 | 4 | 4 | 4 | 4 | 0 | 0 | 0 | 0 | 10 |
| ILS AS1-0054 | 1 | CC204 | 48h | 2 | 4 | 4 | 4 | 4 | 2 | 0 | 0 | 0 | 0 | 7.5 |
| ILS AS1-0054 | 1 | CC204 | 48h | 3 | 4 | 4 | 4 | 4 | 4 | 0 | 0 | 0 | 0 | 10 |
| ILS AS1-0056 | 1 | CC204 | 48h | 1 | 4 | 4 | 4 | 4 | 4 | 0 | 0 | 0 | 0 | 10 |
| ILS AS1-0056 | 1 | CC204 | 48h | 2 | 4 | 4 | 4 | 4 | 2 | 0 | 0 | 0 | 0 | 7.5 |
| ILS AS1-0056 | 1 | CC204 | 48h | 3 | 4 | 4 | 4 | 4 | 4 | 0 | 0 | 0 | 0 | 10 |
| ILS AS1-0057 | 2 | CC9 | 48h | 1 | 4 | 4 | 4 | 4 | 4 | 0 | 0 | 0 | 0 | 10 |
| ILS AS1-0057 | 2 | CC9 | 48h | 2 | 4 | 4 | 4 | 4 | 4 | 0 | 0 | 0 | 0 | 10 |
| ILS AS1-0057 | 2 | CC9 | 48h | 3 | 4 | 4 | 4 | 4 | 4 | 0 | 0 | 0 | 0 | 10 |
| ILS AS1-0058 | 2 | CC9 | 48h | 1 | 4 | 4 | 4 | 4 | 4 | 0 | 0 | 0 | 0 | 10 |
| ILS AS1-0058 | 2 | CC9 | 48h | 2 | 4 | 4 | 4 | 4 | 4 | 0 | 0 | 0 | 0 | 10 |
| ILS AS1-0058 | 2 | CC9 | 48h | 3 | 4 | 4 | 4 | 4 | 4 | 0 | 0 | 0 | 0 | 10 |
| ILS AS1-0059 | 2 | CC9 | 48h | 1 | 4 | 4 | 4 | 4 | 4 | 0 | 0 | 0 | 0 | 10 |
| ILS AS1-0059 | 2 | CC9 | 48h | 2 | 4 | 4 | 4 | 4 | 4 | 0 | 0 | 0 | 0 | 10 |
| ILS AS1-0059 | 2 | CC9 | 48h | 3 | 4 | 4 | 4 | 4 | 4 | 0 | 0 | 0 | 0 | 10 |
| ILS AS1-0061 | 2 | CC9 | 48h | 1 | 4 | 4 | 4 | 4 | 4 | 0 | 0 | 0 | 0 | 10 |
| ILS AS1-0061 | 2 | CC9 | 48h | 2 | 4 | 4 | 4 | 4 | 4 | 0 | 0 | 0 | 0 | 10 |
| ILS AS1-0061 | 2 | CC9 | 48h | 3 | 4 | 4 | 4 | 4 | 4 | 0 | 0 | 0 | 0 | 10 |
| ILS AS1-0062 | 2 | CC9 | 48h | 1 | 4 | 4 | 4 | 4 | 4 | 0 | 0 | 0 | 0 | 10 |
| ILS AS1-0062 | 2 | CC9 | 48h | 2 | 4 | 4 | 4 | 4 | 4 | 0 | 0 | 0 | 0 | 10 |
| ILS AS1-0062 | 2 | CC9 | 48h | 3 | 4 | 4 | 4 | 4 | 4 | 0 | 0 | 0 | 0 | 10 |
| ILS AS1-0063 | 2 | CC9 | 48h | 1 | 4 | 4 | 4 | 4 | 4 | 0 | 0 | 0 | 0 | 10 |
| ILS AS1-0063 | 2 | CC9 | 48h | 2 | 4 | 4 | 4 | 4 | 4 | 0 | 0 | 0 | 0 | 10 |
| ILS AS1-0063 | 2 | CC9 | 48h | 3 | 4 | 4 | 4 | 4 | 4 | 0 | 0 | 0 | 0 | 10 |
| ILS AS1-0019 | 1 | CC121 | 48h | 1 | 4 | 4 | 4 | 4 | 3 | 0 | 0 | 0 | 0 | 10 |
| ILS AS1-0019 | 1 | CC121 | 48h | 2 | 4 | 4 | 4 | 3 | 0 | 0 | 0 | 0 | 0 | 7.5 |
| ILS AS1-0019 | 1 | CC121 | 48h | 3 | 4 | 4 | 4 | 3 | 3 | 0 | 0 | 0 | 0 | 10 |
| ILS AS1-0020 | 1 | CC204 | 48h | 1 | 4 | 4 | 4 | 4 | 4 | 0 | 0 | 0 | 0 | 10 |
| ILS AS1-0020 | 1 | CC204 | 48h | 2 | 4 | 4 | 4 | 4 | 4 | 0 | 0 | 0 | 0 | 10 |
| ILS AS1-0020 | 1 | CC204 | 48h | 3 | 4 | 4 | 4 | 4 | 4 | 0 | 0 | 0 | 0 | 10 |
| ILS AS1-0021 | 1 | CC204 | 48h | 1 | 4 | 4 | 4 | 4 | 4 | 0 | 0 | 0 | 0 | 10 |
| ILS AS1-0021 | 1 | CC204 | 48h | 2 | 4 | 4 | 4 | 4 | 4 | 0 | 0 | 0 | 0 | 10 |
| ILS AS1-0021 | 1 | CC204 | 48h | 3 | 4 | 4 | 4 | 4 | 4 | 0 | 0 | 0 | 0 | 10 |
| ILS AS1-0022 | 2 | CC9 | 48h | 1 | 4 | 4 | 4 | 4 | 4 | 0 | 0 | 0 | 0 | 10 |
| ILS AS1-0022 | 2 | CC9 | 48h | 2 | 4 | 4 | 4 | 4 | 4 | 0 | 0 | 0 | 0 | 10 |
| ILS AS1-0022 | 2 | CC9 | 48h | 3 | 4 | 4 | 4 | 4 | 4 | 0 | 0 | 0 | 0 | 10 |
| ILS AS1-0023 | 1 | CC204 | 48h | 1 | 4 | 4 | 4 | 4 | 4 | 0 | 0 | 0 | 0 | 10 |
| ILS AS1-0023 | 1 | CC204 | 48h | 2 | 4 | 4 | 4 | 4 | 4 | 0 | 0 | 0 | 0 | 10 |
| ILS AS1-0023 | 1 | CC204 | 48h | 3 | 4 | 4 | 4 | 4 | 4 | 0 | 0 | 0 | 0 | 10 |
| ILS AS1-0005 | 2 | CC9 | 48h | 1 | 4 | 4 | 4 | 4 | 3 | 0 | 0 | 0 | 0 | 10 |
| ILS AS1-0005 | 2 | CC9 | 48h | 2 | 4 | 4 | 4 | 4 | 0 | 0 | 0 | 0 | 0 | 7.5 |
| ILS AS1-0005 | 2 | CC9 | 48h | 3 | 4 | 4 | 4 | 3 | 3 | 0 | 0 | 0 | 0 | 10 |
| ILS AS1-0001 | 2 | CC9 | 48h | 1 | 4 | 4 | 4 | 2 | 0 | 0 | 0 | 0 | 0 | 7.5 |
| ILS AS1-0001 | 2 | CC9 | 48h | 2 | 4 | 4 | 4 | 3 | 0 | 0 | 0 | 0 | 0 | 7.5 |
| ILS AS1-0001 | 2 | CC9 | 48h | 3 | 4 | 4 | 4 | 3 | 3 | 0 | 0 | 0 | 0 | 10 |
| ILS AS1-0002 | 1 | CC121 | 48h | 1 | 4 | 4 | 4 | 4 | 3 | 0 | 0 | 0 | 0 | 10 |
| ILS AS1-0002 | 1 | CC121 | 48h | 2 | 4 | 4 | 4 | 4 | 0 | 0 | 0 | 0 | 0 | 7.5 |
| ILS AS1-0002 | 1 | CC121 | 48h | 3 | 4 | 4 | 4 | 4 | 4 | 0 | 0 | 0 | 0 | 10 |
| ILS AS1-0007 | 1 | CC204 | 48h | 1 | 4 | 4 | 4 | 4 | 4 | 0 | 0 | 0 | 0 | 10 |
| ILS AS1-0007 | 1 | CC204 | 48h | 2 | 4 | 4 | 4 | 4 | 4 | 0 | 0 | 0 | 0 | 10 |
| ILS AS1-0007 | 1 | CC204 | 48h | 3 | 4 | 4 | 4 | 4 | 4 | 0 | 0 | 0 | 0 | 10 |
| ILS AS1-0024 | 2 | CC9 | 48h | 1 | 4 | 4 | 4 | 4 | 4 | 0 | 0 | 0 | 0 | 10 |
| ILS AS1-0024 | 2 | CC9 | 48h | 2 | 4 | 4 | 4 | 4 | 4 | 0 | 0 | 0 | 0 | 10 |
| ILS AS1-0024 | 2 | CC9 | 48h | 3 | 4 | 4 | 4 | 4 | 4 | 0 | 0 | 0 | 0 | 10 |
| ILS AS1-0025 | 2 | CC9 | 48h | 1 | 4 | 4 | 4 | 4 | 4 | 0 | 0 | 0 | 0 | 10 |
| ILS AS1-0025 | 2 | CC9 | 48h | 2 | 4 | 4 | 4 | 4 | 4 | 0 | 0 | 0 | 0 | 10 |
| ILS AS1-0025 | 2 | CC9 | 48h | 3 | 4 | 4 | 4 | 4 | 4 | 0 | 0 | 0 | 0 | 10 |
| ILS AS1-0026 | 1 | CC204 | 48h | 1 | 4 | 4 | 3 | 3 | 0 | 0 | 0 | 0 | 0 | 7.5 |
| ILS AS1-0026 | 1 | CC204 | 48h | 2 | 4 | 4 | 4 | 4 | 0 | 0 | 0 | 0 | 0 | 7.5 |
| ILS AS1-0026 | 1 | CC204 | 48h | 3 | 4 | 4 | 4 | 3 | 3 | 0 | 0 | 0 | 0 | 10 |
| ILS AS1-0003 | 1 | CC204 | 48h | 1 | 4 | 4 | 4 | 3 | 2 | 0 | 0 | 0 | 0 | 7.5 |
| ILS AS1-0003 | 1 | CC204 | 48h | 2 | 4 | 4 | 4 | 4 | 0 | 0 | 0 | 0 | 0 | 7.5 |
| ILS AS1-0003 | 1 | CC204 | 48h | 3 | 4 | 4 | 4 | 4 | 4 | 0 | 0 | 0 | 0 | 10 |
| ILS AS1-0067 | 2 | CC9 | 48h | 1 | 4 | 4 | 4 | 4 | 4 | 0 | 0 | 0 | 0 | 10 |
| ILS AS1-0067 | 2 | CC9 | 48h | 2 | 4 | 4 | 4 | 4 | 4 | 0 | 0 | 0 | 0 | 10 |
| ILS AS1-0067 | 2 | CC9 | 48h | 3 | 4 | 4 | 4 | 4 | 4 | 0 | 0 | 0 | 0 | 10 |
| ILS AS1-0004 | 1 | CC204 | 48h | 1 | 4 | 4 | 4 | 3 | 2 | 0 | 0 | 0 | 0 | 7.5 |
| ILS AS1-0004 | 1 | CC204 | 48h | 2 | 4 | 4 | 4 | 3 | 0 | 0 | 0 | 0 | 0 | 7.5 |
| ILS AS1-0004 | 1 | CC204 | 48h | 3 | 4 | 4 | 4 | 4 | 4 | 0 | 0 | 0 | 0 | 10 |
| ILS AS1-0068 | 2 | CC9 | 48h | 1 | 4 | 4 | 4 | 4 | 4 | 0 | 0 | 0 | 0 | 10 |
| ILS AS1-0068 | 2 | CC9 | 48h | 2 | 4 | 4 | 4 | 4 | 4 | 0 | 0 | 0 | 0 | 10 |
| ILS AS1-0068 | 2 | CC9 | 48h | 3 | 4 | 4 | 4 | 4 | 4 | 0 | 0 | 0 | 0 | 10 |
| ILS AS1-0070 | 2 | CC9 | 48h | 1 | 4 | 4 | 4 | 4 | 4 | 0 | 0 | 0 | 0 | 10 |
| ILS AS1-0070 | 2 | CC9 | 48h | 2 | 4 | 4 | 4 | 4 | 4 | 0 | 0 | 0 | 0 | 10 |
| ILS AS1-0070 | 2 | CC9 | 48h | 3 | 4 | 4 | 4 | 4 | 4 | 0 | 0 | 0 | 0 | 10 |
| ILS AS1-0027 | 2 | CC9 | 48h | 1 | 4 | 4 | 4 | 4 | 3 | 0 | 0 | 0 | 0 | 10 |
| ILS AS1-0027 | 2 | CC9 | 48h | 2 | 4 | 4 | 4 | 4 | 0 | 0 | 0 | 0 | 0 | 7.5 |
| ILS AS1-0027 | 2 | CC9 | 48h | 3 | 4 | 4 | 4 | 4 | 4 | 0 | 0 | 0 | 0 | 10 |
| ILS AS1-0035 | 2 | CC9 | 48h | 1 | 4 | 4 | 4 | 4 | 4 | 0 | 0 | 0 | 0 | 10 |
| ILS AS1-0035 | 2 | CC9 | 48h | 2 | 4 | 4 | 4 | 4 | 4 | 0 | 0 | 0 | 0 | 10 |
| ILS AS1-0035 | 2 | CC9 | 48h | 3 | 4 | 4 | 4 | 4 | 4 | 0 | 0 | 0 | 0 | 10 |
| ILS AS1-0036 | 2 | CC9 | 48h | 1 | 4 | 4 | 4 | 4 | 4 | 0 | 0 | 0 | 0 | 10 |
| ILS AS1-0036 | 2 | CC9 | 48h | 2 | 4 | 4 | 4 | 4 | 4 | 0 | 0 | 0 | 0 | 10 |
| ILS AS1-0036 | 2 | CC9 | 48h | 3 | 4 | 4 | 4 | 4 | 4 | 0 | 0 | 0 | 0 | 10 |
| ILS AS1-0037 | 2 | CC9 | 48h | 1 | 4 | 4 | 4 | 4 | 4 | 0 | 0 | 0 | 0 | 10 |
| ILS AS1-0037 | 2 | CC9 | 48h | 2 | 4 | 4 | 4 | 4 | 4 | 0 | 0 | 0 | 0 | 10 |
| ILS AS1-0037 | 2 | CC9 | 48h | 3 | 4 | 4 | 4 | 4 | 4 | 0 | 0 | 0 | 0 | 10 |
| ILS AS1-0038 | 2 | CC9 | 48h | 1 | 4 | 4 | 4 | 4 | 4 | 0 | 0 | 0 | 0 | 10 |
| ILS AS1-0038 | 2 | CC9 | 48h | 2 | 4 | 4 | 4 | 4 | 4 | 0 | 0 | 0 | 0 | 10 |
| ILS AS1-0038 | 2 | CC9 | 48h | 3 | 4 | 4 | 4 | 4 | 4 | 0 | 0 | 0 | 0 | 10 |
| ILS AS1-0028 | 2 | CC9 | 48h | 1 | 4 | 4 | 4 | 4 | 4 | 0 | 0 | 0 | 0 | 10 |
| ILS AS1-0028 | 2 | CC9 | 48h | 2 | 4 | 4 | 4 | 4 | 4 | 0 | 0 | 0 | 0 | 10 |
| ILS AS1-0028 | 2 | CC9 | 48h | 3 | 4 | 4 | 4 | 4 | 4 | 0 | 0 | 0 | 0 | 10 |
| ILS AS1-0029 | 2 | CC9 | 48h | 1 | 4 | 4 | 4 | 4 | 4 | 0 | 0 | 0 | 0 | 10 |
| ILS AS1-0029 | 2 | CC9 | 48h | 2 | 4 | 4 | 4 | 4 | 4 | 0 | 0 | 0 | 0 | 10 |
| ILS AS1-0029 | 2 | CC9 | 48h | 3 | 4 | 4 | 4 | 4 | 4 | 0 | 0 | 0 | 0 | 10 |
| ILS AS1-0030 | 2 | CC9 | 48h | 1 | 4 | 4 | 4 | 4 | 4 | 0 | 0 | 0 | 0 | 10 |
| ILS AS1-0030 | 2 | CC9 | 48h | 2 | 4 | 4 | 4 | 4 | 4 | 0 | 0 | 0 | 0 | 10 |
| ILS AS1-0030 | 2 | CC9 | 48h | 3 | 4 | 4 | 4 | 4 | 4 | 0 | 0 | 0 | 0 | 10 |
| ILS AS1-0031 | 1 | CC204 | 48h | 1 | 4 | 4 | 4 | 4 | 4 | 0 | 0 | 0 | 0 | 10 |
| ILS AS1-0031 | 1 | CC204 | 48h | 2 | 4 | 4 | 4 | 4 | 4 | 0 | 0 | 0 | 0 | 10 |
| ILS AS1-0031 | 1 | CC204 | 48h | 3 | 4 | 4 | 4 | 4 | 4 | 0 | 0 | 0 | 0 | 10 |
| ILS AS1-0069 | 1 | CC29 | 48h | 1 | 4 | 4 | 2 | 0 | 0 | 0 | 0 | 0 | 0 | 2.5 |
| ILS AS1-0069 | 1 | CC29 | 48h | 2 | 4 | 4 | 1 | 0 | 0 | 0 | 0 | 0 | 0 | 2.5 |
| ILS AS1-0069 | 1 | CC29 | 48h | 3 | 4 | 4 | 2 | 1 | 0 | 0 | 0 | 0 | 0 | 2.5 |
| ILS AS1-0032 | 1 | CC20 | 48h | 1 | 4 | 4 | 4 | 4 | 4 | 0 | 0 | 0 | 0 | 10 |
| ILS AS1-0032 | 1 | CC20 | 48h | 1 | 4 | 4 | 3 | 0 | 0 | 0 | 0 | 0 | 0 | 5 |
| ILS AS1-0033 | 1 | CC20 | 48h | 2 | 4 | 4 | 4 | 4 | 4 | 0 | 0 | 0 | 0 | 10 |
| ILS AS1-0032 | 1 | CC20 | 48h | 2 | 4 | 4 | 4 | 2 | 0 | 0 | 0 | 0 | 0 | 5 |
| ILS AS1-0033 | 1 | CC20 | 48h | 3 | 4 | 4 | 4 | 4 | 4 | 0 | 0 | 0 | 0 | 10 |
| ILS AS1-0033 | 1 | CC20 | 48h | 3 | 4 | 4 | 4 | 1 | 0 | 0 | 0 | 0 | 0 | 7.5 |
| ILS AS1-0034 | 2 | CC9 | 48h | 1 | 4 | 4 | 4 | 3 | 0 | 0 | 0 | 0 | 0 | 7.5 |
| ILS AS1-0034 | 2 | CC9 | 48h | 2 | 4 | 4 | 4 | 4 | 0 | 0 | 0 | 0 | 0 | 7.5 |
| ILS AS1-0034 | 2 | CC9 | 48h | 3 | 4 | 4 | 4 | 3 | 3 | 0 | 0 | 0 | 0 | 10 |
| ILS AS1-0039 | 1 | CC121 | 48h | 1 | 4 | 4 | 4 | 4 | 4 | 0 | 0 | 0 | 0 | 10 |
| ILS AS1-0039 | 1 | CC121 | 48h | 2 | 4 | 4 | 4 | 4 | 4 | 0 | 0 | 0 | 0 | 10 |
| ILS AS1-0039 | 1 | CC121 | 48h | 3 | 4 | 4 | 4 | 4 | 4 | 0 | 0 | 0 | 0 | 10 |
| ILS AS1-0071 | 2 | CC9 | 48h | 1 | 4 | 4 | 4 | 4 | 4 | 0 | 0 | 0 | 0 | 10 |
| ILS AS1-0071 | 2 | CC9 | 48h | 2 | 4 | 4 | 4 | 4 | 4 | 0 | 0 | 0 | 0 | 10 |
| ILS AS1-0071 | 2 | CC9 | 48h | 3 | 4 | 4 | 4 | 4 | 4 | 0 | 0 | 0 | 0 | 10 |
| ILS AS1-0073 | 4 | CC6 | 48h | 1 | 4 | 4 | 4 | 3 | 2 | 0 | 0 | 0 | 0 | 7.5 |
| ILS AS1-0073 | 4 | CC6 | 48h | 2 | 4 | 4 | 4 | 2 | 1 | 0 | 0 | 0 | 0 | 5 |
| ILS AS1-0073 | 4 | CC6 | 48h | 3 | 4 | 4 | 4 | 3 | 2 | 0 | 0 | 0 | 0 | 7.5 |
| ILS AS1-0072 | 2 | CC9 | 48h | 1 | 4 | 4 | 4 | 4 | 1 | 0 | 0 | 0 | 0 | 7.5 |
| ILS AS1-0072 | 2 | CC9 | 48h | 2 | 4 | 4 | 4 | 4 | 4 | 0 | 0 | 0 | 0 | 10 |
| ILS AS1-0072 | 2 | CC9 | 48h | 3 | 4 | 4 | 4 | 4 | 4 | 0 | 0 | 0 | 0 | 10 |
| ILS AS1-0074 | 1 | CC29 | 48h | 1 | 4 | 4 | 4 | 2 | 1 | 0 | 0 | 0 | 0 | 5 |
| ILS AS1-0074 | 1 | CC29 | 48h | 2 | 4 | 4 | 2 | 2 | 1 | 0 | 0 | 0 | 0 | 2.5 |
| ILS AS1-0074 | 1 | CC29 | 48h | 3 | 4 | 4 | 2 | 0 | 0 | 0 | 0 | 0 | 0 | 2.5 |
| ILS AS1-0075 | 1 | CC8 | 48h | 1 | 4 | 4 | 4 | 3 | 2 | 0 | 0 | 0 | 0 | 7.5 |
| ILS AS1-0075 | 1 | CC8 | 48h | 2 | 4 | 4 | 2 | 2 | 1 | 0 | 0 | 0 | 0 | 2.5 |
| ILS AS1-0075 | 1 | CC8 | 48h | 3 | 4 | 4 | 3 | 2 | 0 | 0 | 0 | 0 | 0 | 5 |
| ILS AS1-0076 | 1 | CC20 | 48h | 1 | 4 | 4 | 2 | 1 | 0 | 0 | 0 | 0 | 0 | 2.5 |
| ILS AS1-0076 | 1 | CC20 | 48h | 2 | 4 | 4 | 4 | 4 | 4 | 0 | 0 | 0 | 0 | 10 |
| ILS AS1-0076 | 1 | CC20 | 48h | 3 | 4 | 4 | 4 | 4 | 1 | 0 | 0 | 0 | 0 | 7.5 |
| ILS AS1-0086 | 1 | CC20 | 48h | 1 | 4 | 4 | 4 | 4 | 4 | 0 | 0 | 0 | 0 | 10 |
| ILS AS1-0086 | 1 | CC20 | 48h | 2 | 4 | 4 | 4 | 4 | 4 | 0 | 0 | 0 | 0 | 10 |
| ILS AS1-0086 | 1 | CC20 | 48h | 3 | 4 | 4 | 4 | 4 | 4 | 0 | 0 | 0 | 0 | 10 |
| ILS AS1-0077 | 1 | CC29 | 48h | 1 | 4 | 4 | 3 | 2 | 2 | 0 | 0 | 0 | 0 | 5 |
| ILS AS1-0077 | 1 | CC29 | 48h | 2 | 4 | 4 | 2 | 0 | 0 | 0 | 0 | 0 | 0 | 2.5 |
| ILS AS1-0077 | 1 | CC29 | 48h | 3 | 4 | 4 | 2 | 1 | 1 | 0 | 0 | 0 | 0 | 2.5 |
| ILS AS1-0078 | 2 | CC9 | 48h | 1 | 4 | 4 | 4 | 4 | 4 | 0 | 0 | 0 | 0 | 10 |
| ILS AS1-0078 | 2 | CC9 | 48h | 2 | 4 | 4 | 4 | 4 | 4 | 0 | 0 | 0 | 0 | 10 |
| ILS AS1-0078 | 2 | CC9 | 48h | 3 | 4 | 4 | 4 | 4 | 4 | 0 | 0 | 0 | 0 | 10 |
| ILS AS1-0081 | 2 | CC9 | 48h | 1 | 4 | 4 | 4 | 4 | 4 | 0 | 0 | 0 | 0 | 10 |
| ILS AS1-0081 | 2 | CC9 | 48h | 2 | 4 | 4 | 4 | 4 | 4 | 0 | 0 | 0 | 0 | 10 |
| ILS AS1-0081 | 2 | CC9 | 48h | 3 | 4 | 4 | 4 | 4 | 4 | 0 | 0 | 0 | 0 | 10 |
| ILS AS1-0079 | 1 | CC121 | 48h | 1 | 4 | 4 | 4 | 4 | 4 | 0 | 0 | 0 | 0 | 10 |
| ILS AS1-0079 | 1 | CC121 | 48h | 2 | 4 | 4 | 4 | 4 | 4 | 0 | 0 | 0 | 0 | 10 |
| ILS AS1-0079 | 1 | CC121 | 48h | 3 | 4 | 4 | 4 | 4 | 4 | 0 | 0 | 0 | 0 | 10 |
| ILS AS1-0082 | 2 | CC9 | 48h | 1 | 4 | 4 | 4 | 4 | 4 | 0 | 0 | 0 | 0 | 10 |
| ILS AS1-0082 | 2 | CC9 | 48h | 2 | 4 | 4 | 4 | 3 | 1 | 0 | 0 | 0 | 0 | 7.5 |
| ILS AS1-0082 | 2 | CC9 | 48h | 3 | 4 | 4 | 4 | 4 | 4 | 0 | 0 | 0 | 0 | 10 |
| ILS AS1-0080 | 1 | CC121 | 48h | 1 | 4 | 4 | 4 | 4 | 4 | 0 | 0 | 0 | 0 | 10 |
| ILS AS1-0080 | 1 | CC121 | 48h | 2 | 4 | 4 | 4 | 4 | 4 | 0 | 0 | 0 | 0 | 10 |
| ILS AS1-0080 | 1 | CC121 | 48h | 3 | 4 | 4 | 4 | 4 | 4 | 0 | 0 | 0 | 0 | 10 |
| ILS AS1-0083 | 1 | CC204 | 48h | 1 | 4 | 4 | 4 | 4 | 4 | 0 | 0 | 0 | 0 | 10 |
| ILS AS1-0083 | 1 | CC204 | 48h | 2 | 4 | 4 | 4 | 4 | 4 | 0 | 0 | 0 | 0 | 10 |
| ILS AS1-0083 | 1 | CC204 | 48h | 3 | 4 | 4 | 4 | 4 | 4 | 0 | 0 | 0 | 0 | 10 |
| ILS AS1-0090 | 2 | CC9 | 48h | 1 | 4 | 4 | 4 | 4 | 4 | 0 | 0 | 0 | 0 | 10 |
| ILS AS1-0090 | 2 | CC9 | 48h | 2 | 4 | 4 | 4 | 4 | 4 | 0 | 0 | 0 | 0 | 10 |
| ILS AS1-0090 | 2 | CC9 | 48h | 3 | 4 | 4 | 4 | 4 | 4 | 0 | 0 | 0 | 0 | 10 |
| ILS AS1-0085 | 2 | CC9 | 48h | 1 | 4 | 4 | 4 | 4 | 4 | 0 | 0 | 0 | 0 | 10 |
| ILS AS1-0085 | 2 | CC9 | 48h | 2 | 4 | 4 | 4 | 4 | 4 | 0 | 0 | 0 | 0 | 10 |
| ILS AS1-0085 | 2 | CC9 | 48h | 3 | 4 | 4 | 4 | 4 | 4 | 0 | 0 | 0 | 0 | 10 |
| ILS AS1-0084 | 1 | CC204 | 48h | 1 | 4 | 4 | 4 | 4 | 4 | 0 | 0 | 0 | 0 | 10 |
| ILS AS1-0084 | 1 | CC204 | 48h | 2 | 4 | 4 | 4 | 4 | 4 | 0 | 0 | 0 | 0 | 10 |
| ILS AS1-0084 | 1 | CC204 | 48h | 3 | 4 | 4 | 4 | 4 | 4 | 0 | 0 | 0 | 0 | 10 |
| ILS AS1-0087 | 1 | CC204 | 48h | 1 | 4 | 4 | 4 | 4 | 4 | 0 | 0 | 0 | 0 | 10 |
| ILS AS1-0087 | 1 | CC204 | 48h | 2 | 4 | 4 | 4 | 4 | 4 | 0 | 0 | 0 | 0 | 10 |
| ILS AS1-0087 | 1 | CC204 | 48h | 3 | 4 | 4 | 4 | 4 | 4 | 0 | 0 | 0 | 0 | 10 |
| ILS AS1-0088 | 2 | CC9 | 48h | 1 | 4 | 4 | 4 | 4 | 4 | 0 | 0 | 0 | 0 | 10 |
| ILS AS1-0088 | 2 | CC9 | 48h | 2 | 4 | 4 | 4 | 4 | 4 | 0 | 0 | 0 | 0 | 10 |
| ILS AS1-0088 | 2 | CC9 | 48h | 3 | 4 | 4 | 4 | 4 | 4 | 0 | 0 | 0 | 0 | 10 |
| ILS AS1-0089 | 2 | CC9 | 48h | 1 | 4 | 4 | 4 | 4 | 4 | 0 | 0 | 0 | 0 | 10 |
| ILS AS1-0089 | 2 | CC9 | 48h | 2 | 4 | 4 | 4 | 4 | 4 | 0 | 0 | 0 | 0 | 10 |
| ILS AS1-0089 | 2 | CC9 | 48h | 3 | 4 | 4 | 4 | 4 | 4 | 0 | 0 | 0 | 0 | 10 |
| ILS AS1-0091 | 2 | CC9 | 48h | 1 | 4 | 4 | 4 | 4 | 4 | 0 | 0 | 0 | 0 | 10 |
| ILS AS1-0091 | 2 | CC9 | 48h | 2 | 4 | 4 | 4 | 4 | 4 | 0 | 0 | 0 | 0 | 10 |
| ILS AS1-0091 | 2 | CC9 | 48h | 3 | 4 | 4 | 4 | 4 | 4 | 0 | 0 | 0 | 0 | 10 |
| ILS AS1-0006 | 1 | CC121 | 48h | 1 | 4 | 4 | 4 | 4 | 3 | 0 | 0 | 0 | 0 | 10 |
| ILS AS1-0006 | 1 | CC121 | 48h | 2 | 4 | 4 | 4 | 3 | 0 | 0 | 0 | 0 | 0 | 7.5 |
| ILS AS1-0006 | 1 | CC121 | 48h | 3 | 4 | 4 | 4 | 4 | 4 | 0 | 0 | 0 | 0 | 10 |

## Visualize the Benzalkonium chloride data

```
bc %>%
  group_by(readout,serotype) %>%
  summarize(freq=n()) %>%
  ungroup() %>%
  ggplot(aes(x=serotype,y=freq,fill=readout)) +
  geom_bar(stat="identity",position=position_fill(),color="black")
```

```
bc %>%
  group_by(readout,clonal.complex) %>%
  summarize(freq=n()) %>%
  ungroup() %>%
  ggplot(aes(x=clonal.complex,y=freq,fill=readout)) +
  geom_bar(stat="identity",position=position_fill(),color="black")
```

## Model the benzalkonium chloride data by clonal complex and by serotype

```
m_cc <- polr( readout ~ clonal.complex , data=bc)
Anova(m_cc)
```

```
## Analysis of Deviance Table (Type II tests)
## 
## Response: readout
##                LR Chisq Df Pr(>Chisq)    
## clonal.complex   133.07  7  < 2.2e-16 ***
## ---
## Signif. codes:  0 '***' 0.001 '**' 0.01 '*' 0.05 '.' 0.1 ' ' 1
```

```
summary(m_cc) %>%
  coef() %>%
  data.frame() %>%
  tibble::rownames_to_column() %>%
  mutate(p=pnorm(abs(t.value),lower.tail=F)*2)
```

```
## 
## Re-fitting to get Hessian
```

```
##                rowname       Value   Std..Error       t.value            p
## 1   clonal.complexCC20  -1.6610681 8.584646e-01 -1.934929e+00 5.299900e-02
## 2  clonal.complexCC204   0.4828033 7.145783e-01  6.756478e-01 4.992643e-01
## 3   clonal.complexCC29  -4.7710757 9.672995e-01 -4.932367e+00 8.123929e-07
## 4    clonal.complexCC6  -4.3338665 9.188889e-01 -4.716421e+00 2.400297e-06
## 5    clonal.complexCC8  -4.1472808 1.237566e+00 -3.351159e+00 8.047405e-04
## 6   clonal.complexCC89 -37.1321391 3.513028e-14 -1.056984e+15 0.000000e+00
## 7    clonal.complexCC9   0.9144138 6.814421e-01  1.341880e+00 1.796348e-01
## 8                2.5|5  -4.7577916 7.863906e-01 -6.050163e+00 1.446994e-09
## 9                5|7.5  -3.3437450 6.894063e-01 -4.850180e+00 1.233494e-06
## 10              7.5|10  -1.7752801 6.230820e-01 -2.849192e+00 4.383046e-03
```

```
plot(Effect(mod=m_cc,focal.predictors = "clonal.complex"),style="stacked")
```

```
## 
## Re-fitting to get Hessian
```

```
m_serotype <- polr( readout ~ serotype , data=bc)
Anova(m_serotype)
```

```
## Analysis of Deviance Table (Type II tests)
## 
## Response: readout
##          LR Chisq Df Pr(>Chisq)    
## serotype   54.291  2  1.625e-12 ***
## ---
## Signif. codes:  0 '***' 0.001 '**' 0.01 '*' 0.05 '.' 0.1 ' ' 1
```

```
summary(m_serotype) %>%
  coef() %>%
  data.frame() %>%
  tibble::rownames_to_column() %>%
  mutate(p=pnorm(abs(t.value),lower.tail=F)*2)
```

```
## 
## Re-fitting to get Hessian
```

```
##     rowname     Value Std..Error   t.value            p
## 1 serotype2  1.499378  0.3410852  4.395905 1.103121e-05
## 2 serotype4 -2.864957  0.6083366 -4.709494 2.483331e-06
## 3     2.5|5 -2.948590  0.3278769 -8.992981 2.406148e-19
## 4     5|7.5 -2.197786  0.2601423 -8.448400 2.953212e-17
## 5    7.5|10 -1.202743  0.2009402 -5.985576 2.156255e-09
```

```
plot(Effect(mod=m_serotype,focal.predictors = "serotype"),style="stacked")
```

```
## 
## Re-fitting to get Hessian
```

```
1-pchisq(deviance(m_cc),df.residual(m_cc))
```

```
## [1] 0.9971756
```

```
stepAIC(m_cc)
```

```
## Start:  AIC=311.96
## readout ~ clonal.complex
## 
##                  Df    AIC
## <none>              311.96
## - clonal.complex  7 431.03
```

```
## Call:
## polr(formula = readout ~ clonal.complex, data = bc)
## 
## Coefficients:
##  clonal.complexCC20 clonal.complexCC204  clonal.complexCC29 
##          -1.6610681           0.4828033          -4.7710757 
##   clonal.complexCC6   clonal.complexCC8  clonal.complexCC89 
##          -4.3338665          -4.1472808         -37.1321391 
##   clonal.complexCC9 
##           0.9144138 
## 
## Intercepts:
##     2.5|5     5|7.5    7.5|10 
## -4.757792 -3.343745 -1.775280 
## 
## Residual Deviance: 291.963 
## AIC: 311.963
```

# Peracetic acid data

## import and visualize the data for MIC

```
#MIC data 
longlist_mic <- read_excel("data/longlist_mic.xlsx")
MIC <- longlist_mic
# Make readout an ordered factor
MIC$mic <- factor(MIC$mic, ordered=T)
MIC$clonal.complex<-factor(MIC$clonal.complex)
MIC$serotype <- factor(MIC$serotype)
MIC$class <- factor(MIC$class)

MIC %>%
  kable("html") %>%
  kable_styling(bootstrap_options=c("striped",
                                    "hover",
                                    "condensed",
                                    "responsive"))
```

| Strain | serotype | clonal.complex | class | replicate | temperature | time | mic |
| --- | --- | --- | --- | --- | --- | --- | --- |
| ILS AS1-0011 | 2 | CC9 | Strains | 1 | 37° | 48h | 0.2 |
| ILS AS1-0032 | 1 | CC20 | Strains | 1 | 37° | 48h | 0.2 |
| ILS AS1-0051 | 2 | CC9 | Strains | 1 | 37° | 48h | 0.2 |
| ILS AS1-0062 | 2 | CC9 | Strains | 1 | 37° | 48h | 0.2 |
| ILS AS1-0070 | 2 | CC9 | Strains | 1 | 37° | 48h | 0.2 |
| ILS AS1-0071 | 2 | CC9 | Strains | 1 | 37° | 48h | 0.2 |
| ILS AS1-0105 | 2 | CC9 | Strains | 1 | 37° | 48h | 0.1 |
| ILS AS1-0122 | 1 | CC204 | Strains | 1 | 37° | 48h | 0.2 |
| ILS AS1-0011 | 2 | CC9 | Strains | 2 | 37° | 48h | 0.2 |
| ILS AS1-0032 | 1 | CC20 | Strains | 2 | 37° | 48h | 0.2 |
| ILS AS1-0051 | 2 | CC9 | Strains | 2 | 37° | 48h | 0.2 |
| ILS AS1-0062 | 2 | CC9 | Strains | 2 | 37° | 48h | 0.2 |
| ILS AS1-0070 | 2 | CC9 | Strains | 2 | 37° | 48h | 0.2 |
| ILS AS1-0071 | 2 | CC9 | Strains | 2 | 37° | 48h | 0.2 |
| ILS AS1-0105 | 2 | CC9 | Strains | 2 | 37° | 48h | 0.2 |
| ILS AS1-0122 | 1 | CC204 | Strains | 2 | 37° | 48h | 0.2 |
| ILS AS1-0011 | 2 | CC9 | Strains | 3 | 37° | 48h | 0.2 |
| ILS AS1-0032 | 1 | CC20 | Strains | 3 | 37° | 48h | 0.2 |
| ILS AS1-0051 | 2 | CC9 | Strains | 3 | 37° | 48h | 0.2 |
| ILS AS1-0062 | 2 | CC9 | Strains | 3 | 37° | 48h | 0.2 |
| ILS AS1-0070 | 2 | CC9 | Strains | 3 | 37° | 48h | 0.2 |
| ILS AS1-0071 | 2 | CC9 | Strains | 3 | 37° | 48h | 0.2 |
| ILS AS1-0105 | 2 | CC9 | Strains | 3 | 37° | 48h | 0.2 |
| ILS AS1-0122 | 1 | CC204 | Strains | 3 | 37° | 48h | 0.2 |
| ILS AS1-0011 | 2 | CC9 | Strains | 1 | 8° | 168h | 0.2 |
| ILS AS1-0032 | 1 | CC20 | Strains | 1 | 8° | 168h | 0.2 |
| ILS AS1-0051 | 2 | CC9 | Strains | 1 | 8° | 168h | 0.2 |
| ILS AS1-0062 | 2 | CC9 | Strains | 1 | 8° | 168h | 0.2 |
| ILS AS1-0070 | 2 | CC9 | Strains | 1 | 8° | 168h | 0.2 |
| ILS AS1-0071 | 2 | CC9 | Strains | 1 | 8° | 168h | 0.2 |
| ILS AS1-0105 | 2 | CC9 | Strains | 1 | 8° | 168h | 0.2 |
| ILS AS1-0122 | 1 | CC204 | Strains | 1 | 8° | 168h | 0.2 |
| ILS AS1-0011 | 2 | CC9 | Strains | 2 | 8° | 168h | 0.2 |
| ILS AS1-0032 | 1 | CC20 | Strains | 2 | 8° | 168h | 0.2 |
| ILS AS1-0051 | 2 | CC9 | Strains | 2 | 8° | 168h | 0.2 |
| ILS AS1-0062 | 2 | CC9 | Strains | 2 | 8° | 168h | 0.2 |
| ILS AS1-0070 | 2 | CC9 | Strains | 2 | 8° | 168h | 0.2 |
| ILS AS1-0071 | 2 | CC9 | Strains | 2 | 8° | 168h | 0.2 |
| ILS AS1-0105 | 2 | CC9 | Strains | 2 | 8° | 168h | 0.2 |
| ILS AS1-0122 | 1 | CC204 | Strains | 2 | 8° | 168h | 0.2 |
| ILS AS1-0011 | 2 | CC9 | Strains | 3 | 8° | 168h | 0.2 |
| ILS AS1-0032 | 1 | CC20 | Strains | 3 | 8° | 168h | 0.2 |
| ILS AS1-0051 | 2 | CC9 | Strains | 3 | 8° | 168h | 0.2 |
| ILS AS1-0062 | 2 | CC9 | Strains | 3 | 8° | 168h | 0.2 |
| ILS AS1-0070 | 2 | CC9 | Strains | 3 | 8° | 168h | 0.2 |
| ILS AS1-0071 | 2 | CC9 | Strains | 3 | 8° | 168h | 0.2 |
| ILS AS1-0105 | 2 | CC9 | Strains | 3 | 8° | 168h | 0.2 |
| ILS AS1-0122 | 1 | CC204 | Strains | 3 | 8° | 168h | 0.2 |
| ILS AS1-0002 | 1 | CC121 | Strains | 1 | 37° | 48h | 0.2 |
| ILS AS1-0004 | 1 | CC121 | Strains | 1 | 37° | 48h | 0.2 |
| ILS AS1-0079 | 1 | CC121 | Strains | 1 | 37° | 48h | 0.2 |
| ILS AS1-0056 | 1 | CC204 | Strains | 1 | 37° | 48h | 0.2 |
| ILS AS1-0115 | 1 | CC204 | Strains | 1 | 37° | 48h | 0.2 |
| ILS AS1-0123 | 1 | CC121 | Strains | 1 | 37° | 48h | 0.2 |
| ILS AS1-0108 | 4 | CC6 | Strains | 1 | 37° | 48h | 0.2 |
| ILS AS1-0018 | 4 | CC6 | Strains | 1 | 37° | 48h | 0.2 |
| ILS AS1-0002 | 1 | CC121 | Strains | 2 | 37° | 48h | 0.2 |
| ILS AS1-0004 | 1 | CC121 | Strains | 2 | 37° | 48h | 0.2 |
| ILS AS1-0079 | 1 | CC121 | Strains | 2 | 37° | 48h | 0.2 |
| ILS AS1-0056 | 1 | CC204 | Strains | 2 | 37° | 48h | 0.2 |
| ILS AS1-0115 | 1 | CC204 | Strains | 2 | 37° | 48h | 0.2 |
| ILS AS1-0123 | 1 | CC121 | Strains | 2 | 37° | 48h | 0.2 |
| ILS AS1-0108 | 4 | CC6 | Strains | 2 | 37° | 48h | 0.2 |
| ILS AS1-0018 | 4 | CC6 | Strains | 2 | 37° | 48h | 0.2 |
| ILS AS1-0002 | 1 | CC121 | Strains | 3 | 37° | 48h | 0.2 |
| ILS AS1-0004 | 1 | CC121 | Strains | 3 | 37° | 48h | 0.2 |
| ILS AS1-0079 | 1 | CC121 | Strains | 3 | 37° | 48h | 0.2 |
| ILS AS1-0056 | 1 | CC204 | Strains | 3 | 37° | 48h | 0.2 |
| ILS AS1-0115 | 1 | CC204 | Strains | 3 | 37° | 48h | 0.2 |
| ILS AS1-0123 | 1 | CC121 | Strains | 3 | 37° | 48h | 0.2 |
| ILS AS1-0108 | 4 | CC6 | Strains | 3 | 37° | 48h | 0.1 |
| ILS AS1-0018 | 4 | CC6 | Strains | 3 | 37° | 48h | 0.2 |
| ILS AS1-0002 | 1 | CC121 | Strains | 1 | 8° | 168h | 0.1 |
| ILS AS1-0004 | 1 | CC121 | Strains | 1 | 8° | 168h | 0.2 |
| ILS AS1-0079 | 1 | CC121 | Strains | 1 | 8° | 168h | 0.2 |
| ILS AS1-0056 | 1 | CC204 | Strains | 1 | 8° | 168h | 0.2 |
| ILS AS1-0115 | 1 | CC204 | Strains | 1 | 8° | 168h | 0.2 |
| ILS AS1-0123 | 1 | CC121 | Strains | 1 | 8° | 168h | 0.2 |
| ILS AS1-0108 | 4 | CC6 | Strains | 1 | 8° | 168h | 0.2 |
| ILS AS1-0018 | 4 | CC6 | Strains | 1 | 8° | 168h | 0.2 |
| ILS AS1-0002 | 1 | CC121 | Strains | 2 | 8° | 168h | 0.2 |
| ILS AS1-0004 | 1 | CC121 | Strains | 2 | 8° | 168h | 0.2 |
| ILS AS1-0079 | 1 | CC121 | Strains | 2 | 8° | 168h | 0.2 |
| ILS AS1-0056 | 1 | CC204 | Strains | 2 | 8° | 168h | 0.1 |
| ILS AS1-0115 | 1 | CC204 | Strains | 2 | 8° | 168h | 0.1 |
| ILS AS1-0123 | 1 | CC121 | Strains | 2 | 8° | 168h | 0.2 |
| ILS AS1-0108 | 4 | CC6 | Strains | 2 | 8° | 168h | 0.2 |
| ILS AS1-0018 | 4 | CC6 | Strains | 2 | 8° | 168h | 0.2 |
| ILS AS1-0002 | 1 | CC121 | Strains | 3 | 8° | 168h | 0.2 |
| ILS AS1-0004 | 1 | CC121 | Strains | 3 | 8° | 168h | 0.2 |
| ILS AS1-0079 | 1 | CC121 | Strains | 3 | 8° | 168h | 0.2 |
| ILS AS1-0056 | 1 | CC204 | Strains | 3 | 8° | 168h | 0.2 |
| ILS AS1-0115 | 1 | CC204 | Strains | 3 | 8° | 168h | 0.2 |
| ILS AS1-0123 | 1 | CC121 | Strains | 3 | 8° | 168h | 0.2 |
| ILS AS1-0108 | 4 | CC6 | Strains | 3 | 8° | 168h | 0.2 |
| ILS AS1-0018 | 4 | CC6 | Strains | 3 | 8° | 168h | 0.2 |
| NA | NA | CC204 | comparative strain | 1 | 37° | 48h | 0.2 |
| NA | NA | CC204 | comparative strain | 1 | 37° | 48h | 0.2 |
| NA | NA | CC204 | comparative strain | 1 | 37° | 48h | 0.1 |
| NA | NA | CC9 | comparative strain | 1 | 37° | 48h | 0.2 |
| NA | NA | CC9 | comparative strain | 1 | 37° | 48h | 0.2 |
| NA | NA | CC9 | comparative strain | 1 | 37° | 48h | 0.2 |
| NA | NA | CC9 | comparative strain | 1 | 37° | 48h | 0.2 |
| NA | NA | CC121 | comparative strain | 1 | 37° | 48h | 0.2 |
| NA | NA | CC6 | comparative strain | 1 | 37° | 48h | 0.2 |
| NA | NA | CC204 | comparative strain | 2 | 37° | 48h | 0.2 |
| NA | NA | CC204 | comparative strain | 2 | 37° | 48h | 0.2 |
| NA | NA | CC204 | comparative strain | 2 | 37° | 48h | 0.2 |
| NA | NA | CC9 | comparative strain | 2 | 37° | 48h | 0.2 |
| NA | NA | CC9 | comparative strain | 2 | 37° | 48h | 0.2 |
| NA | NA | CC9 | comparative strain | 2 | 37° | 48h | 0.2 |
| NA | NA | CC9 | comparative strain | 2 | 37° | 48h | 0.2 |
| NA | NA | CC121 | comparative strain | 2 | 37° | 48h | 0.2 |
| NA | NA | CC6 | comparative strain | 2 | 37° | 48h | 0.2 |
| NA | NA | CC204 | comparative strain | 3 | 37° | 48h | 0.1 |
| NA | NA | CC204 | comparative strain | 3 | 37° | 48h | 0.2 |
| NA | NA | CC204 | comparative strain | 3 | 37° | 48h | 0.2 |
| NA | NA | CC9 | comparative strain | 3 | 37° | 48h | 0.2 |
| NA | NA | CC9 | comparative strain | 3 | 37° | 48h | 0.2 |
| NA | NA | CC9 | comparative strain | 3 | 37° | 48h | 0.2 |
| NA | NA | CC9 | comparative strain | 3 | 37° | 48h | 0.2 |
| NA | NA | CC121 | comparative strain | 3 | 37° | 48h | 0.2 |
| NA | NA | CC6 | comparative strain | 3 | 37° | 48h | 0.2 |
| NA | NA | CC204 | comparative strain | 1 | 8° | 168h | 0.2 |
| NA | NA | CC204 | comparative strain | 1 | 8° | 168h | 0.2 |
| NA | NA | CC204 | comparative strain | 1 | 8° | 168h | 0.2 |
| NA | NA | CC9 | comparative strain | 1 | 8° | 168h | 0.2 |
| NA | NA | CC9 | comparative strain | 1 | 8° | 168h | 0.2 |
| NA | NA | CC9 | comparative strain | 1 | 8° | 168h | 0.2 |
| NA | NA | CC9 | comparative strain | 1 | 8° | 168h | 0.2 |
| NA | NA | CC121 | comparative strain | 1 | 8° | 168h | 0.2 |
| NA | NA | CC6 | comparative strain | 1 | 8° | 168h | 0.2 |
| NA | NA | CC204 | comparative strain | 2 | 8° | 168h | 0.2 |
| NA | NA | CC204 | comparative strain | 2 | 8° | 168h | 0.2 |
| NA | NA | CC204 | comparative strain | 2 | 8° | 168h | 0.2 |
| NA | NA | CC9 | comparative strain | 2 | 8° | 168h | 0.2 |
| NA | NA | CC9 | comparative strain | 2 | 8° | 168h | 0.2 |
| NA | NA | CC9 | comparative strain | 2 | 8° | 168h | 0.2 |
| NA | NA | CC9 | comparative strain | 2 | 8° | 168h | 0.2 |
| NA | NA | CC121 | comparative strain | 2 | 8° | 168h | 0.2 |
| NA | NA | CC6 | comparative strain | 2 | 8° | 168h | 0.2 |
| NA | NA | CC204 | comparative strain | 3 | 8° | 168h | 0.2 |
| NA | NA | CC204 | comparative strain | 3 | 8° | 168h | 0.1 |
| NA | NA | CC204 | comparative strain | 3 | 8° | 168h | 0.1 |
| NA | NA | CC9 | comparative strain | 3 | 8° | 168h | 0.1 |
| NA | NA | CC9 | comparative strain | 3 | 8° | 168h | 0.2 |
| NA | NA | CC9 | comparative strain | 3 | 8° | 168h | 0.2 |
| NA | NA | CC9 | comparative strain | 3 | 8° | 168h | 0.2 |
| NA | NA | CC121 | comparative strain | 3 | 8° | 168h | 0.2 |
| NA | NA | CC6 | comparative strain | 3 | 8° | 168h | 0.2 |

```
MIC %>%
  group_by(mic,clonal.complex,temperature, class) %>%
  summarize(freq=n()) %>%
  ungroup() %>%
  ggplot(aes(x=clonal.complex,y=freq,fill=mic)) +
  geom_bar(stat="identity",position=position_fill(),color="black")+
  facet_grid(temperature~ class)
```

This does not need any modelling to see that there is no significant difference

## import and visualize the data for MBC

```
#MIC data 
longlist_mbk_wasser_bhi <- read_excel("data/longlist_mbk_wasser_bhi.xlsx")
MBC <- longlist_mbk_wasser_bhi
# Make readout an ordered factor
MBC$mbc <- factor(MBC$mbc, ordered=T)
MBC$clonal.complex<-factor(MBC$clonal.complex)
MBC$serotype <- factor(MBC$serotype)
MBC$class<- factor(MBC$class)
MBC$temperature <-factor(MBC$temperature)
MBC$medium <- factor(MBC$Medium)

MBC %>%
  kable("html") %>%
  kable_styling(bootstrap_options=c("striped",
                                    "hover",
                                    "condensed",
                                    "responsive"))
```

| Strain | serotype | clonal.complex | class | Medium | temperature | time | mbc | medium |
| --- | --- | --- | --- | --- | --- | --- | --- | --- |
| H1 | NA | CC204 | comparative strain | bhi | 37° | 48h | 1.2 | bhi |
| H1 | NA | CC204 | comparative strain | bhi | 37° | 48h | 1.2 | bhi |
| H1 | NA | CC204 | comparative strain | bhi | 37° | 48h | 1.6 | bhi |
| H1 | NA | CC204 | comparative strain | water | 37° | 48h | 0.1 | water |
| H1 | NA | CC204 | comparative strain | water | 37° | 48h | 0.2 | water |
| H1 | NA | CC204 | comparative strain | water | 37° | 48h | 0.2 | water |
| H2 | NA | CC204 | comparative strain | bhi | 37° | 48h | 1.2 | bhi |
| H2 | NA | CC204 | comparative strain | bhi | 37° | 48h | 1.2 | bhi |
| H2 | NA | CC204 | comparative strain | bhi | 37° | 48h | 1.6 | bhi |
| H2 | NA | CC204 | comparative strain | water | 37° | 48h | 0.2 | water |
| H2 | NA | CC204 | comparative strain | water | 37° | 48h | 0.2 | water |
| H2 | NA | CC204 | comparative strain | water | 37° | 48h | 0.4 | water |
| H3 | NA | CC204 | comparative strain | bhi | 37° | 48h | 1.2 | bhi |
| H3 | NA | CC204 | comparative strain | bhi | 37° | 48h | 1.6 | bhi |
| H3 | NA | CC204 | comparative strain | bhi | 37° | 48h | 1.6 | bhi |
| H3 | NA | CC204 | comparative strain | water | 37° | 48h | 0.1 | water |
| H3 | NA | CC204 | comparative strain | water | 37° | 48h | 0.1 | water |
| H3 | NA | CC204 | comparative strain | water | 37° | 48h | 0.4 | water |
| H4 | NA | CC9 | comparative strain | bhi | 37° | 48h | 1.2 | bhi |
| H4 | NA | CC9 | comparative strain | bhi | 37° | 48h | 1.6 | bhi |
| H4 | NA | CC9 | comparative strain | bhi | 37° | 48h | 1.6 | bhi |
| H4 | NA | CC9 | comparative strain | water | 37° | 48h | 0.2 | water |
| H4 | NA | CC9 | comparative strain | water | 37° | 48h | 0.2 | water |
| H4 | NA | CC9 | comparative strain | water | 37° | 48h | 0.2 | water |
| H5 | NA | CC9 | comparative strain | bhi | 37° | 48h | 1.6 | bhi |
| H5 | NA | CC9 | comparative strain | bhi | 37° | 48h | 1.6 | bhi |
| H5 | NA | CC9 | comparative strain | bhi | 37° | 48h | 1.6 | bhi |
| H5 | NA | CC9 | comparative strain | water | 37° | 48h | 0.1 | water |
| H5 | NA | CC9 | comparative strain | water | 37° | 48h | 0.2 | water |
| H5 | NA | CC9 | comparative strain | water | 37° | 48h | 0.2 | water |
| H6 | NA | CC9 | comparative strain | bhi | 37° | 48h | 1.2 | bhi |
| H6 | NA | CC9 | comparative strain | bhi | 37° | 48h | 1.6 | bhi |
| H6 | NA | CC9 | comparative strain | bhi | 37° | 48h | 1.6 | bhi |
| H6 | NA | CC9 | comparative strain | water | 37° | 48h | 0.1 | water |
| H6 | NA | CC9 | comparative strain | water | 37° | 48h | 0.2 | water |
| H6 | NA | CC9 | comparative strain | water | 37° | 48h | 0.4 | water |
| H7 | NA | CC9 | comparative strain | bhi | 37° | 48h | 1.2 | bhi |
| H7 | NA | CC9 | comparative strain | bhi | 37° | 48h | 1.2 | bhi |
| H7 | NA | CC9 | comparative strain | bhi | 37° | 48h | 1.6 | bhi |
| H7 | NA | CC9 | comparative strain | water | 37° | 48h | 0.1 | water |
| H7 | NA | CC9 | comparative strain | water | 37° | 48h | 0.2 | water |
| H7 | NA | CC9 | comparative strain | water | 37° | 48h | 0.2 | water |
| H8 | NA | CC121 | comparative strain | bhi | 37° | 48h | 1.2 | bhi |
| H8 | NA | CC121 | comparative strain | bhi | 37° | 48h | 1.2 | bhi |
| H8 | NA | CC121 | comparative strain | bhi | 37° | 48h | 1.2 | bhi |
| H8 | NA | CC121 | comparative strain | water | 37° | 48h | 0.1 | water |
| H8 | NA | CC121 | comparative strain | water | 37° | 48h | 0.2 | water |
| H8 | NA | CC121 | comparative strain | water | 37° | 48h | 0.2 | water |
| H9 | NA | CC6 | comparative strain | bhi | 37° | 48h | 1.2 | bhi |
| H9 | NA | CC6 | comparative strain | bhi | 37° | 48h | 1.2 | bhi |
| H9 | NA | CC6 | comparative strain | bhi | 37° | 48h | 1.6 | bhi |
| H9 | NA | CC6 | comparative strain | water | 37° | 48h | 0.1 | water |
| H9 | NA | CC6 | comparative strain | water | 37° | 48h | 0.2 | water |
| H9 | NA | CC6 | comparative strain | water | 37° | 48h | 0.2 | water |
| ILS AS1-0002 | 1 | CC121 | strain | bhi | 37° | 48h | 1.2 | bhi |
| ILS AS1-0002 | 1 | CC121 | strain | bhi | 37° | 48h | 1.2 | bhi |
| ILS AS1-0002 | 1 | CC121 | strain | bhi | 37° | 48h | 1.6 | bhi |
| ILS AS1-0002 | 1 | CC121 | strain | water | 37° | 48h | 0.2 | water |
| ILS AS1-0002 | 1 | CC121 | strain | water | 37° | 48h | 0.4 | water |
| ILS AS1-0002 | 1 | CC121 | strain | water | 37° | 48h | 0.05 | water |
| ILS AS1-0004 | 1 | CC204 | strain | bhi | 37° | 48h | 1.2 | bhi |
| ILS AS1-0004 | 1 | CC204 | strain | bhi | 37° | 48h | 1.2 | bhi |
| ILS AS1-0004 | 1 | CC204 | strain | bhi | 37° | 48h | 1.6 | bhi |
| ILS AS1-0004 | 1 | CC204 | strain | water | 37° | 48h | 0.2 | water |
| ILS AS1-0004 | 1 | CC204 | strain | water | 37° | 48h | 0.4 | water |
| ILS AS1-0004 | 1 | CC204 | strain | water | 37° | 48h | 0.05 | water |
| ILS AS1-0011 | 2 | CC9 | strain | bhi | 37° | 48h | 1.2 | bhi |
| ILS AS1-0011 | 2 | CC9 | strain | bhi | 37° | 48h | 1.2 | bhi |
| ILS AS1-0011 | 2 | CC9 | strain | bhi | 37° | 48h | 1.6 | bhi |
| ILS AS1-0011 | 2 | CC9 | strain | water | 37° | 48h | 0.4 | water |
| ILS AS1-0011 | 2 | CC9 | strain | water | 37° | 48h | 0.4 | water |
| ILS AS1-0011 | 2 | CC9 | strain | water | 37° | 48h | 0.05 | water |
| ILS AS1-0018 | 4 | CC6 | strain | bhi | 37° | 48h | 1.2 | bhi |
| ILS AS1-0018 | 4 | CC6 | strain | bhi | 37° | 48h | 1.2 | bhi |
| ILS AS1-0018 | 4 | CC6 | strain | bhi | 37° | 48h | 1.6 | bhi |
| ILS AS1-0018 | 4 | CC6 | strain | water | 37° | 48h | 0.2 | water |
| ILS AS1-0018 | 4 | CC6 | strain | water | 37° | 48h | 0.2 | water |
| ILS AS1-0018 | 4 | CC6 | strain | water | 37° | 48h | 0.05 | water |
| ILS AS1-0032 | 1 | CC20 | strain | bhi | 37° | 48h | 1.2 | bhi |
| ILS AS1-0032 | 1 | CC20 | strain | bhi | 37° | 48h | 1.6 | bhi |
| ILS AS1-0032 | 1 | CC20 | strain | bhi | 37° | 48h | 1.6 | bhi |
| ILS AS1-0032 | 1 | CC20 | strain | water | 37° | 48h | 0.2 | water |
| ILS AS1-0032 | 1 | CC20 | strain | water | 37° | 48h | 0.4 | water |
| ILS AS1-0032 | 1 | CC20 | strain | water | 37° | 48h | 0.05 | water |
| ILS AS1-0051 | 2 | CC9 | strain | bhi | 37° | 48h | 1.2 | bhi |
| ILS AS1-0051 | 2 | CC9 | strain | bhi | 37° | 48h | 1.6 | bhi |
| ILS AS1-0051 | 2 | CC9 | strain | bhi | 37° | 48h | 1.6 | bhi |
| ILS AS1-0051 | 2 | CC9 | strain | water | 37° | 48h | 0.2 | water |
| ILS AS1-0051 | 2 | CC9 | strain | water | 37° | 48h | 0.4 | water |
| ILS AS1-0051 | 2 | CC9 | strain | water | 37° | 48h | 0.05 | water |
| ILS AS1-0056 | 1 | CC204 | strain | bhi | 37° | 48h | 1.2 | bhi |
| ILS AS1-0056 | 1 | CC204 | strain | bhi | 37° | 48h | 1.2 | bhi |
| ILS AS1-0056 | 1 | CC204 | strain | bhi | 37° | 48h | 1.6 | bhi |
| ILS AS1-0056 | 1 | CC204 | strain | water | 37° | 48h | 0.2 | water |
| ILS AS1-0056 | 1 | CC204 | strain | water | 37° | 48h | 0.4 | water |
| ILS AS1-0056 | 1 | CC204 | strain | water | 37° | 48h | 0.05 | water |
| ILS AS1-0062 | 2 | CC9 | strain | bhi | 37° | 48h | 1.2 | bhi |
| ILS AS1-0062 | 2 | CC9 | strain | bhi | 37° | 48h | 1.2 | bhi |
| ILS AS1-0062 | 2 | CC9 | strain | bhi | 37° | 48h | 1.6 | bhi |
| ILS AS1-0062 | 2 | CC9 | strain | water | 37° | 48h | 0.2 | water |
| ILS AS1-0062 | 2 | CC9 | strain | water | 37° | 48h | 0.2 | water |
| ILS AS1-0062 | 2 | CC9 | strain | water | 37° | 48h | 0.05 | water |
| ILS AS1-0070 | 2 | CC9 | strain | bhi | 37° | 48h | 1.6 | bhi |
| ILS AS1-0070 | 2 | CC9 | strain | bhi | 37° | 48h | 1.6 | bhi |
| ILS AS1-0070 | 2 | CC9 | strain | bhi | 37° | 48h | 1.6 | bhi |
| ILS AS1-0070 | 2 | CC9 | strain | water | 37° | 48h | 0.2 | water |
| ILS AS1-0070 | 2 | CC9 | strain | water | 37° | 48h | 0.4 | water |
| ILS AS1-0070 | 2 | CC9 | strain | water | 37° | 48h | 0.05 | water |
| ILS AS1-0071 | 2 | CC9 | strain | bhi | 37° | 48h | 1.6 | bhi |
| ILS AS1-0071 | 2 | CC9 | strain | bhi | 37° | 48h | 1.6 | bhi |
| ILS AS1-0071 | 2 | CC9 | strain | bhi | 37° | 48h | 1.6 | bhi |
| ILS AS1-0071 | 2 | CC9 | strain | water | 37° | 48h | 0.2 | water |
| ILS AS1-0071 | 2 | CC9 | strain | water | 37° | 48h | 0.4 | water |
| ILS AS1-0071 | 2 | CC9 | strain | water | 37° | 48h | 0.05 | water |
| ILS AS1-0079 | 1 | CC121 | strain | bhi | 37° | 48h | 1.6 | bhi |
| ILS AS1-0079 | 1 | CC121 | strain | bhi | 37° | 48h | 1.6 | bhi |
| ILS AS1-0079 | 1 | CC121 | strain | bhi | 37° | 48h | 1.6 | bhi |
| ILS AS1-0079 | 1 | CC121 | strain | water | 37° | 48h | 0.4 | water |
| ILS AS1-0079 | 1 | CC121 | strain | water | 37° | 48h | 0.4 | water |
| ILS AS1-0079 | 1 | CC121 | strain | water | 37° | 48h | 0.05 | water |
| ILS AS1-0105 | 2 | CC9 | strain | bhi | 37° | 48h | 1.2 | bhi |
| ILS AS1-0105 | 2 | CC9 | strain | bhi | 37° | 48h | 1.6 | bhi |
| ILS AS1-0105 | 2 | CC9 | strain | bhi | 37° | 48h | 1.6 | bhi |
| ILS AS1-0105 | 2 | CC9 | strain | water | 37° | 48h | 0.2 | water |
| ILS AS1-0105 | 2 | CC9 | strain | water | 37° | 48h | 0.2 | water |
| ILS AS1-0105 | 2 | CC9 | strain | water | 37° | 48h | 0.05 | water |
| ILS AS1-0108 | 4 | CC6 | strain | bhi | 37° | 48h | 1.6 | bhi |
| ILS AS1-0108 | 4 | CC6 | strain | bhi | 37° | 48h | 1.6 | bhi |
| ILS AS1-0108 | 4 | CC6 | strain | bhi | 37° | 48h | 1.6 | bhi |
| ILS AS1-0108 | 4 | CC6 | strain | water | 37° | 48h | 0.2 | water |
| ILS AS1-0108 | 4 | CC6 | strain | water | 37° | 48h | 0.2 | water |
| ILS AS1-0108 | 4 | CC6 | strain | water | 37° | 48h | 0.05 | water |
| ILS AS1-0115 | 1 | CC204 | strain | bhi | 37° | 48h | 1.2 | bhi |
| ILS AS1-0115 | 1 | CC204 | strain | bhi | 37° | 48h | 1.2 | bhi |
| ILS AS1-0115 | 1 | CC204 | strain | bhi | 37° | 48h | 1.6 | bhi |
| ILS AS1-0115 | 1 | CC204 | strain | water | 37° | 48h | 0.2 | water |
| ILS AS1-0115 | 1 | CC204 | strain | water | 37° | 48h | 0.2 | water |
| ILS AS1-0115 | 1 | CC204 | strain | water | 37° | 48h | 0.05 | water |
| ILS AS1-0122 | 1 | CC204 | strain | bhi | 37° | 48h | 1.2 | bhi |
| ILS AS1-0122 | 1 | CC204 | strain | bhi | 37° | 48h | 1.2 | bhi |
| ILS AS1-0122 | 1 | CC204 | strain | bhi | 37° | 48h | 1.6 | bhi |
| ILS AS1-0122 | 1 | CC204 | strain | water | 37° | 48h | 0.2 | water |
| ILS AS1-0122 | 1 | CC204 | strain | water | 37° | 48h | 0.2 | water |
| ILS AS1-0122 | 1 | CC204 | strain | water | 37° | 48h | 0.05 | water |
| ILS AS1-0124 | 1 | CC204 | strain | bhi | 37° | 48h | 1.2 | bhi |
| ILS AS1-0124 | 1 | CC204 | strain | bhi | 37° | 48h | 1.6 | bhi |
| ILS AS1-0124 | 1 | CC204 | strain | bhi | 37° | 48h | 1.6 | bhi |
| ILS AS1-0124 | 1 | CC204 | strain | water | 37° | 48h | 0.2 | water |
| ILS AS1-0124 | 1 | CC204 | strain | water | 37° | 48h | 0.2 | water |
| ILS AS1-0124 | 1 | CC204 | strain | water | 37° | 48h | 0.05 | water |
| H1 | NA | CC204 | comparative strain | water | 37° | 48h | 0.05 | water |
| H2 | NA | CC204 | comparative strain | water | 37° | 48h | 0.05 | water |
| H3 | NA | CC204 | comparative strain | water | 37° | 48h | 0.05 | water |
| H4 | NA | CC9 | comparative strain | water | 37° | 48h | 0.05 | water |
| H5 | NA | CC9 | comparative strain | water | 37° | 48h | 0.05 | water |
| H6 | NA | CC9 | comparative strain | water | 37° | 48h | 0.05 | water |
| H7 | NA | CC9 | comparative strain | water | 37° | 48h | 0.05 | water |
| H8 | NA | CC121 | comparative strain | water | 37° | 48h | 0.05 | water |
| H9 | NA | CC6 | comparative strain | water | 37° | 48h | 0.05 | water |

```
#EXPLORE THE DATA
hist(as.numeric(MBC$mbc))
```

```
scatterplot(mbc ~ clonal.complex, data=MBC)
```

```
scatterplot(mbc ~ temperature, data=MBC)
```

```
#make nice Graphs
MBC %>%
  group_by(mbc,medium) %>%
  summarize(freq=n()) %>%
  ungroup() %>%
  ggplot(aes(x=medium,y=freq,fill=mbc)) +
  geom_bar(stat="identity",position=position_fill(),color="black")
```

```
MBC %>%
  group_by(mbc,medium, class) %>%
  summarize(freq=n()) %>%
  ungroup() %>%
  ggplot(aes(x=medium,y=freq,fill=mbc)) +
  geom_bar(stat="identity",position=position_fill(),color="black")+
  facet_grid(class~.)
```

```
MBC %>%
  group_by(mbc,clonal.complex,medium , class) %>%
  summarize(freq=n()) %>%
  ungroup() %>%
  ggplot(aes(x=clonal.complex,y=freq,fill=mbc)) +
  geom_bar(stat="identity",position=position_fill(),color="black")+
  facet_grid(medium ~ class)
```

## model the MBC data

```
m_mbc_full <- polr( mbc ~ clonal.complex + class + medium , data= MBC, Hess=TRUE)
Anova(m_mbc_full)
```

```
## Analysis of Deviance Table (Type II tests)
## 
## Response: mbc
##                LR Chisq Df Pr(>Chisq)    
## clonal.complex    2.456  4     0.6525    
## class             1.931  1     0.1647    
## medium          221.756  1     <2e-16 ***
## ---
## Signif. codes:  0 '***' 0.001 '**' 0.01 '*' 0.05 '.' 0.1 ' ' 1
```

```
summary(m_mbc_full) %>%
  coef() %>%
  data.frame() %>%
  tibble::rownames_to_column() %>%
  mutate(p=pnorm(abs(t.value),lower.tail=F)*2)
```

```
##                rowname       Value Std..Error      t.value         p
## 1   clonal.complexCC20   0.3391597  0.9696301    0.3497826 0.7265019
## 2  clonal.complexCC204  -0.2371231  0.5274846   -0.4495356 0.6530453
## 3    clonal.complexCC6  -0.1742345  0.6224160   -0.2799325 0.7795293
## 4    clonal.complexCC9   0.2818360  0.5154050    0.5468244 0.5844994
## 5          classstrain   0.4405205  0.3181092    1.3848091 0.1661109
## 6          mediumwater -35.1697517  0.2574623 -136.6015687 0.0000000
## 7             0.05|0.1 -35.7982508  0.2578081 -138.8561925 0.0000000
## 8              0.1|0.2 -35.3722116  0.2722505 -129.9252389 0.0000000
## 9              0.2|0.4 -33.2674670  0.3740035  -88.9496091 0.0000000
## 10             0.4|1.2 -22.4420453 45.6409301   -0.4917088 0.6229252
## 11             1.2|1.6   0.1199176  0.5222923    0.2295987 0.8184036
```

```
confint(m_mbc_full)
```

```
## Waiting for profiling to be done...
```

```
##                          2.5 %    97.5 %
## clonal.complexCC20  -1.5459745 2.3313457
## clonal.complexCC204 -1.2814693 0.8004252
## clonal.complexCC6   -1.4040810 1.0500083
## clonal.complexCC9   -0.7362732 1.2989058
## classstrain         -0.1805841 1.0690758
## mediumwater                 NA        NA
```

```
1-pchisq(deviance(m_mbc_full),df.residual(m_mbc_full))
```

```
## [1] 2.674527e-13
```

```
#test if we could drop terms from the model.
#this confirms that medium is the only relevant factor 
stepAIC(m_mbc_full)
```

```
## Start:  AIC=330.04
## mbc ~ clonal.complex + class + medium
## 
##                  Df    AIC
## - clonal.complex  4 324.50
## - class           1 329.97
## <none>              330.04
## - medium          1 549.79
## 
## Step:  AIC=324.5
## mbc ~ class + medium
## 
##          Df    AIC
## - class   1 324.39
## <none>      324.50
## - medium  1 542.25
## 
## Step:  AIC=324.39
## mbc ~ medium
## 
##          Df    AIC
## <none>      324.39
## - medium  1 542.29
```

```
## Call:
## polr(formula = mbc ~ medium, data = MBC, Hess = TRUE)
## 
## Coefficients:
## mediumwater 
##   -20.89182 
## 
## Intercepts:
##    0.05|0.1     0.1|0.2     0.2|0.4     0.4|1.2     1.2|1.6 
## -21.7505070 -21.3271850 -19.2825002 -10.3355081  -0.1871213 
## 
## Residual Deviance: 312.3879 
## AIC: 324.3879
```

# as a supplement, import the MBC / MIC data for all strains

```
longlist_ganzes_kollektiv <- read_excel("data/longlist_ganzes kollektiv.xlsx")
longlist_ganzes_kollektiv %>%
  kable("html") %>%
  kable_styling(bootstrap_options=c("striped",
                                    "hover",
                                    "condensed",
                                    "responsive"))
```

| strain | CC | MIC | MBC |
| --- | --- | --- | --- |
| ILS AS1-0001 | CC9 | 0.2 | 1.2 |
| ILS AS1-0002 | CC121 | 0.2 | 1.2 |
| ILS AS1-0003 | CC29 | 0.2 | 1.2 |
| ILS AS1-0004 | CC204 | 0.2 | 1.2 |
| ILS AS1-0005 | CC9 | 0.2 | 1.2 |
| ILS AS1-0006 | CC121 | 0.2 | 1.2 |
| ILS AS1-0007 | CC204 | 0.2 | 1.2 |
| ILS AS1-0008 | CC9 | 0.2 | 1.2 |
| ILS AS1-0009 | CC9 | 0.2 | 1.2 |
| ILS AS1-0010 | CC9 | 0.2 | 1.2 |
| ILS AS1-0011 | CC9 | 0.2 | 1.2 |
| ILS AS1-0012 | CC9 | 0.2 | 1.2 |
| ILS AS1-0013 | CC9 | 0.2 | 1.2 |
| ILS AS1-0014 | CC9 | 0.2 | 1.2 |
| ILS AS1-0015 | CC9 | 0.2 | 1.2 |
| ILS AS1-0016 | CC9 | 0.2 | 1.6 |
| ILS AS1-0017 | CC9 | 0.2 | 1.2 |
| ILS AS1-0018 | CC6 | 0.2 | 1.2 |
| ILS AS1-0019 | CC121 | 0.2 | 1.2 |
| ILS AS1-0020 | CC204 | 0.2 | 1.2 |
| ILS AS1-0021 | CC204 | 0.2 | 1.2 |
| ILS AS1-0022 | CC9 | 0.2 | 1.2 |
| ILS AS1-0023 | CC204 | 0.2 | 1.2 |
| ILS AS1-0024 | CC9 | 0.2 | 1.2 |
| ILS AS1-0025 | CC9 | 0.2 | 1.2 |
| ILS AS1-0026 | CC204 | 0.2 | 1.2 |
| ILS AS1-0027 | CC9 | 0.2 | 1.2 |
| ILS AS1-0028 | CC6 | 0.2 | 1.2 |
| ILS AS1-0029 | CC9 | 0.2 | 1.2 |
| ILS AS1-0030 | CC9 | 0.2 | 1.2 |
| ILS AS1-0031 | CC204 | 0.2 | 1.2 |
| ILS AS1-0032 | CC20 | 0.2 | 1.6 |
| ILS AS1-0033 | CC20 | 0.2 | 1.2 |
| ILS AS1-0034 | CC9 | 0.2 | 1.2 |
| ILS AS1-0035 | CC9 | 0.2 | 1.2 |
| ILS AS1-0036 | CC9 | 0.2 | 1.2 |
| ILS AS1-0037 | CC9 | 0.2 | 1.2 |
| ILS AS1-0038 | CC9 | 0.2 | 1.2 |
| ILS AS1-0039 | CC121 | 0.2 | 1.2 |
| ILS AS1-0040 | CC204 | 0.2 | 1.2 |
| ILS AS1-0041 | CC9 | 0.2 | 1.2 |
| ILS AS1-0042 | CC9 | 0.2 | 1.2 |
| ILS AS1-0043 | CC29 | 0.2 | 1.2 |
| ILS AS1-0044 | CC9 | 0.2 | 1.2 |
| ILS AS1-0045 | CC9 | 0.2 | 1.2 |
| ILS AS1-0046 | CC9 | 0.2 | 1.2 |
| ILS AS1-0047 | CC204 | 0.2 | 1.2 |
| ILS AS1-0048 | CC9 | 0.2 | 1.2 |
| ILS AS1-0049 | CC9 | 0.2 | 1.2 |
| ILS AS1-0050 | CC9 | 0.1 | 1.6 |
| ILS AS1-0051 | CC9 | 0.2 | 1.2 |
| ILS AS1-0052 | CC9 | 0.2 | 1.6 |
| ILS AS1-0053 | CC9 | 0.2 | 1.6 |
| ILS AS1-0054 | CC204 | 0.2 | 1.6 |
| ILS AS1-0055 | CC204 | 0.2 | 1.6 |
| ILS AS1-0056 | CC204 | 0.2 | 1.2 |
| ILS AS1-0057 | CC9 | 0.2 | 1.6 |
| ILS AS1-0058 | CC9 | 0.2 | 1.6 |
| ILS AS1-0059 | CC9 | 0.1 | 1.6 |
| ILS AS1-0060 | CC9 | 0.2 | 1.6 |
| ILS AS1-0061 | CC9 | 0.2 | 1.6 |
| ILS AS1-0062 | CC9 | 0.2 | 1.2 |
| ILS AS1-0063 | CC9 | 0.2 | 1.6 |
| ILS AS1-0064 | CC9 | 0.2 | 1.6 |
| ILS AS1-0065 | CC9 | 0.2 | 1.2 |
| ILS AS1-0066 | CC89 | 0.2 | 0.8 |
| ILS AS1-0067 | CC9 | 0.2 | 0.8 |
| ILS AS1-0068 | CC9 | 0.2 | 1.6 |
| ILS AS1-0069 | CC29 | 0.2 | 1.6 |
| ILS AS1-0070 | CC9 | 0.2 | 1.6 |
| ILS AS1-0071 | CC9 | 0.2 | 1.6 |
| ILS AS1-0072 | CC9 | 0.2 | 1.2 |
| ILS AS1-0073 | CC6 | 0.2 | 1.2 |
| ILS AS1-0074 | CC29 | 0.2 | 1.2 |
| ILS AS1-0075 | CC8 | 0.1 | 1.6 |
| ILS AS1-0076 | CC20 | 0.1 | 1.6 |
| ILS AS1-0077 | CC29 | 0.1 | 1.6 |
| ILS AS1-0078 | CC9 | 0.1 | 1.6 |
| ILS AS1-0079 | CC121 | 0.2 | 1.6 |
| ILS AS1-0080 | CC121 | 0.1 | 1.2 |
| ILS AS1-0081 | CC9 | 0.1 | 1.2 |
| ILS AS1-0082 | CC9 | 0.1 | 1.2 |
| ILS AS1-0083 | CC204 | 0.1 | 1.2 |
| ILS AS1-0084 | CC204 | 0.1 | 1.6 |
| ILS AS1-0085 | CC9 | 0.1 | 1.6 |
| ILS AS1-0086 | CC20 | 0.1 | 1.2 |
| ILS AS1-0087 | CC204 | 0.1 | 1.2 |
| ILS AS1-0088 | CC9 | 0.1 | 1.2 |
| ILS AS1-0089 | CC9 | 0.2 | 1.2 |
| ILS AS1-0090 | CC9 | 0.2 | 1.2 |
| ILS AS1-0091 | CC9 | 0.2 | 1.2 |
| ILS AS1-0092 | CC9 | 0.2 | 1.6 |
| ILS AS1-0093 | CC9 | 0.2 | 1.6 |
| ILS AS1-0094 | CC9 | 0.2 | 1.6 |
| ILS AS1-0095 | CC9 | 0.2 | 1.6 |
| ILS AS1-0096 | CC9 | 0.2 | 1.6 |
| ILS AS1-0097 | CC9 | 0.2 | 1.2 |
| ILS AS1-0098 | CC204 | 0.2 | 1.2 |
| ILS AS1-0099 | CC204 | 0.2 | 1.2 |
| ILS AS1-0100 | CC9 | 0.2 | 1.2 |
| ILS AS1-0101 | CC9 | 0.2 | 1.2 |
| ILS AS1-0102 | CC9 | 0.2 | 1.2 |
| ILS AS1-0103 | CC9 | 0.2 | 1.2 |
| ILS AS1-0104 | CC9 | 0.2 | 1.2 |
| ILS AS1-0105 | CC9 | 0.2 | 1.6 |
| ILS AS1-0106 | CC9 | 0.2 | 1.2 |
| ILS AS1-0107 | CC9 | 0.2 | 1.2 |
| ILS AS1-0108 | CC6 | 0.2 | 1.6 |
| ILS AS1-0109 | CC9 | 0.2 | 1.2 |
| ILS AS1-0110 | CC204 | 0.2 | 1.2 |
| ILS AS1-0111 | CC204 | 0.2 | 1.2 |
| ILS AS1-0112 | CC204 | 0.2 | 1.2 |
| ILS AS1-0113 | CC204 | 0.2 | 1.2 |
| ILS AS1-0114 | CC204 | 0.2 | 1.2 |
| ILS AS1-0115 | CC204 | 0.2 | 1.2 |
| ILS AS1-0116 | CC204 | 0.2 | 1.2 |
| ILS AS1-0117 | CC204 | 0.2 | 1.2 |
| ILS AS1-0118 | CC204 | 0.2 | 1.2 |
| ILS AS1-0119 | CC9 | 0.2 | 1.2 |
| ILS AS1-0120 | CC204 | 0.2 | 1.2 |
| ILS AS1-0121 | CC204 | 0.2 | 1.2 |
| ILS AS1-0122 | CC204 | 0.2 | 1.2 |
| ILS AS1-0123 | CC121 | 0.2 | 1.6 |
| ILS AS1-0124 | CC204 | 0.2 | 1.2 |
